# Supplementary material for: The evolution of menopause in toothed whales
Source: Nature. 2024 Mar 13;627(8004):579–85. doi: 10.1038/s41586-024-07159-9 (PMC10954554; doi:10.1038/s41586-024-07159-9)
Supplement: Supplementary file 1 — Supplementary figures, tables, equations, mortality and corpora models and additional data. [file 41586_2024_7159_MOESM1_ESM.pdf]

nature portfolio

https://doi.org/10.1038/s41586-024-07159-9

Supplementary information

The evolution of menopause in toothed whales

In the format provided by the authors and unedited

Title

The evolution of menopause in toothed whales

Authors

Samuel Ellis[1] *

Daniel W Franks[2]

Mia Lybkær Kronborg Nielsen[1]

Michael N Weiss[1,3]

Darren P Croft[1,3]

1. Centre for Research in Animal Behaviour, Department of Psychology, University of Exeter, Exeter, UK
2. Department of Biology, University of York, York, UK
3. Center for Whale Research, Friday Harbor, Washington State, USA

Supplementary Information Guide

Supplementary Title	Description
Supplementary Figures	Supplementary figures supporting results the main text. Contains 7 figures.
Supplementary Tables	Supplementary figures supporting results the main text. Contains 8 tables.
Supplementary 1 Equations	Extended versions of main text equations 1, 2. Including details of the model priors. Additionally including detailed explanation of model parameters.
Supplementary 2 Mortality and Corpora	Plots and parameter tables for the fitted mortality and corpora models. Contains 50 figures and 2 tables.
Supplementary 3 Additional Data Explanation	Further explanation of data used in this study: (1) Phylogeny, (2) Age at Maturity, (3) Size, (4) Survival to maturity. Supported by 4 figures and 4 tables.

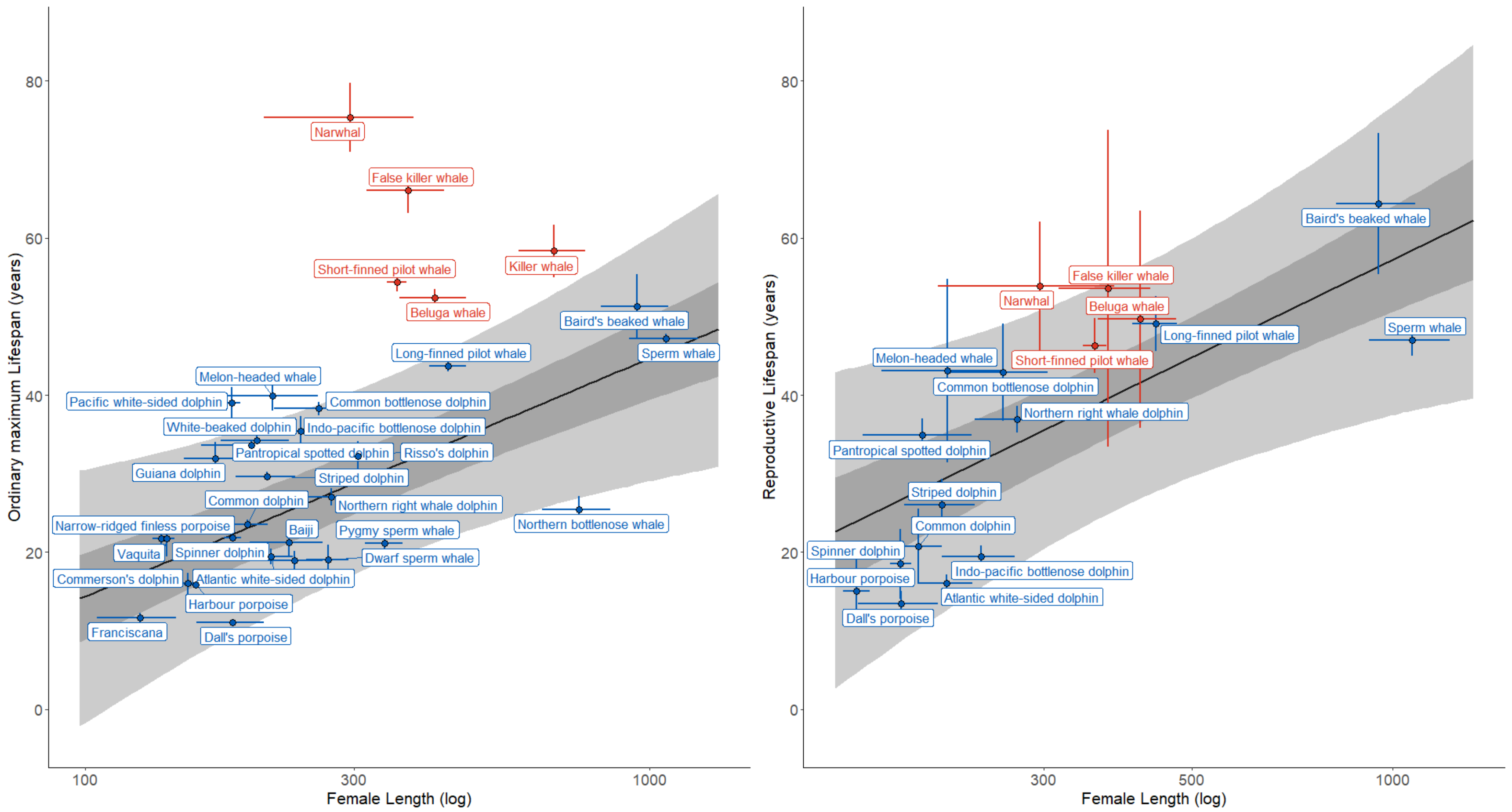

Figure S1. (a) The relationship between body length and ordinary maximum lifespan in female toothed whales with lifespan data (n=32, see figure 1b), and (b) the relationship between reproductive lifespan and body length in toothed whale species with reproductive lifespan data (n=18, see figure 1c). This figure repeats main text figure 1b,c but includes all species labels. For a detailed description of the figures see the legend to main text figure 1.

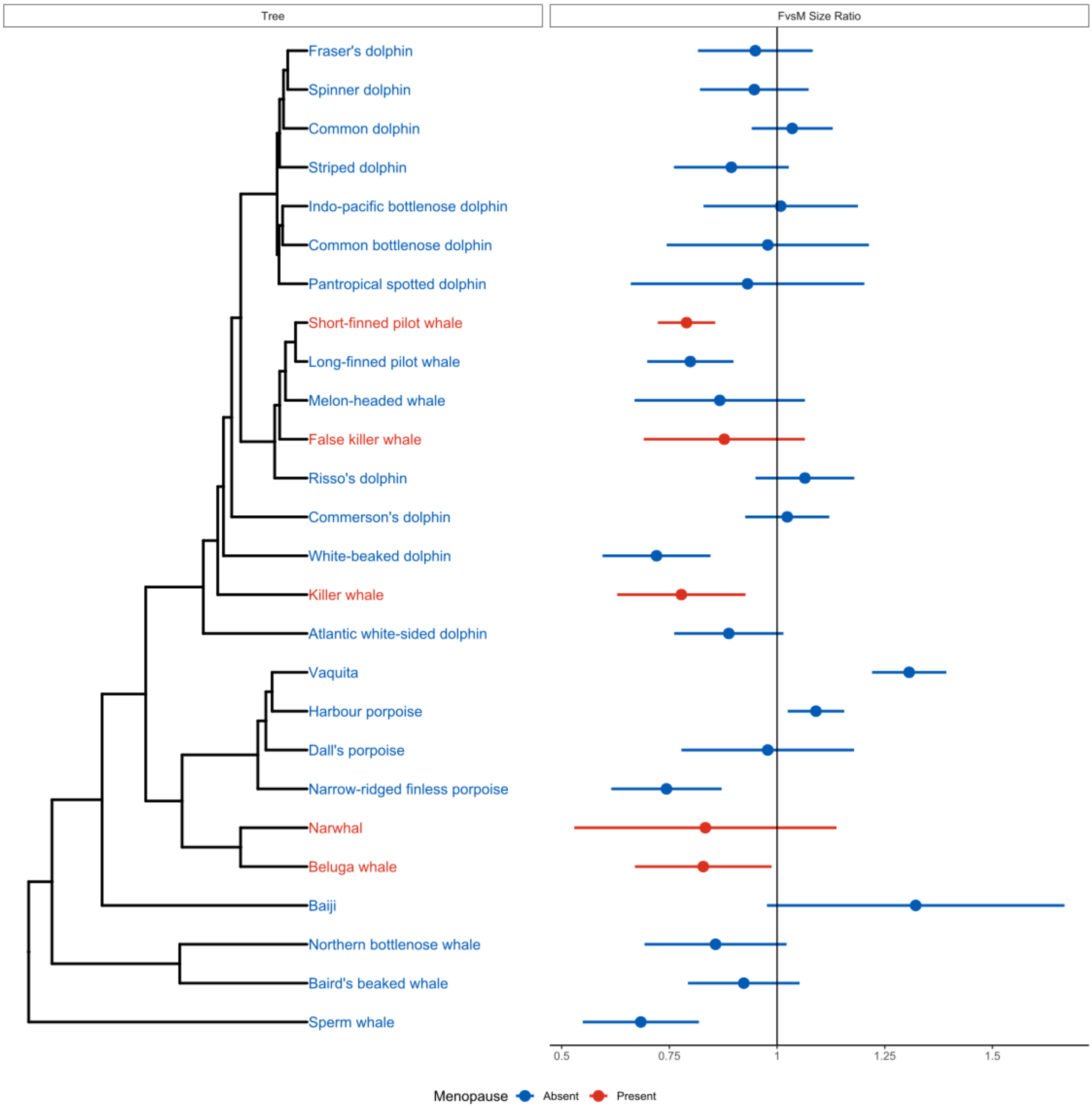

Figure S2. Phylogeny (left panel) and female/male size ratio (right panel) for toothed whales (n=26). Species with menopause (red points, errors and text) have a lower female/male size ratio than species without menopause (blue points, errors and text). For both sexes, size (length) is calculated as mean size with error (standard deviation) around this mean. In the female/male size ratio panel (right), points represent the female size mean estimate / male size mean estimate- with error showing the standard deviation of that estimate calculated from the higher and lower estimates of that ratio based on the error around the estimates.

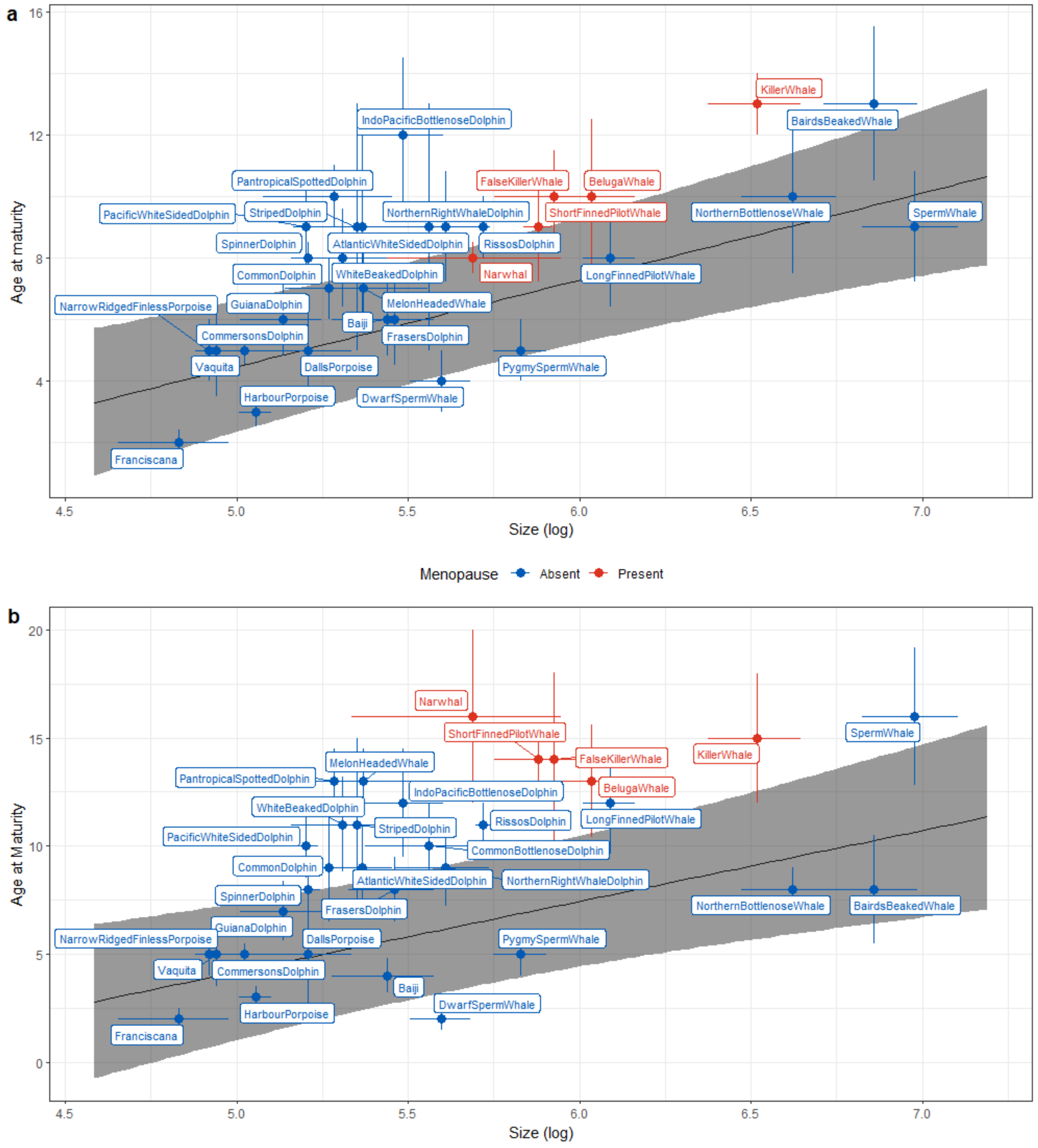

Figure S3 Female (a) and male (b) age at maturity given the size of their mother (female size) for species of toothed whale with (red points and error) and without (blue points and error) menopause (both panels, n=32). Size is measured as length (cm), points show the mean estimated size with variation (standard deviation) around that mean (more details Supplementary Data Explanation). For age at maturity points show the mean age at maturity with error bars showing variation around that mean (more details Supplementary 3). Phylogenetic autocorrelation and variation on both axes are included in the fitted model. Black line and grey ribbon shows the posterior mean and 95% credible interval from models predicting the relationship between female size and offspring age at maturity. Model and outputs described in the main text. Neither the female nor male offspring of species with menopause reach maturity later than expected given their size.

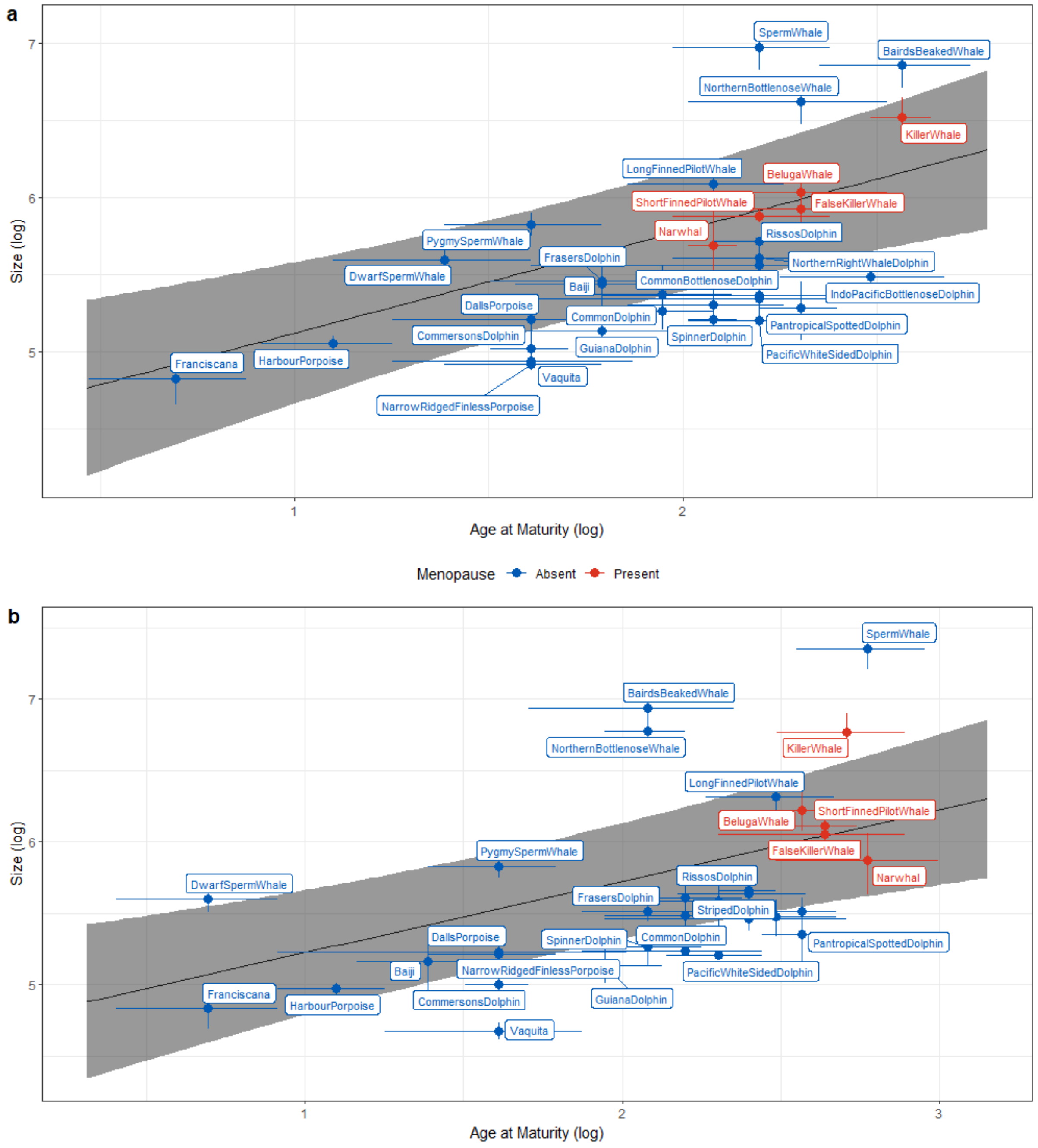

Figure S4. Female (a) and male (b) adult size given their age at maturity for toothed whale species (both panels, n=32) with (red points and error) and without (blue points and error). Size is measured as length (cm), points show the mean estimated size with error (standard deviation) around that estimate (more details Supplementary 3). For age at maturity points show the mean age at maturity with error bars showing variation around that mean (more details Supplementary Data Explanation). Phylogenetic autocorrelation and variation on both axes are included in the fitted model. Black line and grey ribbon shows the posterior mean and 95% credible interval from models predicting the relationship between age at maturity and male and female adult size. Model and outputs described in the main text. Neither females nor males of species with menopause are larger than expected given their age at maturity.

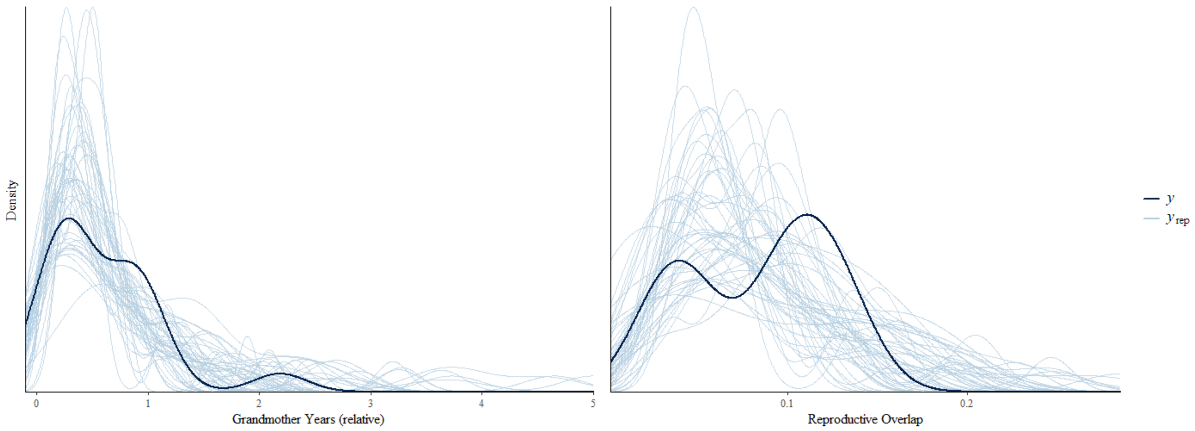

Figure S5. Posterior predictive checks for the Grandmother Years (left) and Reproductive Overlap right) models (main text figure 2). Thick blue lines represent the observed distribution of the parameter (y) and thin blue lines (y_{rep}) show the predicted distribution from 50 posterior draws from the model. In both cases, the model captures the true observed distribution.

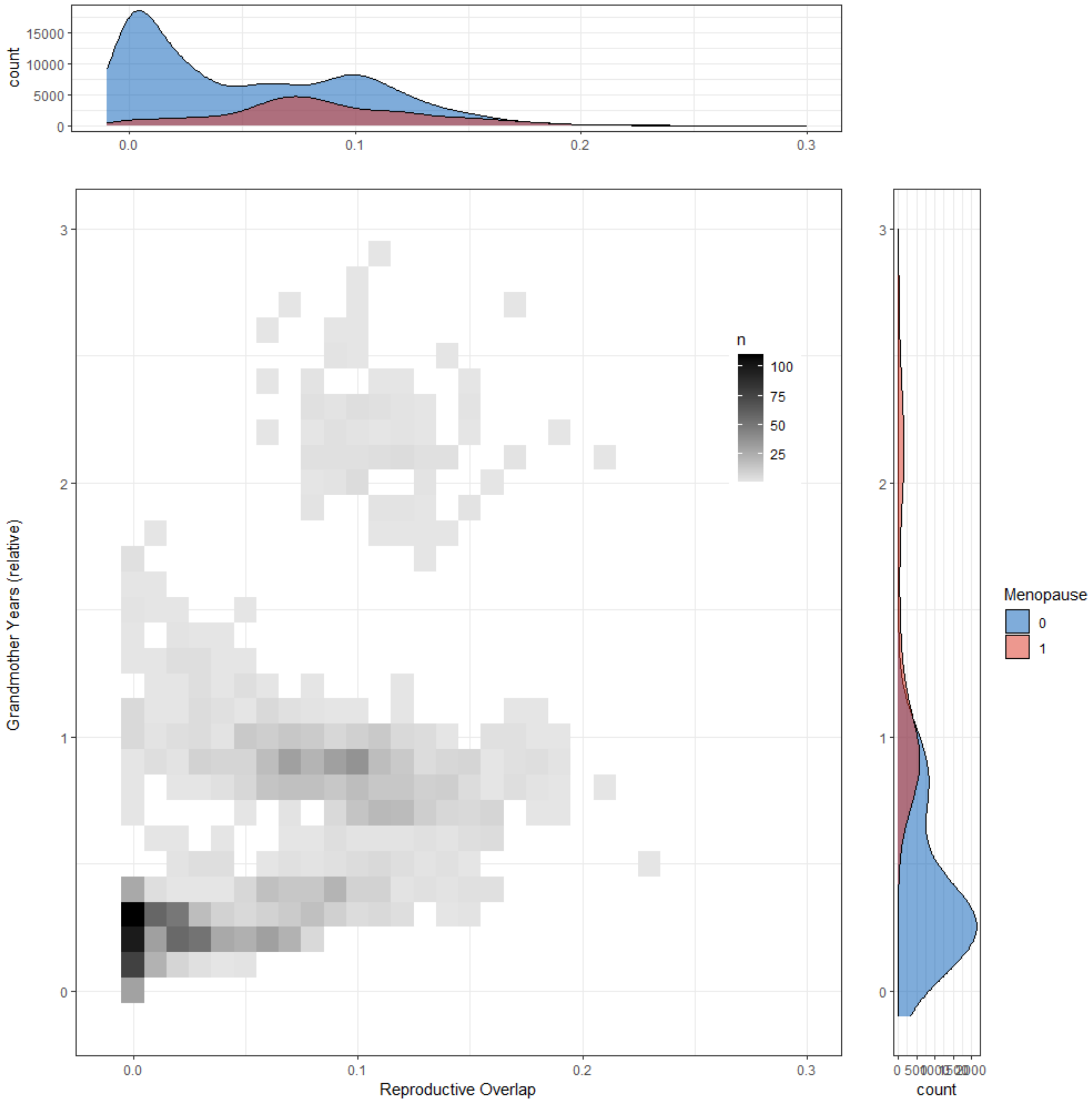

Figure S6. Distributions of the Grandmother Years (relative) and Reproductive Overlap metrics combined for all species. Density plots (top panel, right panel) show the distributions of the posterior draws of the Reproductive Overlap (top panel) and Grandmother Years (right panel) metrics for species with (red) and without (blue) menopause. The heat map shows the combined distribution of the two metrics. The darkness of the a cell represents the number of draws from the combined posterior falling within that cell. Darker colours show more draws are found in that cell. The isolated ‘island’ of draws with very high grandmother years (but typical reproductive overlap) are from Narwal which have very high Grandmother Years (fig 2a).

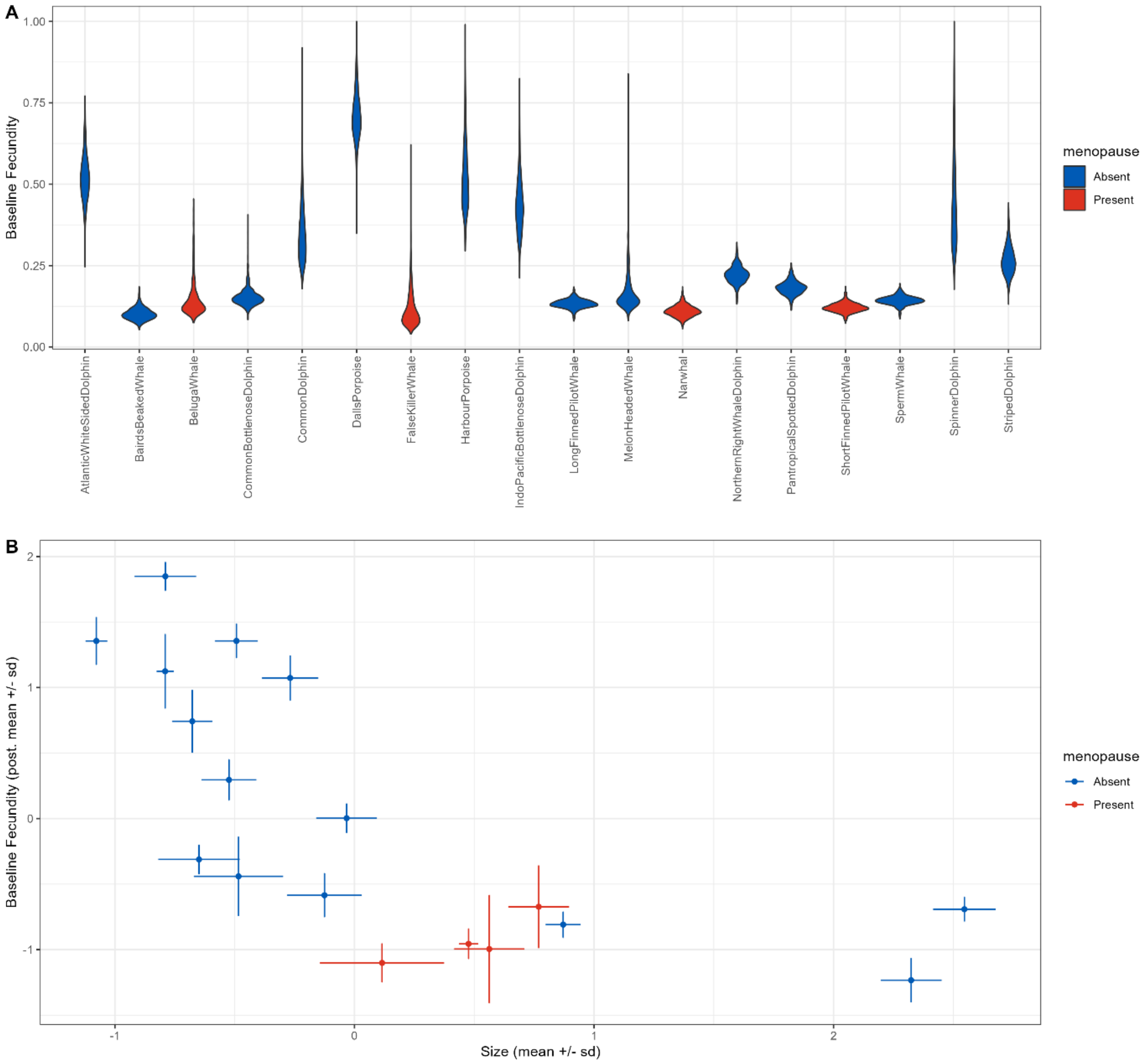

Figure S7. Baseline fecundity in species (n=18) with (red points, lines and violins) and without menopause (blue points, lines and violins). (A) Baseline Fecundity estimates derived from 1000 draws from kinship demography analysis (see methods) for female toothed whales. (B) The relationship between baseline fecundity (z scored) and species size (log and z scored). Species with menopause do not have a baseline rate of reproduction different than expected given their size and phylogenetic position (β = -0.51, 95CI = -0.857 -0.139).

Table S1. Details of the samples used to construct the estimate age-specific mortality for 32 species of toothed whale.

**Species**	**N Datasets**	**Total whales in sample**	**N whales in largest sample**	**Highest sampling ratio (*number of whales in sample / age of oldest whale across all samples*)**
Atlantic white-sided dolphin	4	45	23	2.20
Baiji	1	17	17	1.12
Baird's beaked whale	2	24	22	0.57
Beluga whale	8	757	176	4.15
Commerson's dolphin	2	24	19	1.72
Common bottlenose dolphin	10	423	137	4.68
Common dolphin	6	628	284	17.14
Dall's porpoise	4	854	510	83.33
Dwarf sperm whale	1	15	15	1.00
False killer whale	2	100	66	1.18
Franciscana	7	228	68	7.02
Fraser's dolphin	3	39	30	2.32
Guiana dolphin	6	49	14	0.54
Harbour porpoise	12	643	199	15.46
Indo-pacific bottlenose dolphin	2	75	43	1.83
Killer whale	6	112	45	0.99
Long-finned pilot whale	3	1170	990	27.75
Melon-headed whale	4	90	36	1.10
Narrow-ridged finless porpoise	6	96	28	1.67
Narwhal	3	100	51	0.76
Northern bottlenose whale	1	26	26	1.68
Northern right whale dolphin	2	105	73	4.05
Pacific white-sided dolphin	3	73	46	1.53
Pantropical spotted dolphin	8	1039	275	11.64
Pygmy sperm whale	3	26	16	0.99
Risso's dolphin	4	51	29	1.25
Short-finned pilot whale	3	505	321	7.08
Sperm whale	5	2932	2093	54.78
Spinner dolphin	2	1420	717	51.85
Striped dolphin	2	913	900	43.59
Vaquita	1	11	11	0.66
White-beaked dolphin	3	51	35	1.33

Table S2. Details of the samples used to construct the estimate age-specific fertility estimates for 18 species of toothed whale.

**Species**	**# Datasets**	**Total whales in sample**	**# whales in largest sample**	**Highest sampling ratio (*number of whales in sample / age of oldest whale across all samples*)**
Atlantic white-sided dolphin	1	24	24	2.30
Baird's beaked whale	1	24	24	0.63
Beluga whale	2	184	114	2.69
Common bottlenose dolphin	3	223	100	3.42
Common dolphin	2	349	248	14.96
Dall's porpoise	1	66	66	10.78
False killer whale	2	75	52	0.93
Harbour porpoise	3	113	66	5.13
Indo-pacific bottlenose dolphin	1	31	31	1.32
Long-finned pilot whale	1	574	574	16.09
Melon-headed whale	2	82	48	1.46
Narwhal	1	47	47	0.70
Northern right whale dolphin	1	61	61	3.38
Pantropical spotted dolphin	1	517	517	21.88
Short-finned pilot whale	1	187	187	4.13
Sperm whale	1	792	792	20.73
Spinner dolphin	2	262	133	9.62
Striped dolphin	1	118	118	5.71

Table S3. Derivation of parameters for the demographic simulation analyses.

	Observed Case	Ancestral Case	Slow life-history case
**Adult age-specific mortality**	See *lifespan data and modelling* section	We use 4000 posterior draws from the fitted lifespan vs size model (see *live-long vs stop early* section) to derive lifespan estimates (z) for each of the five species with menopause if βPR is 0 (rather than 1).<br><br>We then fit a further model to each of these 4000 estimates of the form:<br><br>$z = \frac{\log\left(\frac{\alpha}{\beta}(\log(0.1)+1)\right)}{\beta}$<br><br>To get a distribution of estimates of α and β – and hence the pattern of age-specific adult mortality – fitting the required maximum lifespan for each of the five species. Priors for α and β are the same as for mortality model.	As observed case
**Juvenile mortality**	See *Size, age at maturity and survival to maturity* section and Supplementary 3.	As observed case.	We fit a Bayesian phylogenetically controlled linear model to estimate the relationship between lifespan and age at maturity in toothed whales with a term for the presence or absence of menopause (cf. equation 3). We use this model to estimate the expected age at maturity for each species with menopause given their size. We then follow the steps outlined in the *survival to maturity* analysis to get estimates of age-specific juvenile mortality for each of the five species with menopause.
**Age-specific fecundity**	For simplicity, we consider a linear decline in fecundity from age at maturity to the age of the last observed reproduction. We then multiply this linear fecundity by a scaling factor (calculated as *kinship demography section*) to get an estimate of age-specific fecundity. Changing the assumption of linear decline to quadratic or to constant fecundity does not qualitatively change the results.	As observed case.	From our database, we found the age of last known reproduction for each of the 18 species for which we had corpora reproductive data. Fitting a model of age at last reproduction given lifespan reveals that, as expected, species with menopause have an earlier age of last known reproduction given their lifespan. We use this regression to get a distribution of expected age at last reproduction for each species with menopause if they did not have menopause. With these predicted ages of last reproduction, we then follow the pathway outline in *kinship demography analysis* section to get simulated estimates of age-specific fecundity.

Table S4. Age of last observed reproduction for the five toothed whale species with menopause. Reproductive evidence type indicates how reproduction was inferred for the oldest sample: pregnancy are the oldest known pregnant individual; all corpora is the end of any kind of ovarian corpora activity; and corpora lutea are the oldest known active corpora lutea. Where a species has multiple reproductive evidence types the oldest age (*) is used.

**Species**	**Age of oldest observed reproductively active female**	**Reproductive evidence type**	**Reference**
Beluga whale	34	all corpora	[1]
Beluga whale	41*	corpora lutea	[2]
False killer whale	39*	pregnancy	[3]
Killer whale	41*	pregnancy	[4]
Narwhal	47	all corpora	[1]
Narwhal	69*	pregnancy	[5]
Short-finned pilot whale	38	all corpora	[1]
Short-finned pilot whale	39*	corpora lutea	[6]
Short-finned pilot whale	36	pregnancy	[6]

Table S5. Key results from the analysis repeated excluding particular species or groups of species. *None* represents no-exclusions and repeats the result from the main text (but see *). *Corpora species only* repeats the analysis using only species for which reproductive lifespan are available. *PrR with error column* repeats the analysis with the binary 0/1 indicator of menopause replaced with the published Post-Reproductive Representation (PrR) values for a given species (table S8). PrR represents the proportion of adult female life years lived by post-reproductive females[7]. In the analysis we consider true PrR to be drawn from a normal distribution centred on the published PrR estimates, with a standard deviation of half the published range of PrR values for that species (therefore assuming the published range encompasses 95% of the true variation). *Without strandings samples* shows the results of analysis where the 27/269 datasets from ad-hoc strandings data are excluded from the mortality modelling (see Supplementary 2). All other columns list the excluded species in the header. No exclusions had any qualitative or a biologically significant quantitative difference to the results.

	**None**	**Corpora species only**	**Beluga whales excluded**	**Narwhals excluded**	**Beluga whales and narwhals excluded**	**False killer whales excluded**	**Sperm whales Excluded**	**Sperm whales and Baird' s beaked whales excluded**	**Sperm whales, Baird' s beaked whales and N. bottlenose whales excluded**	**Vaquita and baiji excluded**	**PrR with error**	**Without strandings samples**
**Proportion of posterior where species with menopause live longer than species without menopause***	0.99	0.98	0.99	0.99	0.97	0.98	0.99	0.99	0.99	0.99	0.94	0.99
**Proportion of posterior where species with menopause reproduce for longer than species without menopause***	0.75	-·	0.76	0.76	0.78	0.75	0.75	0.77	- †	- ‡	0.77	-
**Proportion of posterior where species with menopause spend longer alive at the same time as their grandoffspring than species without menopause**	0.99	-·	0.98	0.96	0.89	0.97	0.99	0.99	-†	- ‡	0.97	0.99
**Proportion of posterior where female/male adult size ratio is smaller in species with**	0.95	-·	0.95	0.95	0.92	0.95	0.97	0.97	0.97	0.95	0.92	-

**menopause than species without menopause**												
**Proportion of posterior where species with menopause have greater reproductive overlap than species without menopause**	0.73	-•	0.71	0.64	0.63	0.75	0.76	0.73	-†	- ǂ	0.58	0.73

*Note: For computational reasons, these models are only run on the consensus tree rather than over multiple tree versions as in the main text. We repeat the analysis reported in the text on a consensus tree to make it comparable (but it may differ slightly from that reported in the text).

• These analyses already only include corpora species so are identical to 'none'

† There is no measure of reproductive lifespan available Northern bottlenose whales therefore analysis unchanged from 'Sperm whales and Baird's beaked whales excluded' case.

ǂ There is no measure of reproductive lifespan available for either vaquita or baiji and therefore analysis is unchanged from 'none'.

Table S6. Comparison of the predictive power of models of female reproductive lifespan (figure 1c, and text) with and without a menopause parameter. In our analysis (figure 1c) we found no evidence that species with menopause have a longer reproductive lifespan than expected given their size. To confirm this result we compare the predictive power of models of reproductive lifespan with and without a menopause term. If menopause where to be important in explaining differences in reproductive lifespan we would expect that model with a menopause parameter will have a greater predictive power than a model without a menopause parameter- even where a lack of power prevented unambiguous estimate of the menopause parameter as above 0.

Predictive power is estimated using leave-one-out-cross-validation implemented in the loo package in R[8]. Expected log pointwise predictive density (elpd) is an estimate of the predictive power of a model- the difference in elpd describes the comparative predictive power, with higher values indicating greater predictive power. Models with a menopause parameter did not have a greater predictive power than models without a menopause parameter- supporting the reported result that species with menopause do not have longer reproductive lifespan than expected given their size.

Model	*elpd difference*	*elpd difference standard error*
Without menopause parameter	0	0
With menopause parameter	-0.8	1.2

Table S7. Comparison of the predictive power of models of toothed whale reproductive overlap (figure 2a, and text) with and without a menopause parameter. In our analysis (figure 2b) we found no evidence that species with menopause have a higher reproductive overlap than species without menopause. To confirm this result we compare the predictive power of models of reproductive overlap with and without a menopause term. If menopause where to be important in explaining differences in reproductive overlap we would expect that model with a menopause parameter will have a greater predictive power than a model without a menopause parameter- even where a lack of power prevented unambiguous estimate of the menopause parameter as above 0.

Predictive power is estimated using leave-one-out-cross-validation implemented in the loo package[8] in R. Expected log pointwise predictive density (elpd) is an estimate of the predictive power of a model- the difference in elpd describes the comparative predictive power, with higher values indicating greater predictive power. Models with a menopause parameter have a lower predictive power than models without a menopause parameter- supporting the reported result that species with menopause do not have higher reproductive overlap than species without menopause.

Model	*elpd difference*	*elpd difference standard error*
Without menopause parameter	0	0
With menopause parameter	-0.4	1.0

Table S8. Published post-reproductive representation (PrR) estimates for toothed whales. Post-reproductive representation calculates the proportion of adult female life being lived by post-reproductive females. Different papers have used slightly different reporting metrics but the data represented is equivalent. 'PrR' values is the mean/best guess PrR estimate for a given species. Minimum PrR is the lowest PrR value reported for a population (given the simulated assumptions used in the paper), and the Max PrR is the highest value.

Species	PrR	Min PrR	Max PrR	Ref.
**Baird's Beaked Whale**	0.01	0	0.02	[9]
**Beluga Whale**	0.24	0.19	0.33	[9]
**False Killer Whale**	0.12	0.09	0.14	[10]
**Long-finned Pilot Whale**	0.01	0	0.02	[9]
**Narwhal**	0.24	0.19	0.29	[9]
**Northern Right-whale Dolphin**	0.03	0.02	0.05	[9]
**Pantropical Spotted Dolphin**	0.02	0.01	0.03	[9]
**Short-finned Pilot Whale**	0.15	0.08	0.22	[9]
**Sperm Whale**	0	0	0.01	[9]
**Spinner Dolphin**	0.01	0.01	0.02	[9]
**Killer Whale**	0.3	0.24	0.37	[4]

Supplementary Table References

1. Ellis, S. *et al.* Analyses of ovarian activity reveal repeated evolution of post-reproductive lifespans in toothed whales. *Scientific Reports* **8**, 1–10 (2018).
2. Suydam, R. S. Age, growth, reproduction, and movements of beluga whales (Delphinapterus leucas) from the eastern Chukchi Sea, Robert Scott Suydam. (University of Washington, 2009).
3. Photopoulou, T., Ferreira, I. M., Best, P. B., Kasuya, T. & Marsh, H. Evidence for a postreproductive phase in female false killer whales Pseudorca crassidens. *Frontiers in Zoology* **14**, 1–14 (2017).
4. Nielsen, M. L. K. *et al.* A long postreproductive life span is a shared trait among genetically distinct killer whale populations. *Ecology and Evolution* **11**, 9123–9136 (2021).
5. Garde, E. *et al.* Life history parameters of narwhals (Monodon monoceros) from Greenland. *Journal of Mammalogy* **96**, 866–879 (2015).
6. Kasuya, T. & Marsh, H. Life history and reproductive biology of the short-finned pilot whale, Globicephala macrorhynchus, off the Pacific coast of Japan. *Report of the International Whaling Commission (Special Issue 6)* 259–310 Preprint at (1984).
7. Levitis, D. A. & Lackey, L. B. A measure for describing and comparing postreproductive life span as a population trait. *Methods in Ecology and Evolution* **2**, 446–453 (2011).
8. Vehtari, A. *et al.* loo: Efficient leave-one-out cross-validation and WAIC for Bayesian models. (2023).
9. Ellis, S. *et al.* Analyses of ovarian activity reveal repeated evolution of post-reproductive lifespans in toothed whales. *Scientific Reports* **8**, 1–10 (2018).
10. Photopoulou, T., Ferreira, I. M., Best, P. B., Kasuya, T. & Marsh, H. Evidence for a postreproductive phase in female false killer whales *Pseudorca crassidens*. *Frontiers in Zoology* **14**, 1–14 (2017).

Supplementary 1: Equations

Sam Ellis

07/04/2022

Equation S1

Complete lifespan model wiht priors and paramter explanations

$$
\begin{aligned}
c &\sim Multinomial(\theta, N) \\
\theta_{d,i} &= \frac{L_{d,i} R_{d,i} S_{d,i}}{\sum L_{d,i} R_{d,i} S_{d,i}} \\
L_{d,i} &= \frac{e^{-(\alpha_{SEX_d}/\beta_{SEX_d})(e^{\beta_{SEX_d}{}^{AGE_{d,i}}}-1)}}{\sum_{j=0}^{n} e^{-(\alpha_{SEX_d}/\beta_{SEX_d})(e^{\beta_{SEX_d}{}^{j}}-1)}} \\
R_{d,i} &= (1-\rho_d)^{AGE_{d,i}} \\
\rho_d &= \frac{r_{POP_d}}{\frac{1}{2} max(o_{DSET_{SEX=SEX_d}})} \\
S_{d,i} &\sim \begin{cases} s_d + 1\,, & AGE_{d,i} \in W_d \\ 1\,, & AGE_{d,i} \notin W_d \end{cases}
\end{aligned}
$$

$$
\begin{aligned}
\tau_j &\sim Normal(o_j, \epsilon_j) \\
\epsilon_j &= \frac{1}{20}(o_j + B) \\
c_{d,i} &= \sum_{k=1}^{N} [\lfloor \tau_k \rceil = AGE_i, DSET_k = d]
\end{aligned}
$$

$$
\begin{aligned}
\alpha_2 &\sim Beta(p\alpha_1, p\alpha_2) \\
\beta_2 &\sim Beta(p\beta_1, p\beta_2) \\
r_p \in [-0.5, 0.5] &\sim \begin{cases} Normal(-0.25, 0.2)\,, & pr_p = 1 \\ Normal(0, 1)\,, & pr_p = 0 \\ Normal(0.25, 0.2), & pr_p = -1 \end{cases} \\
s_d \in [-1, 2] &\sim \begin{cases} Normal(1, 0.5)\,, & ps_d = 1 \\ Normal(0, 2)\,, & ps_d = 0 \\ Normal(-1, 0.5), & ps_d = -1 \end{cases}
\end{aligned}
$$

Where:

Parameters

- α Gompertez mortality model baseline mortality parameter
- β Gompertez mortality model ageing parameter
- τ True age of observed whale parameter
- r population growth parameter
- s sampling bias parameter

Inputs

- o observed ages of sampled whales
- SEX vector indicating the sexes of whales in each dataset [each dataset is single sex]
- AGE vector of possible ages of whales in the sample: 0-150. [where 0 = age at maturity]
- POP vector indicating the population each dataset is drawn from.
- $DSET$ vector indicating which dataset each sample (observed age o) is drawn from
- W Range of ages ('Window') with potential sampling bias in each dataset
- B Age of sexual maturity
- $p\alpha$, $ps\beta$ shape parameters for prior on the gompertez parameters. Each is of length 2.
- pr prior belief on the direction of population growth for each population
- ps prior belief on the direction of bias on samples with in the window W for each dataset

Modelling Variables

- i index through possible ages
- d index through datasets
- k index through samples
- p index through populations
- n total number of possible ages
- N total number of samples [dead whales]
- $c_{d,i}$ count of samples [dead whales] in dataset d of age i
- $L_{d,i}$ Probability of surviving to age i given dataset d [which is actually just a way of indicating sex]
- $R_{d.i}$ Change in probability of finding a whale of age i given population growth of dataset d
- $S_{d,i}$ Change in probability of finding a whale of age i in dataset d due to sampling bias.
- ρ_d change in population size over half the maximum lifespan of the whales in dataset d
- ϵ_j standard deviation around observed age of maturity

Equation S2

Modelling corpora data, full model and parameter list.

$$C \sim Poisson(\lambda)$$

$$\lambda_i = \sum_{j=0}^{i} \Delta k_j$$

$$\Delta k_j = \alpha(1 - \beta AGE_j)$$

$$\alpha \in \mathbb{R}_{\geq 0} \sim Normal(0, 1)$$

$$\beta \sim Beta(b_1, b_2)$$

Parameters

- α Initial annual corpora deposition rate
- β Rate of decline in corpora deposition with age

Inputs

- C Total number of corpora
- AGE Relative age (where 0 = age at maturity)
- b shape priors on β . Vector of length 2. Prior assumption is that reproductive lifespan ends with somatic lifespan. Where maximum lifespan is age Z from the lifespan model with error.

Modelling Parameters

- i index through all samples *λ_i Mean corpora at age i
- δc_i Additional corpora added at age i

Supplementary 2: Mortality and Corpora data and models

Supplementary data for the fitted mortality and corpora models. A. Fitted Mortality Models, B. Fitted Corpora Models, C. Fitted Mortality Models: Parameter table, D. Fitted Corpora Models: Parameter table.

A. Fitted Mortality Models

Mortality models fitted to toothed whale datasets used in this study (see text and equations 1 + S1)..

Models are run on all datasets for a species simultaneously, with some parameters shred between datasets/groups of datasets and other applying only to one dataset (see methods). These plots show the fitted mortality models for female data of each species. For each species, each panel represents one dataset- with grey bars showing counts of whales in that age category, and the red line showing the model estimated posterior mean of multinomial distribution for a given age category (ribbon 95% credible interval around the mean). 27 /269 datasets from 8 species come from ad-hoc stranding events: datasets 47, 78, 49, 50, 51, 77, 78, 206, 207, 200, 201, 228, 229, 242, 243, 250, 251, 253, 254, 255, 256, 257, 25, 259, 260, 261. We define ad-hoc strandings events as from a collection of individual strandings data rather than a single stranding event (or other source). The datasets do not have a noticeably different age distribution to datasets from other sources so are treated in the same way as other datasets. However, removing these datasets makes little difference to to our estimates of age-specific survival, and running the rest of our analysis using mortality models without these datasets does not qualitatively affect our results (table S5).

AtlanticWhiteSidedDolphin

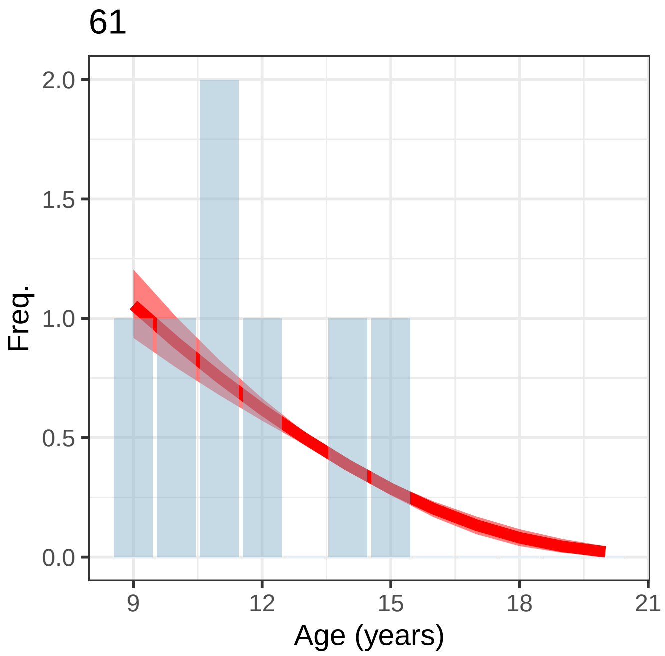

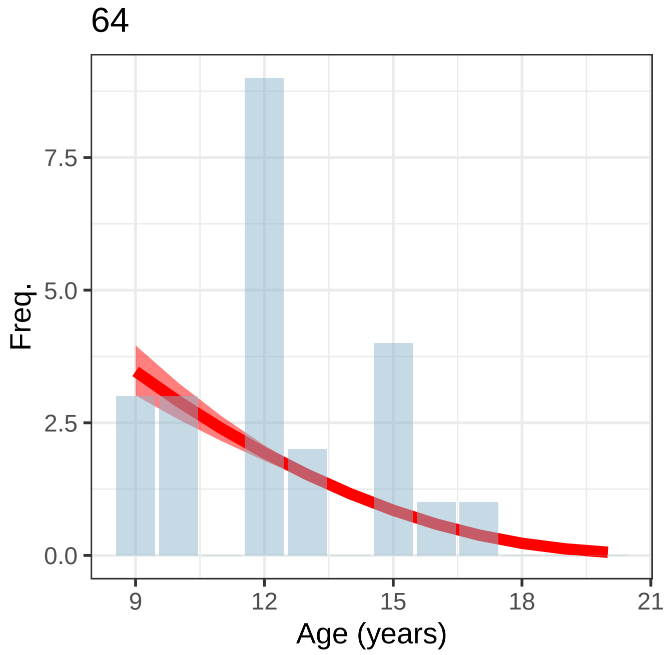

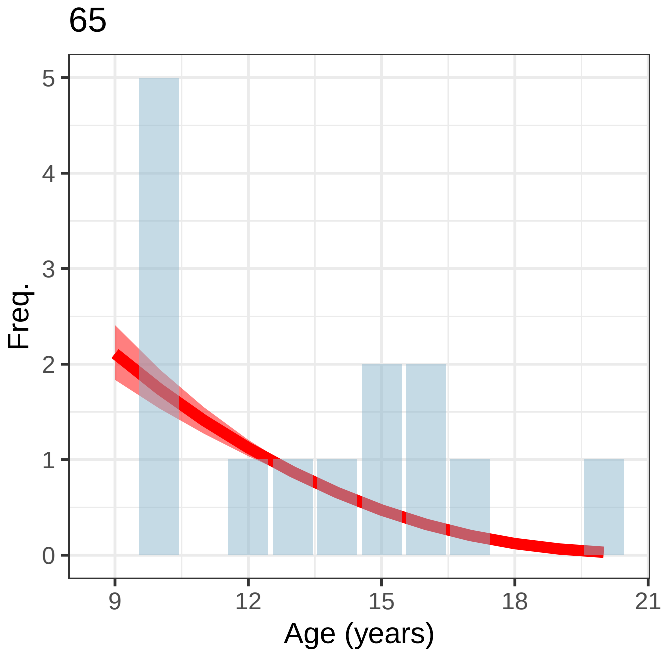

Baiji

89

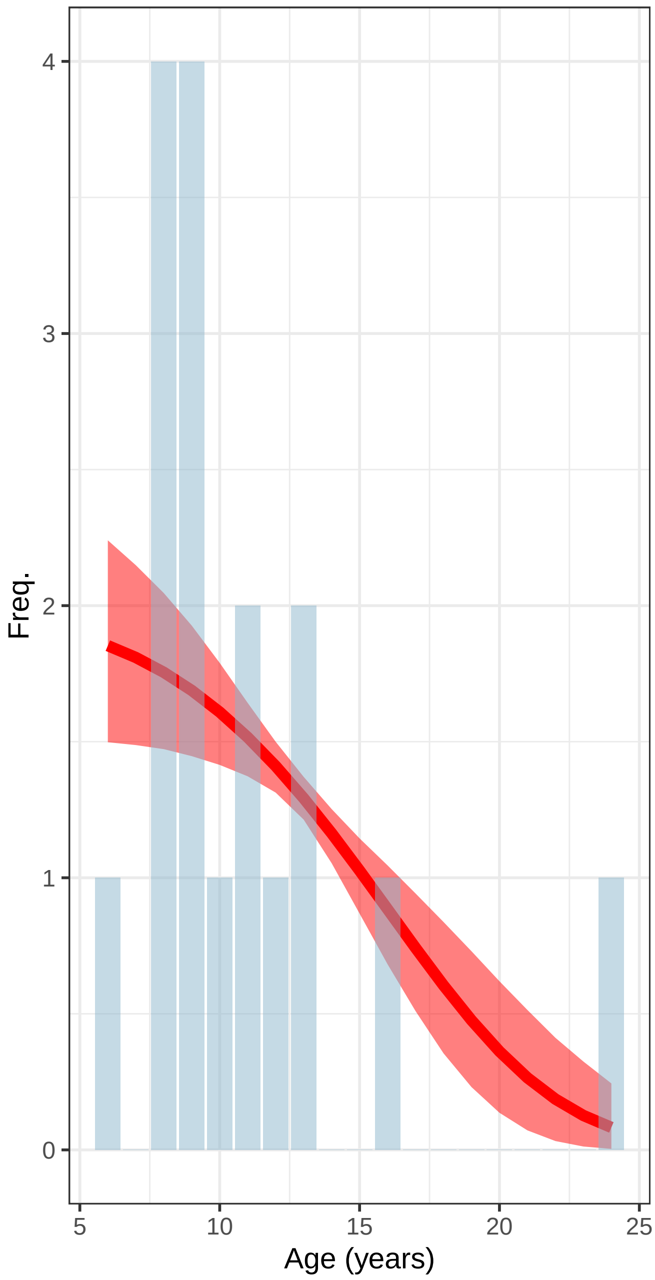

BairdsBeakedWhale

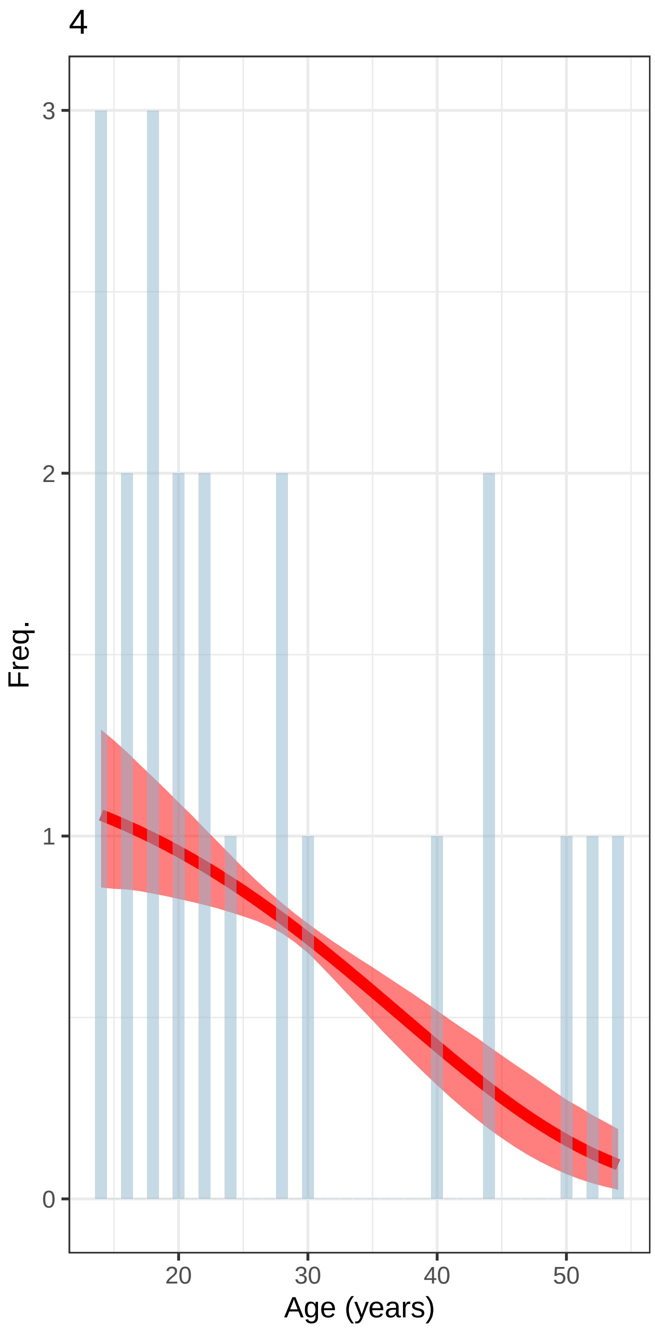

BelugaWhale

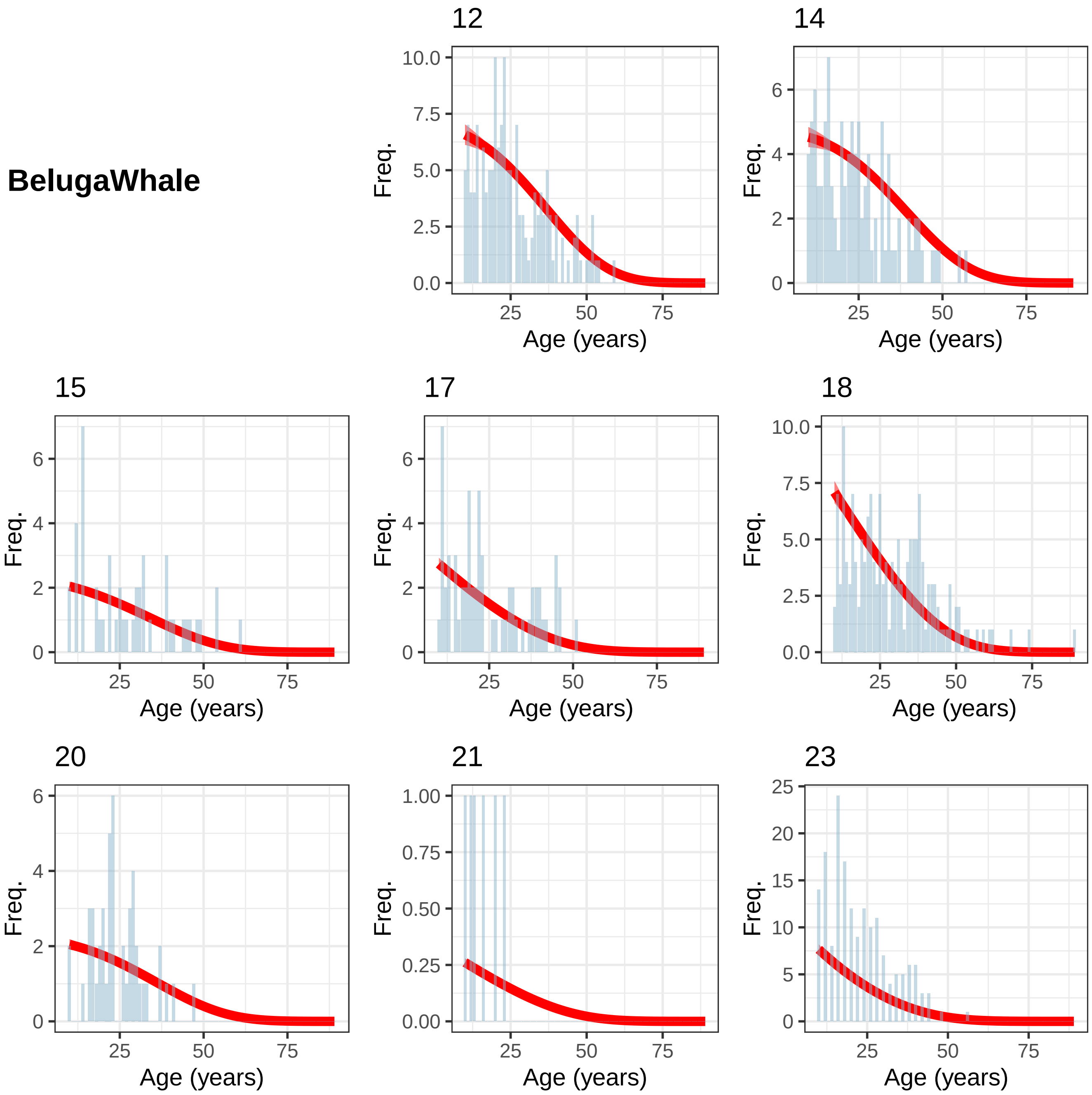

CommersonsDolphin

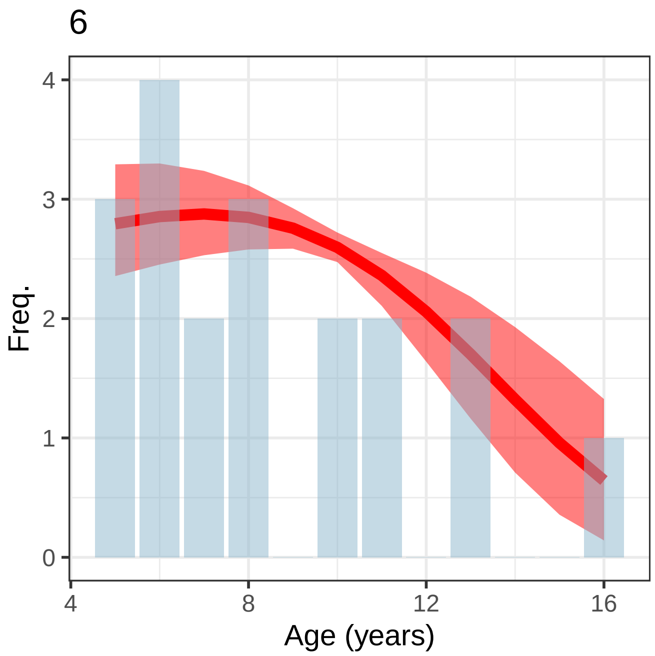

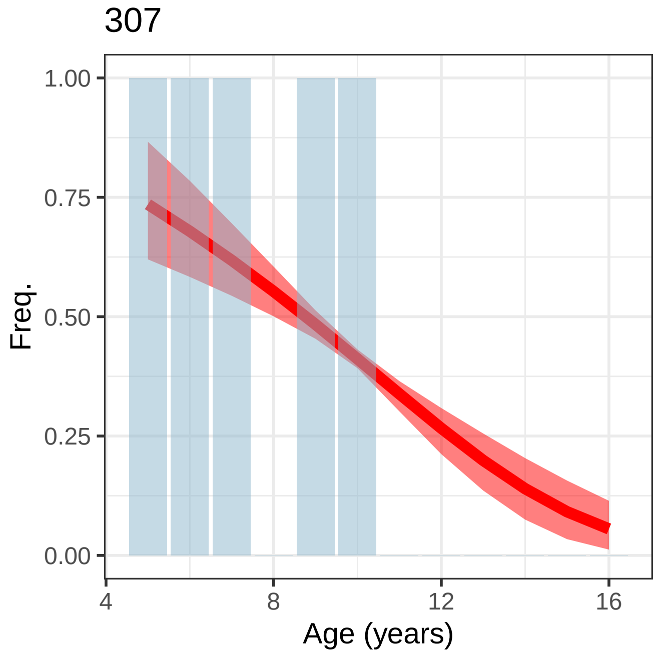

CommonBottlenos

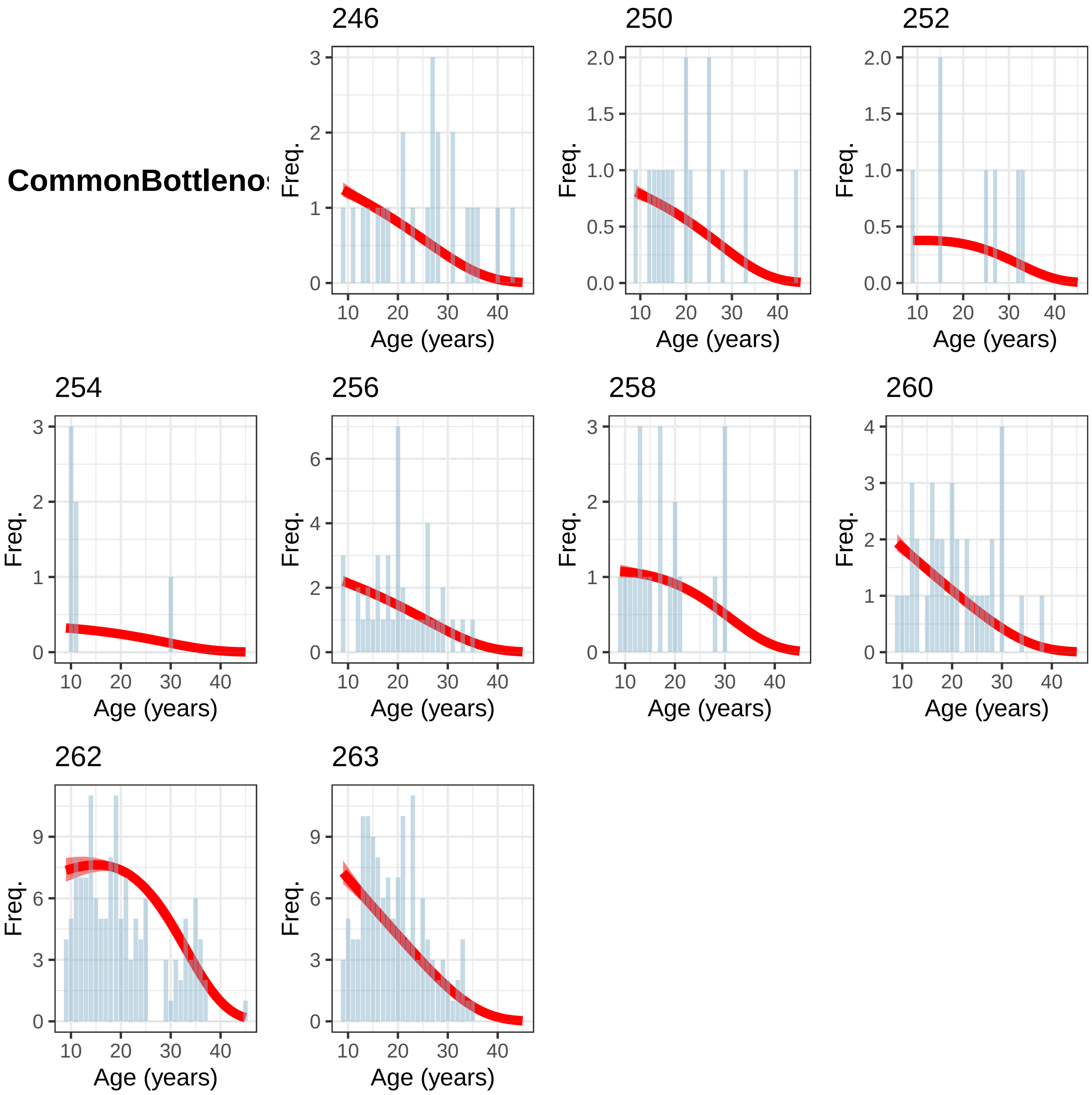

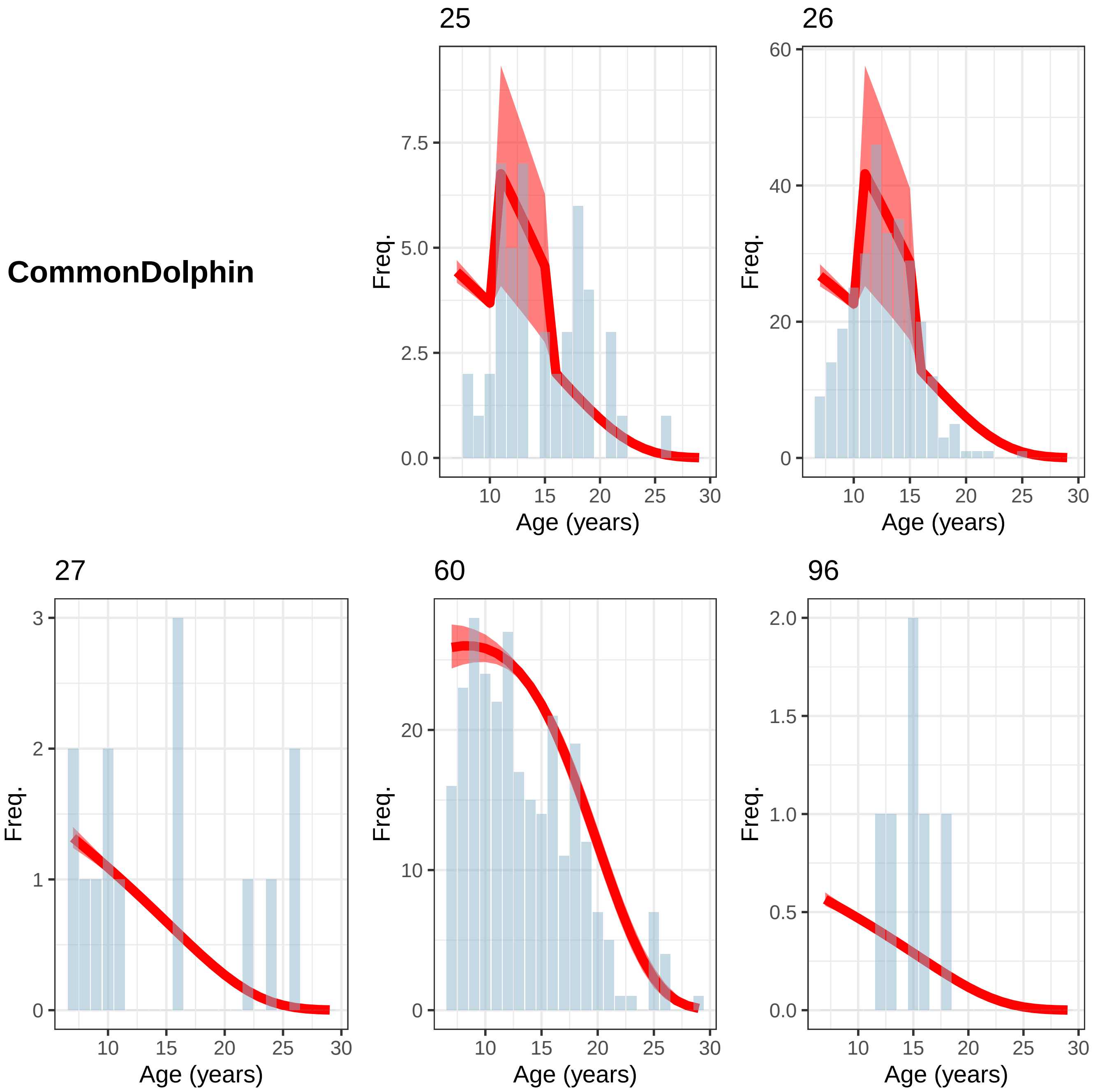

CommonDolphin
25
26
27
60
96
Freq.
Age (years)
0.0
2.5
5.0
7.5
0
20
40
60
0
1
2
3
0
10
20
0.0
0.5
1.0
1.5
2.0
10
15
20
25
30

DallsPorpoise

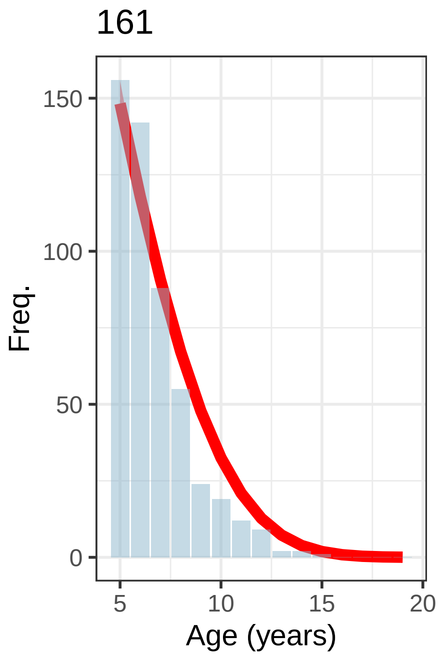

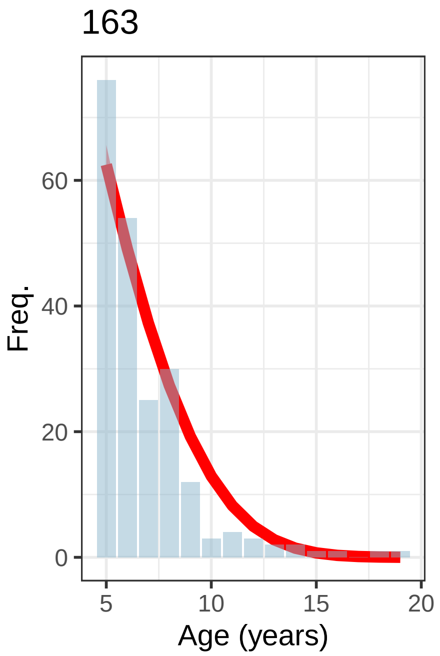

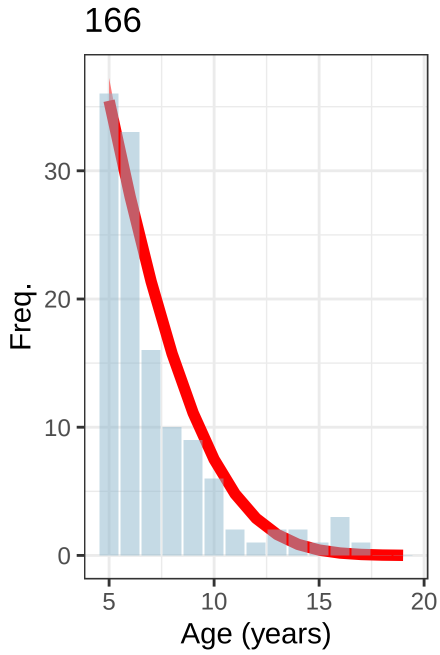

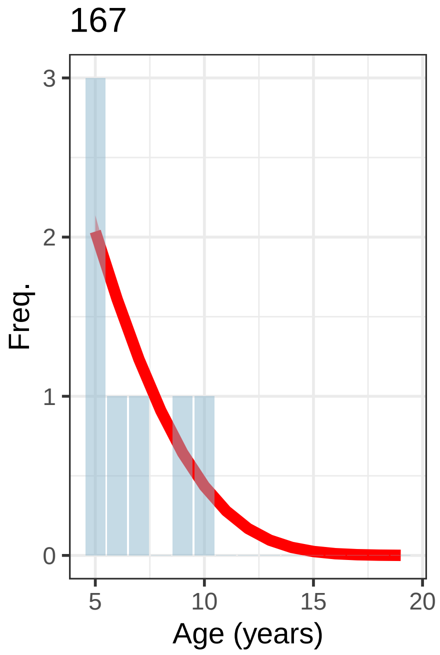

DwarfSpermWhale

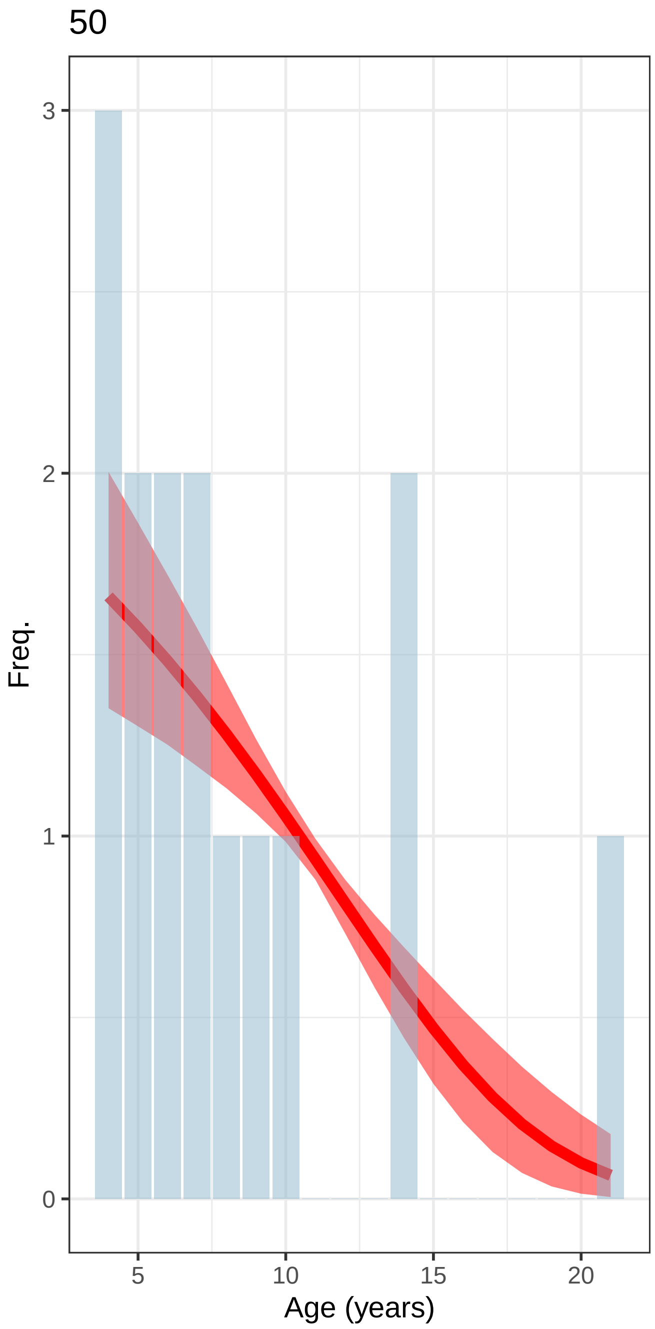

FalseKillerWhale

194

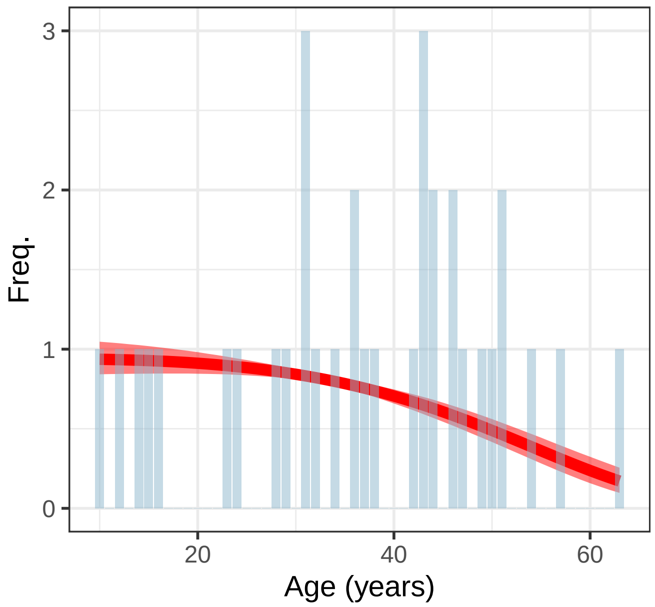

196

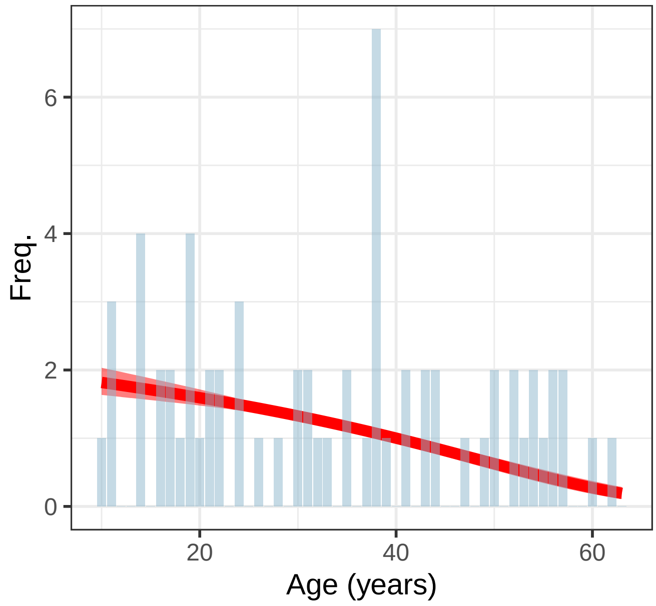

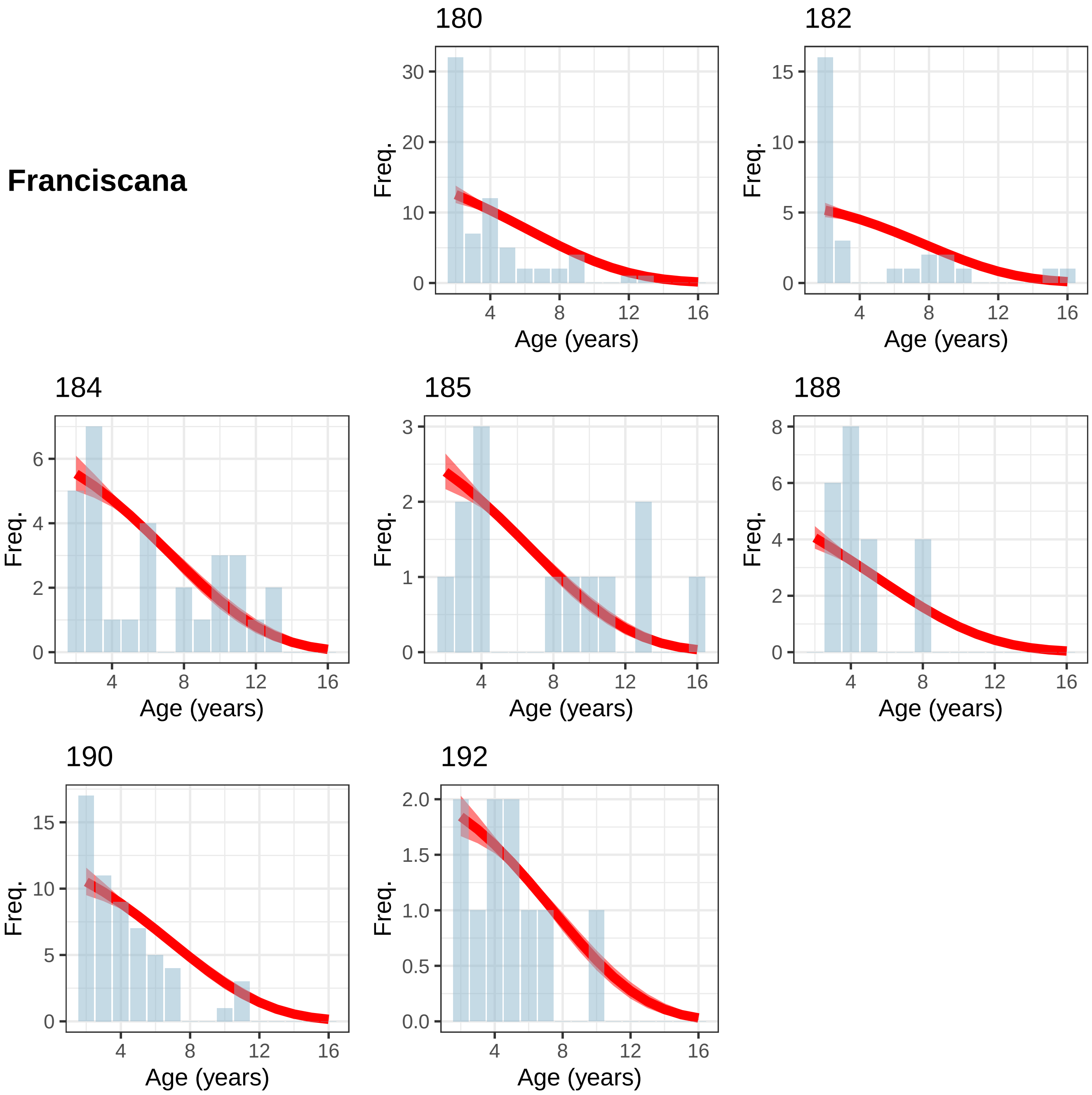
Franciscana
180
182
184
185
188
190
192
Freq.
Age (years)

FrasersDolphin

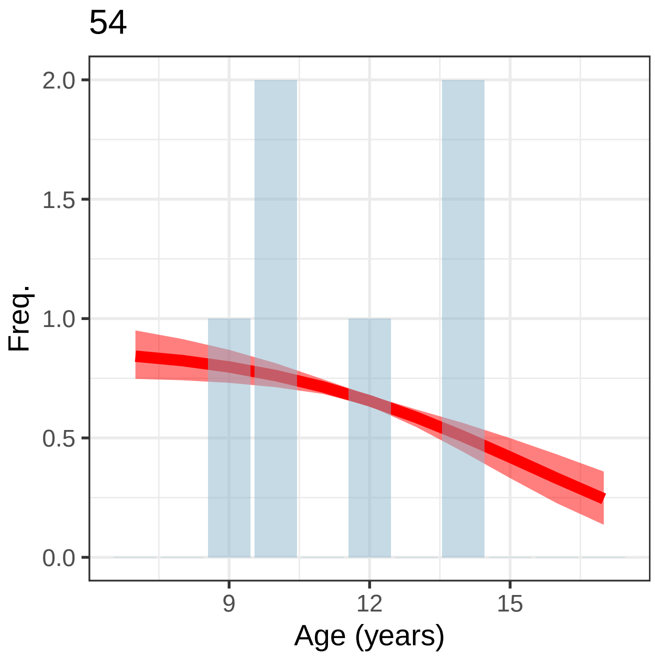

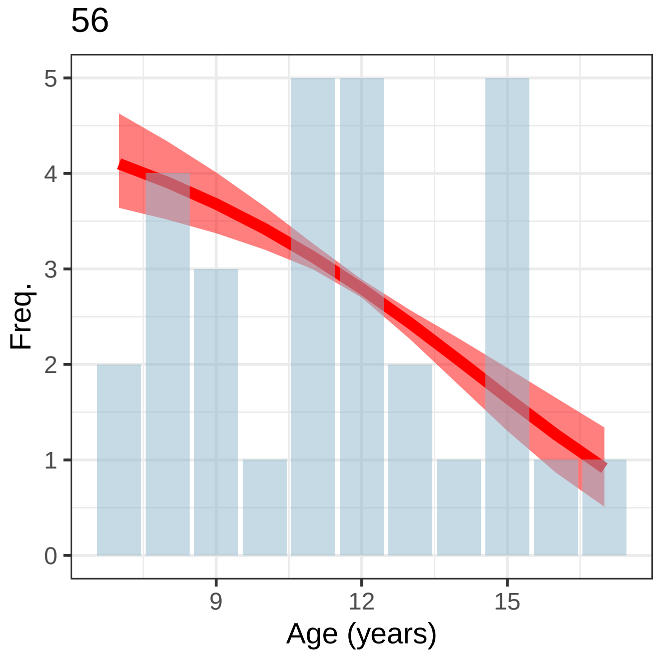

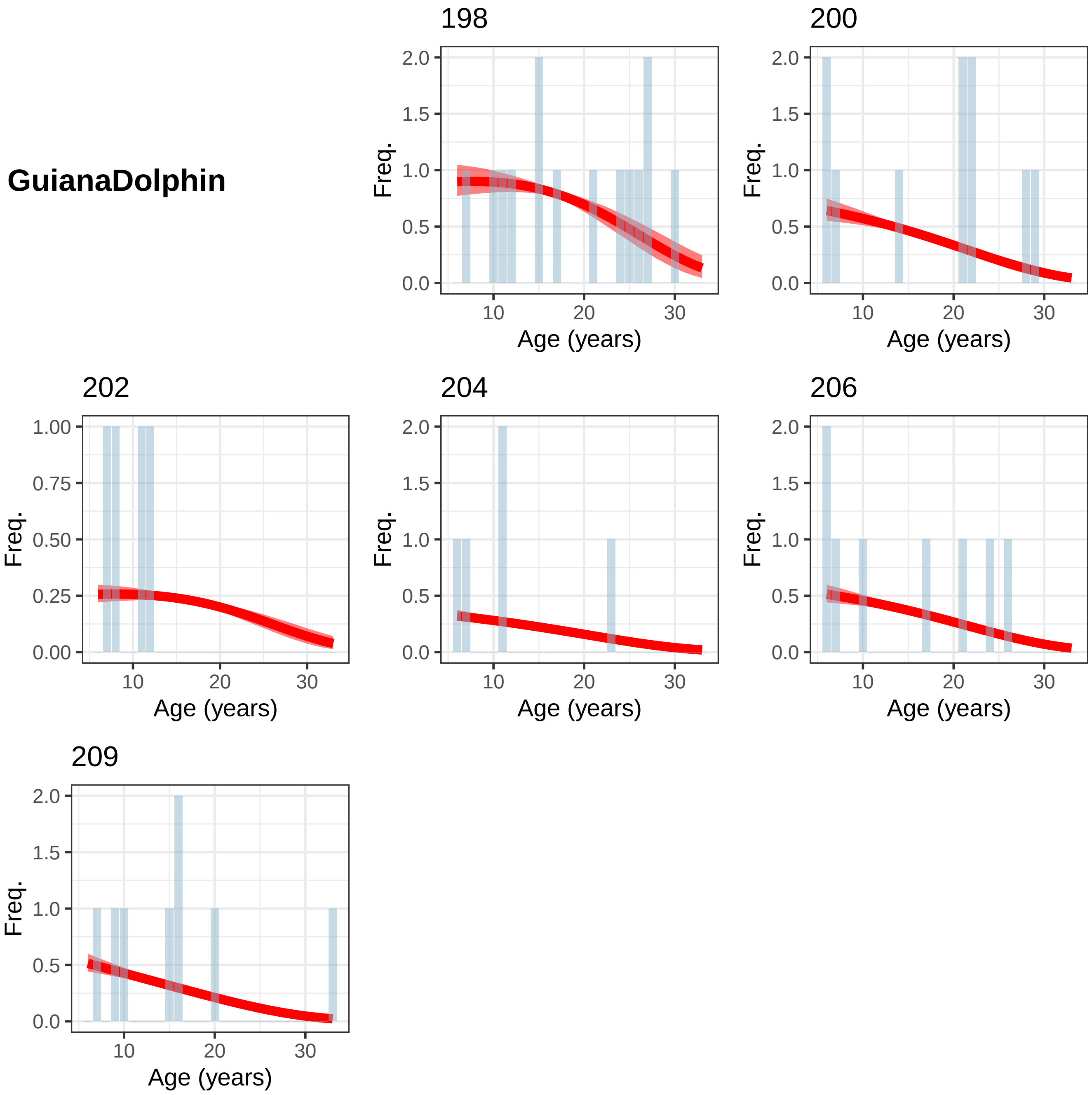
GuianaDolphin
198
200
202
204
206
209
Freq.
Age (years)

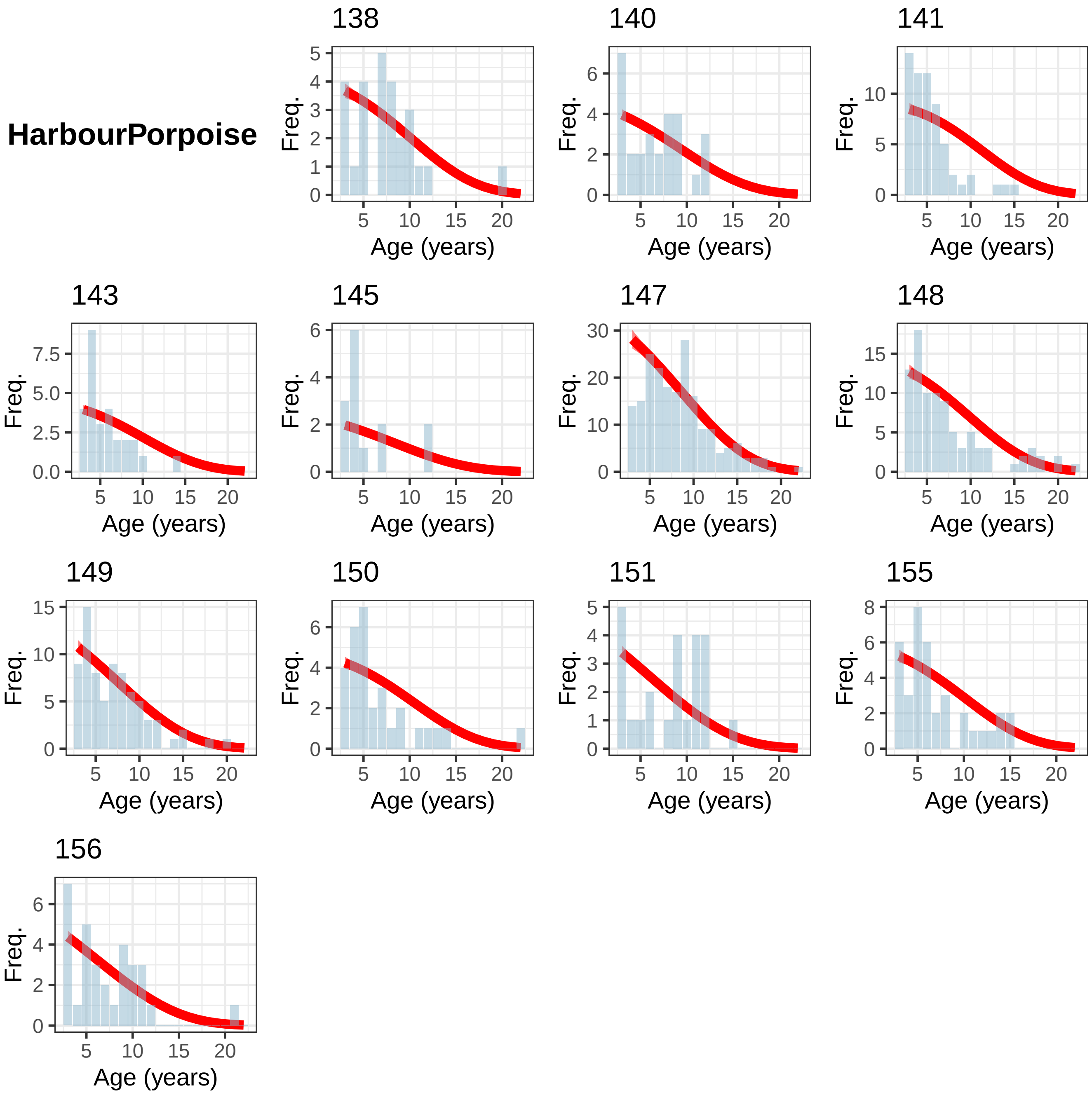

HarbourPorpoise
138
140
141
143
145
147
148
149
150
151
155
156
Freq.
Age (years)

IndoPacificBottlenoseDolphin

243

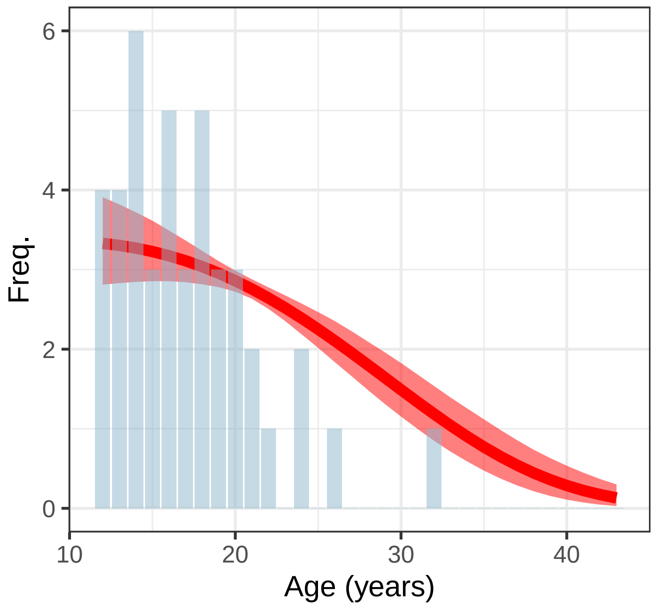

244

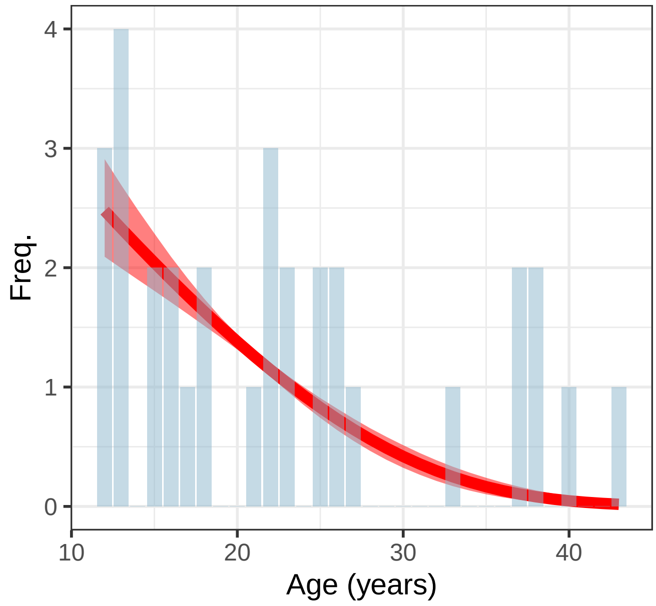

KillerWhale

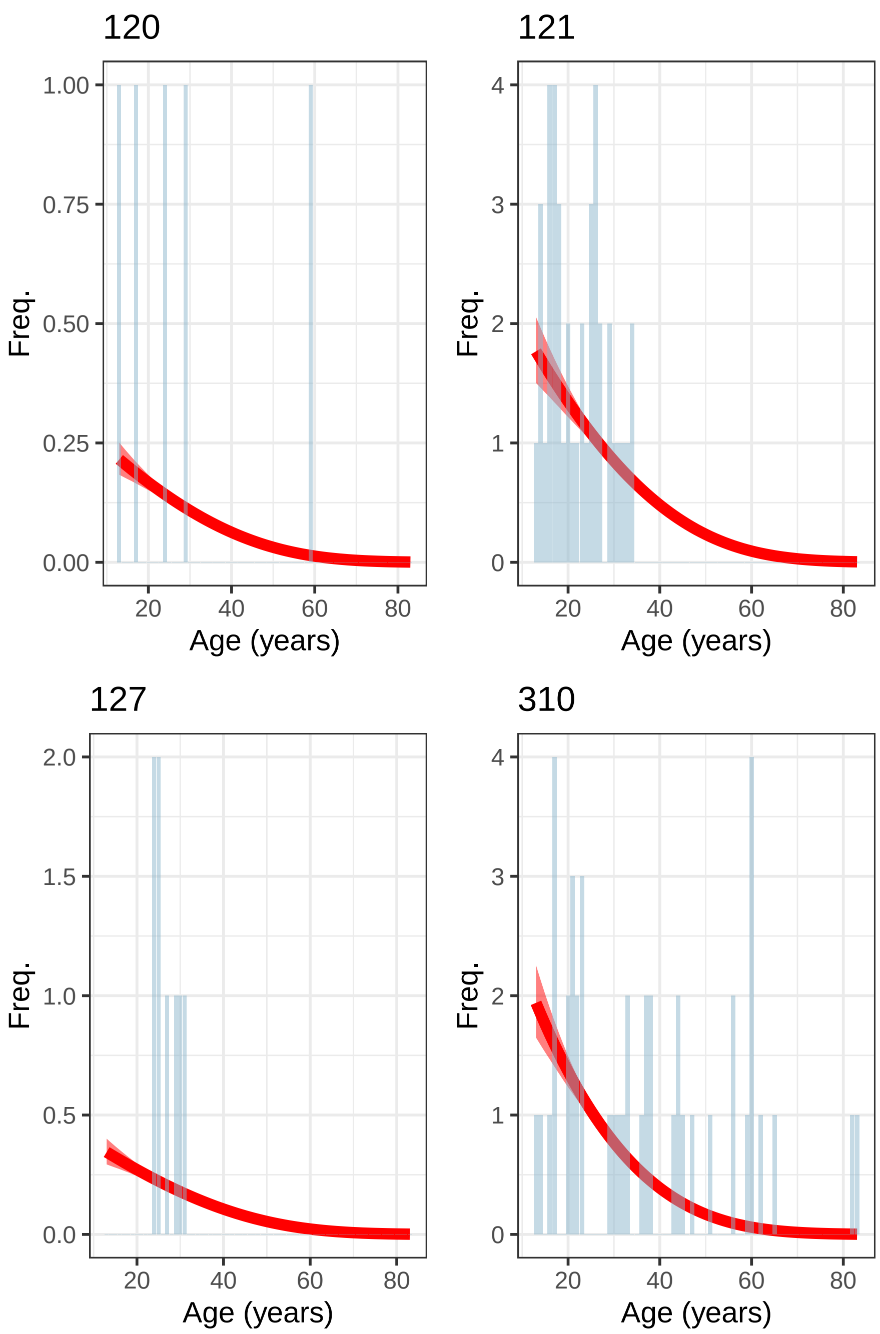

LongFinnedPilotWhale

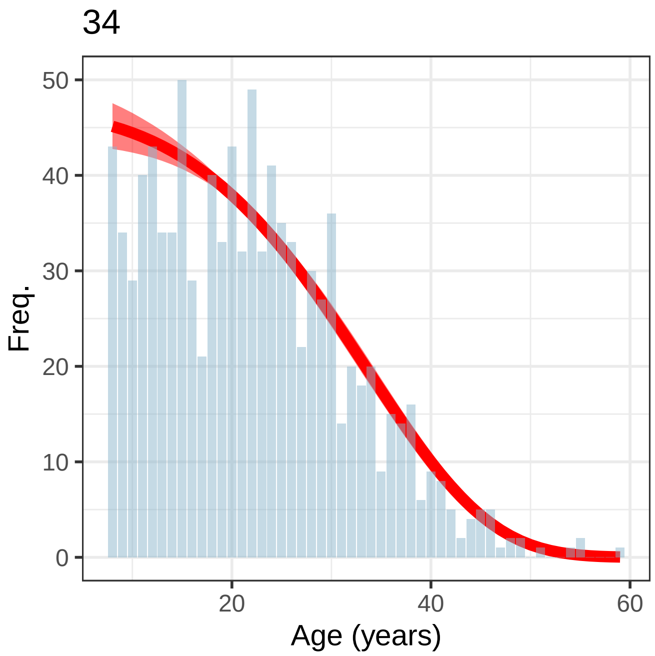

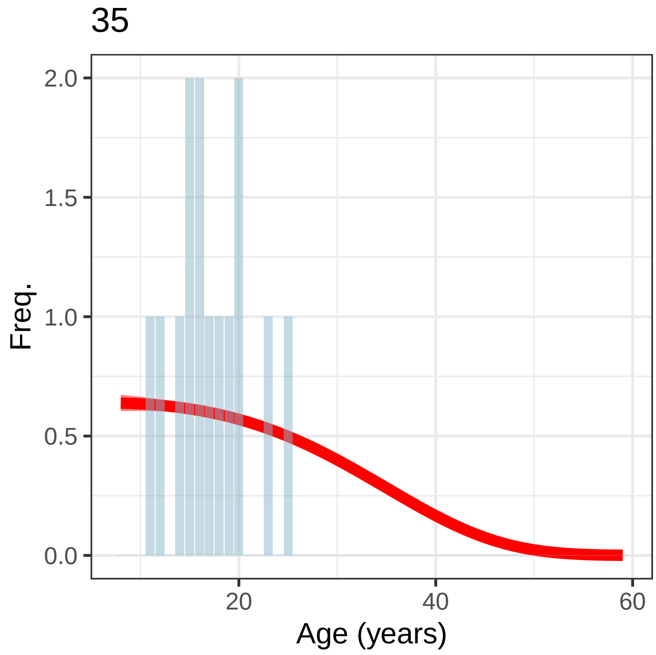

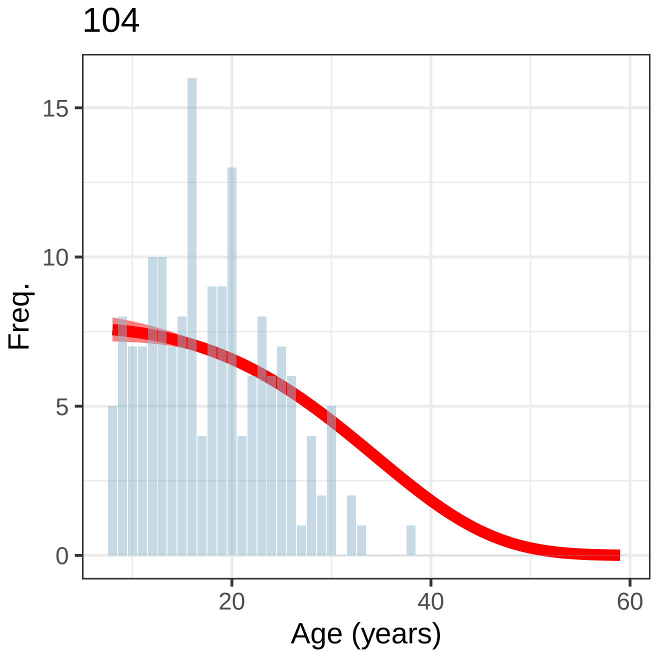

MelonHeadedWhale

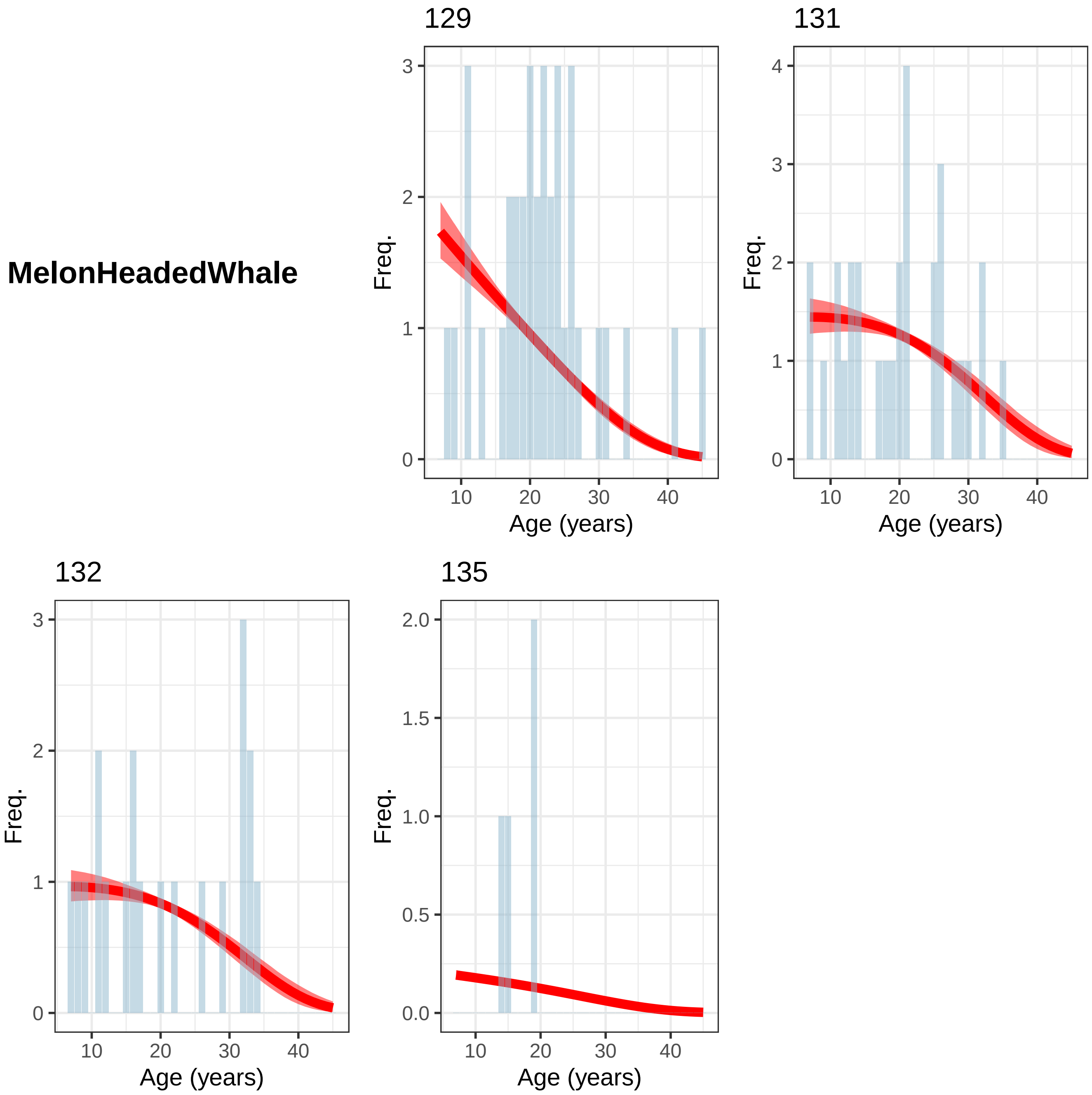

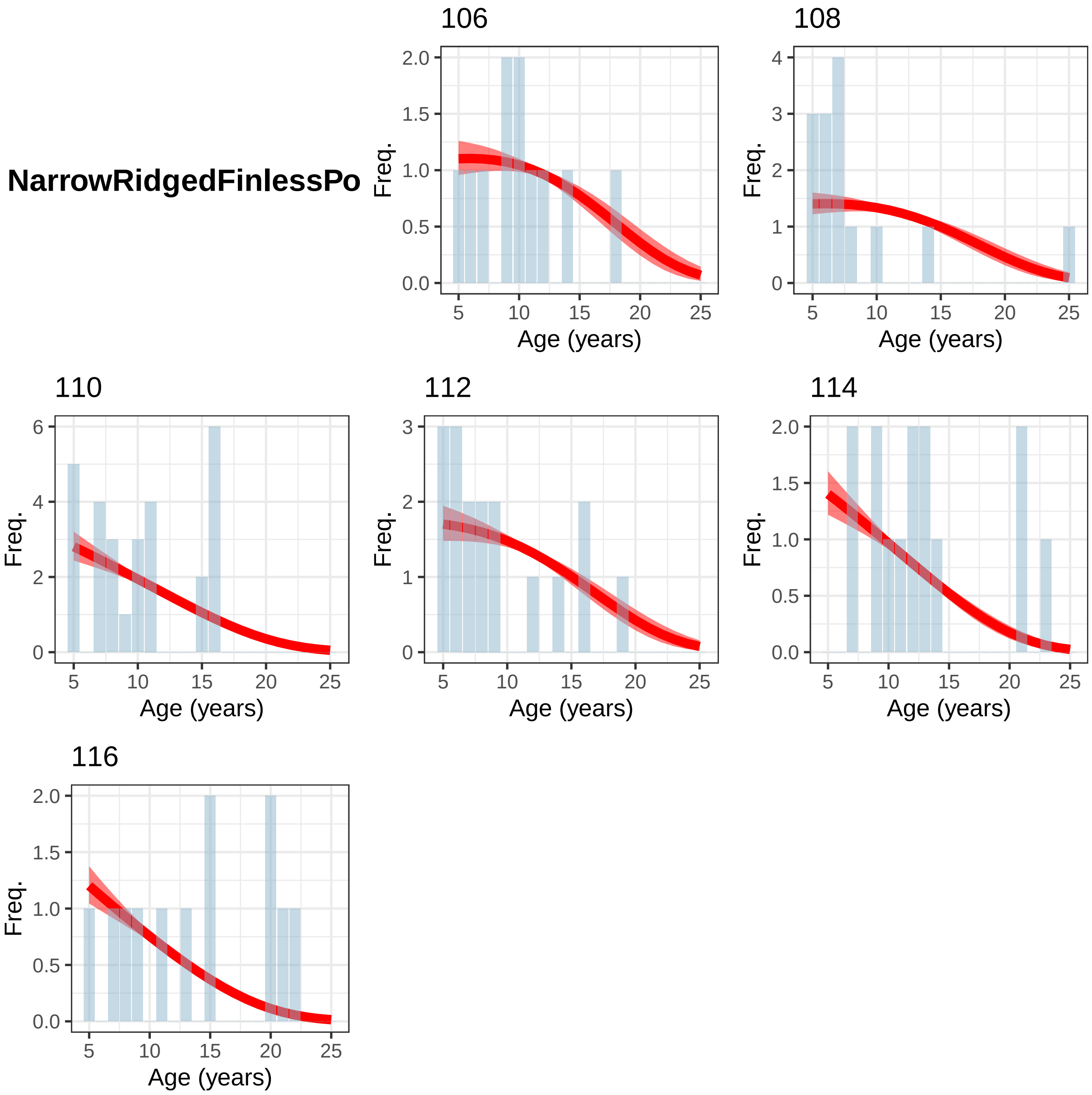
NarrowRidgedFinlessPo
106
108
110
112
114
116
Freq.
Age (years)

Narwhal

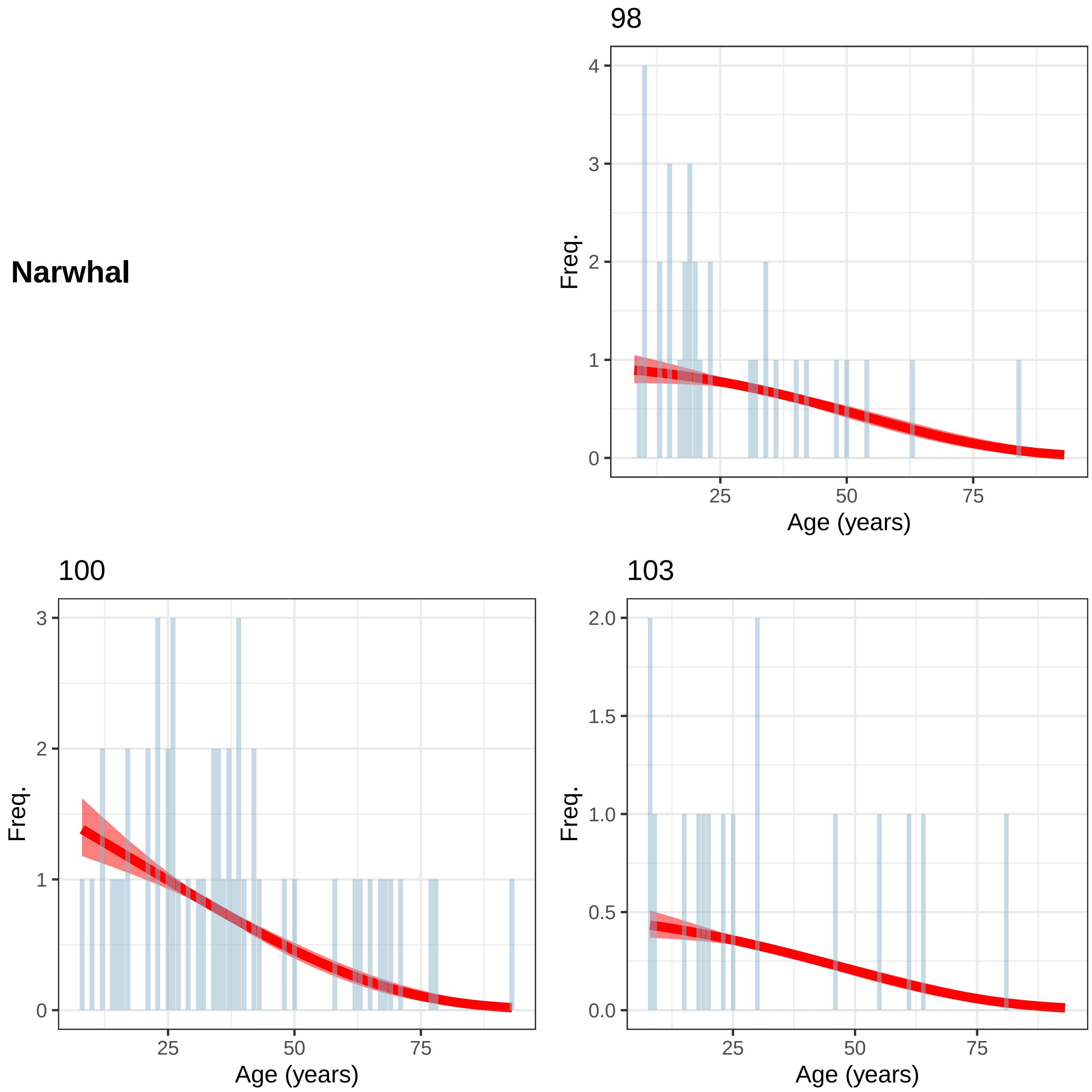

98
Freq.
0
1
2
3
4
25
50
75
Age (years)
100
Freq.
0
1
2
3
25
50
75
Age (years)
103
Freq.
0.0
0.5
1.0
1.5
2.0
25
50
75
Age (years)

NorthernBottlenoseWhale

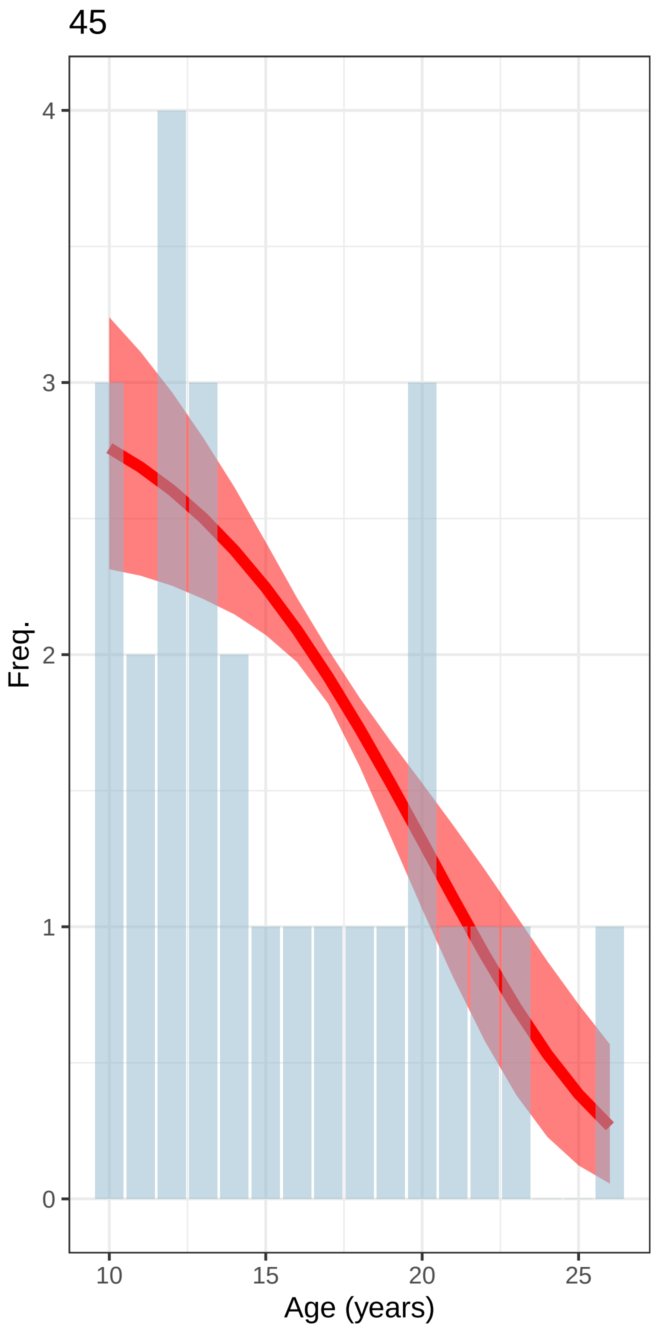

NorthernRightWhaleDolphin

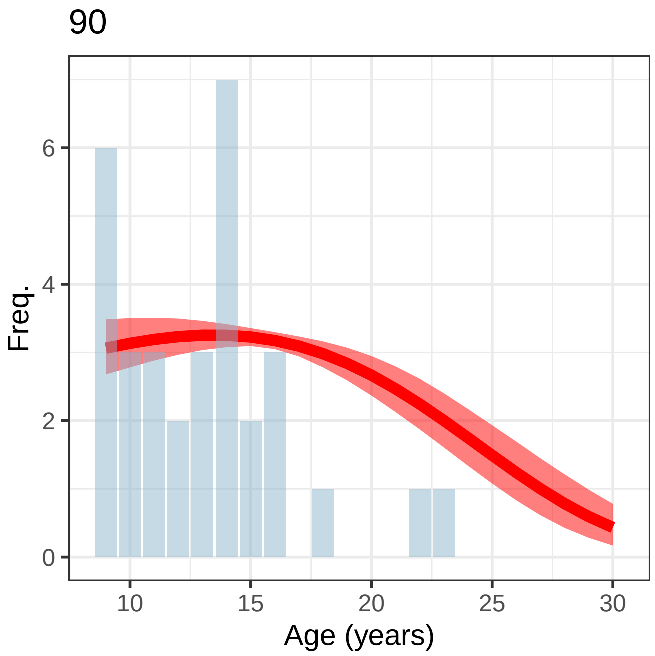

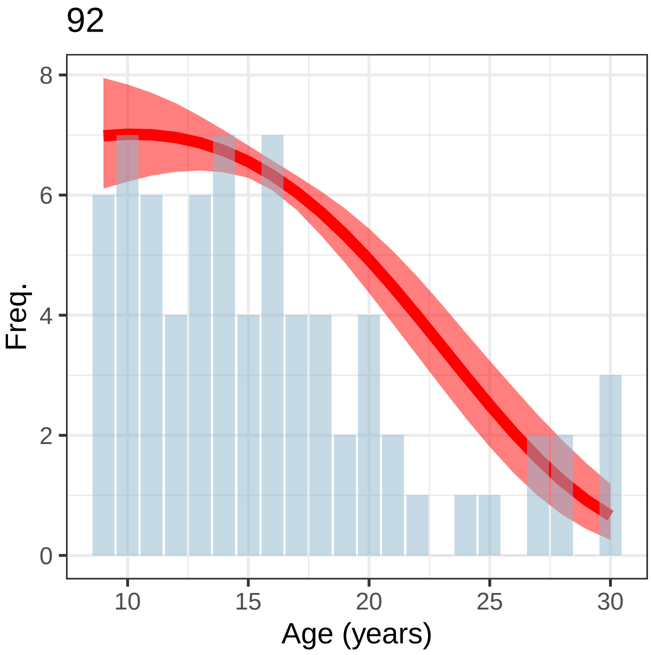

PacificWhiteSidedDolphin

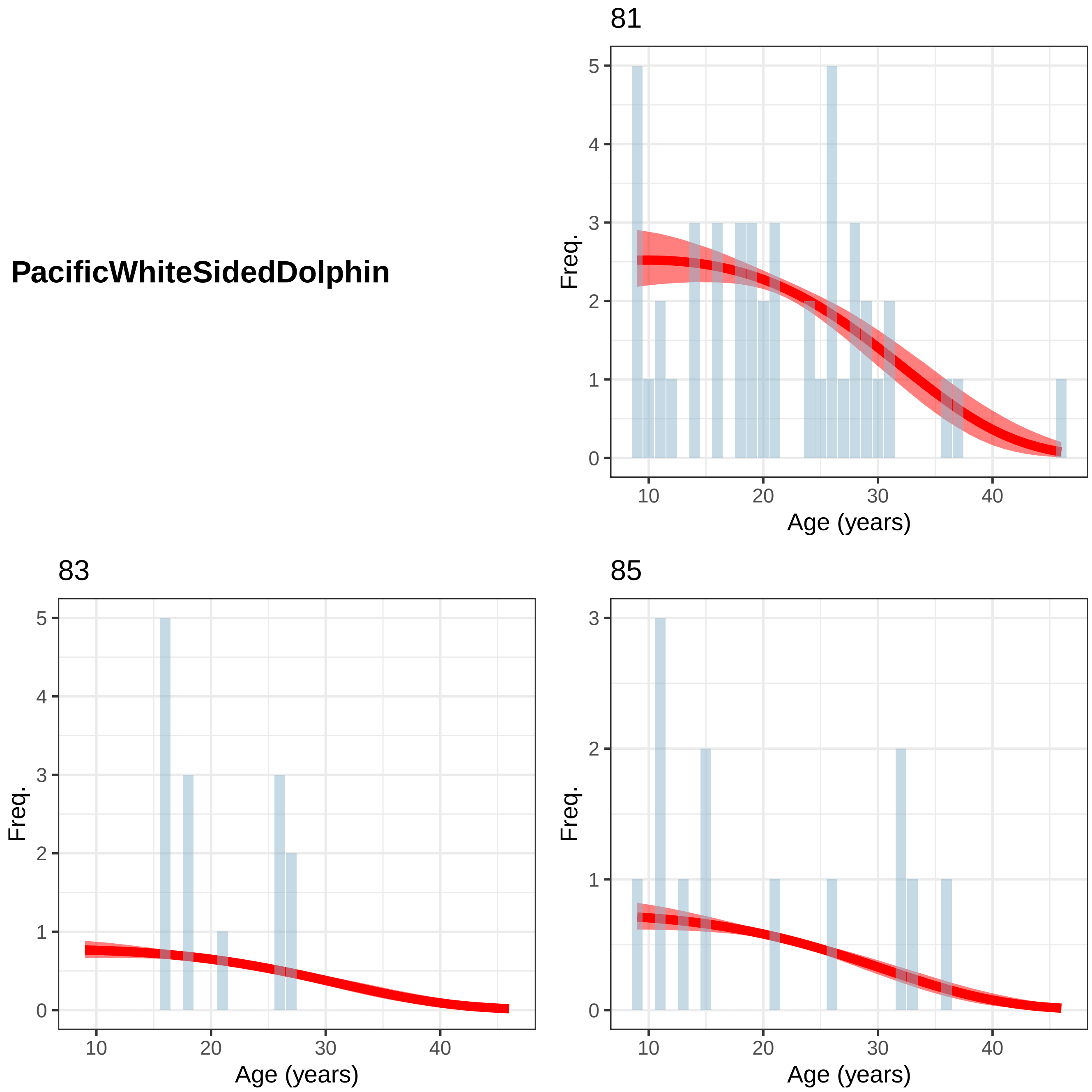

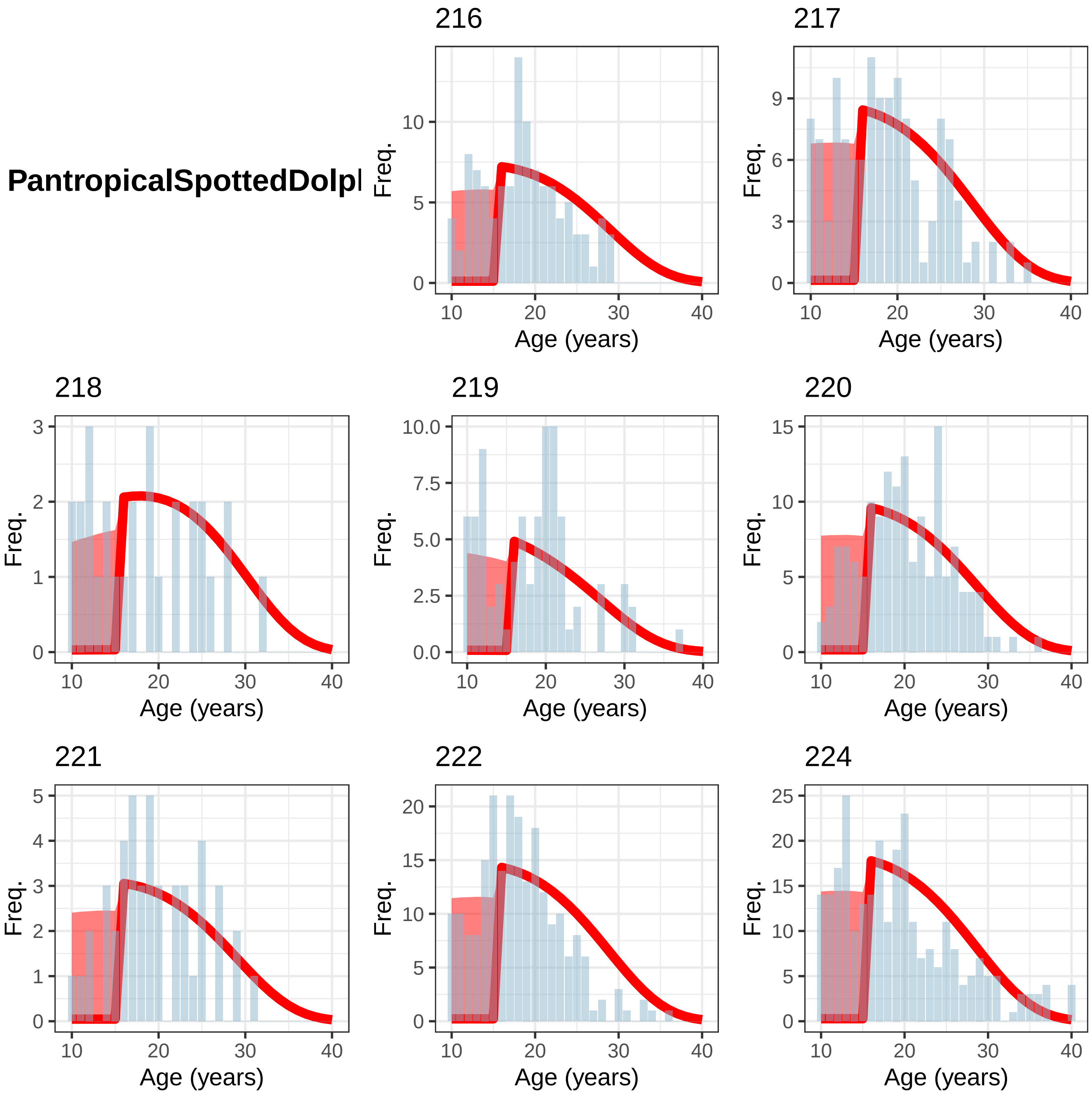

PantropicalSpottedDolpl
216
217
218
219
220
221
222
224
Freq.
Age (years)

PygmySpermWhale

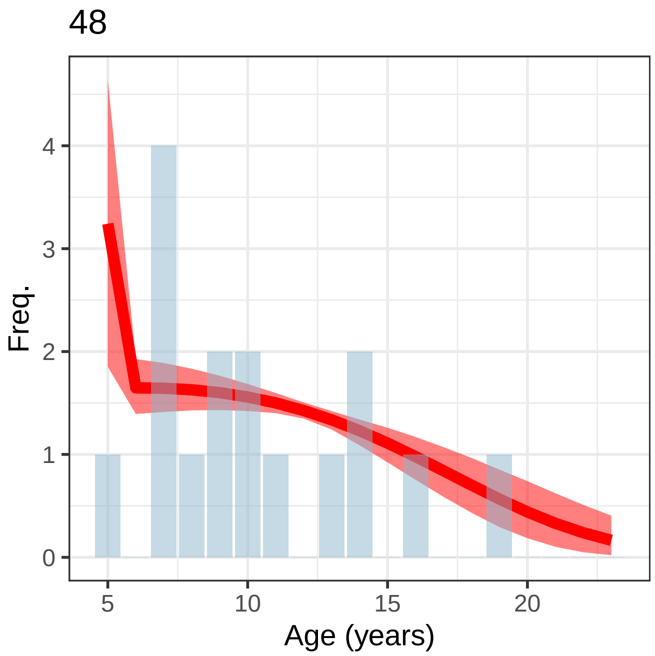

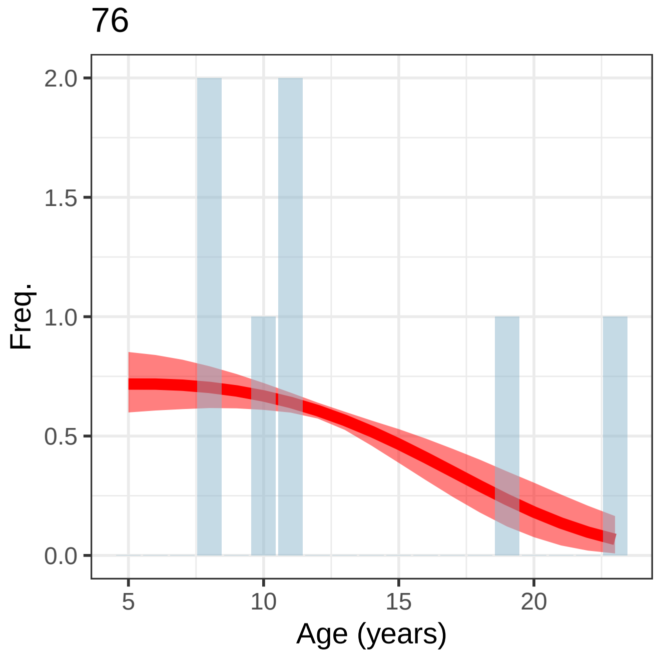

RissosDolphin

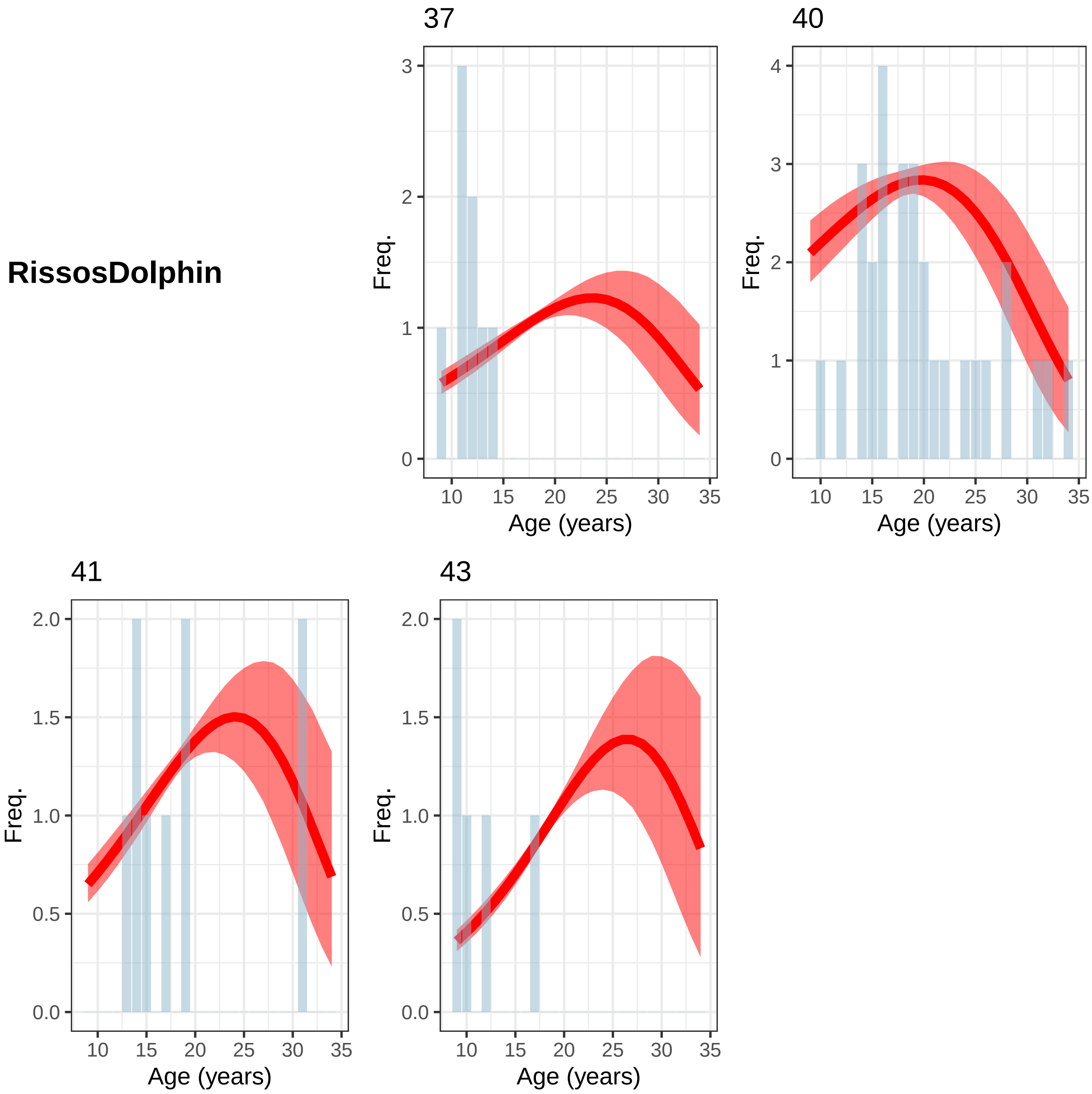

ShortFinnedPilotWhale

30

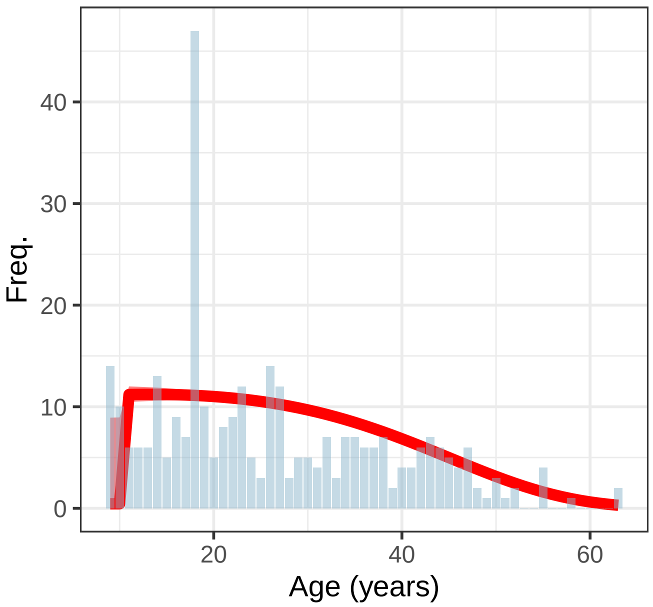

32

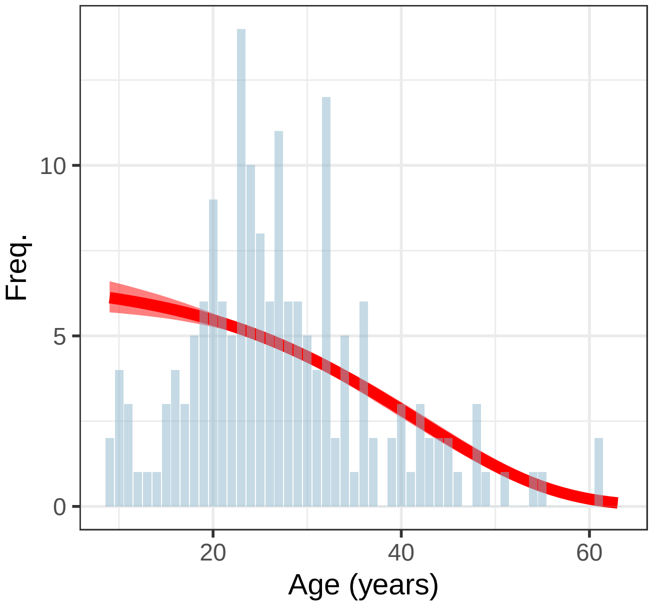

94

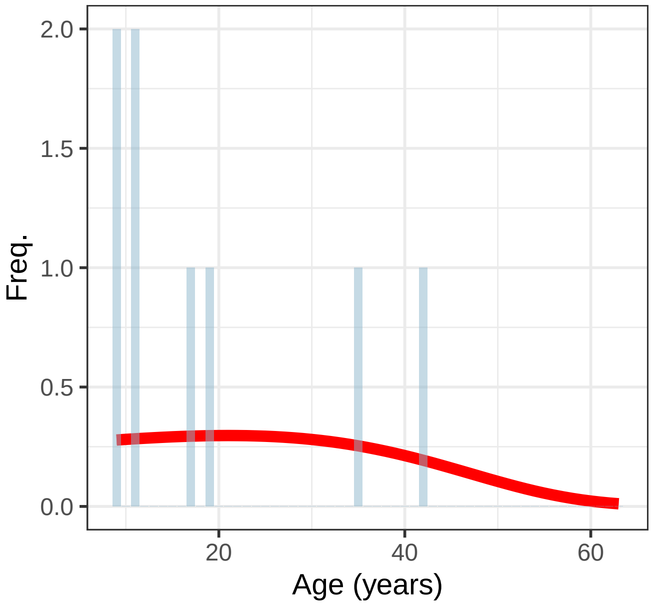

SpermWhale

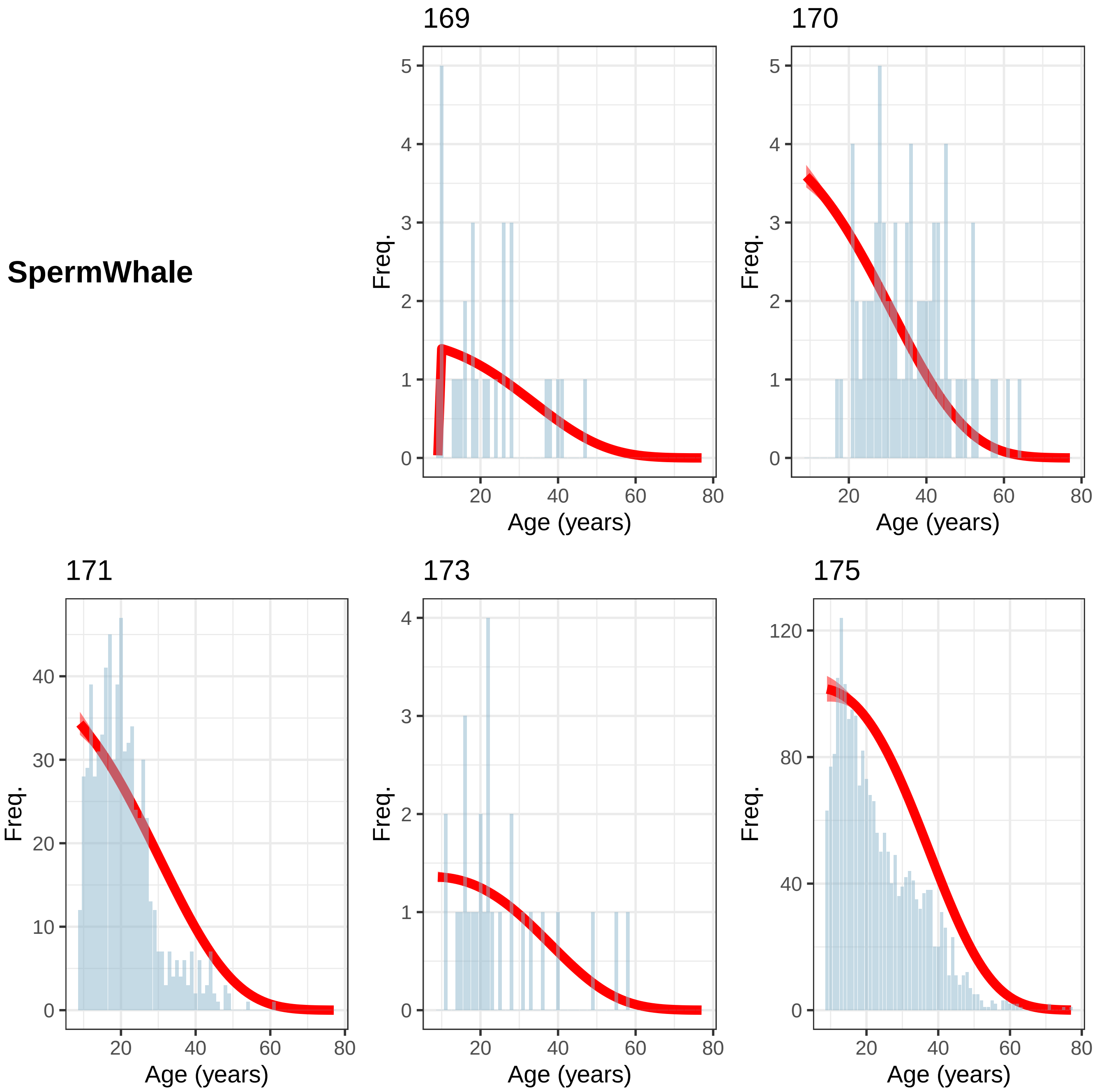

SpinnerDolphin

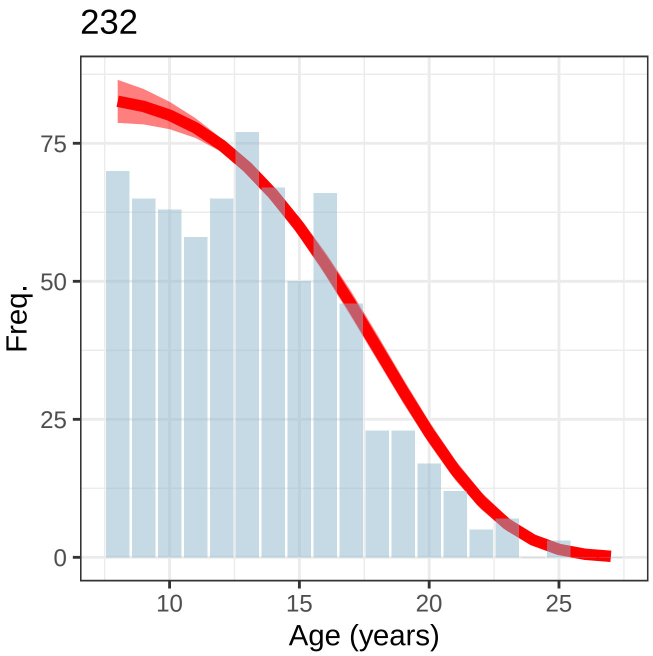

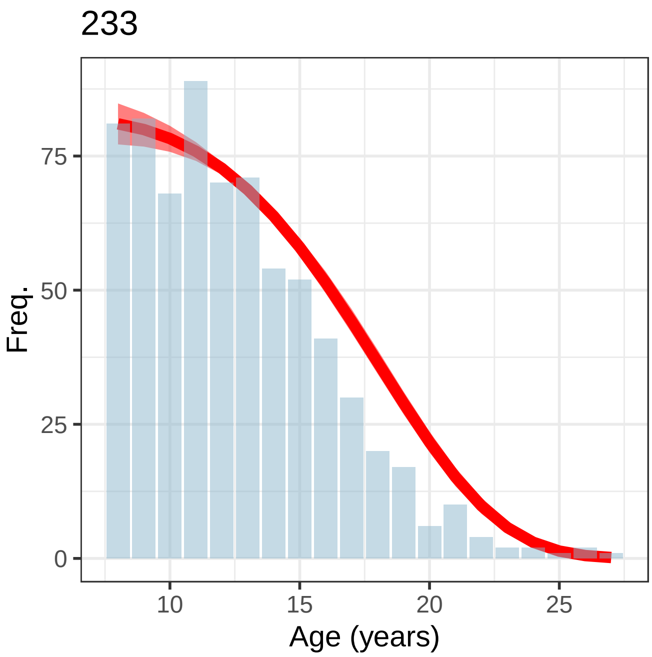

StripedDolphin

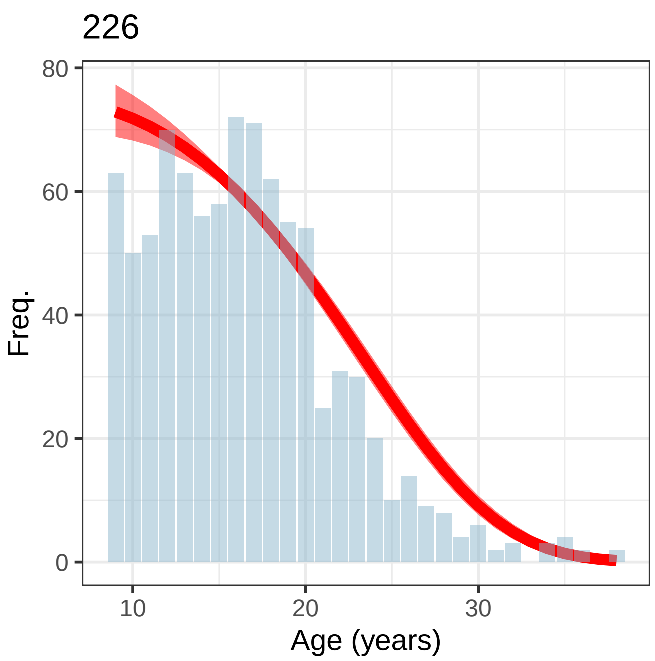

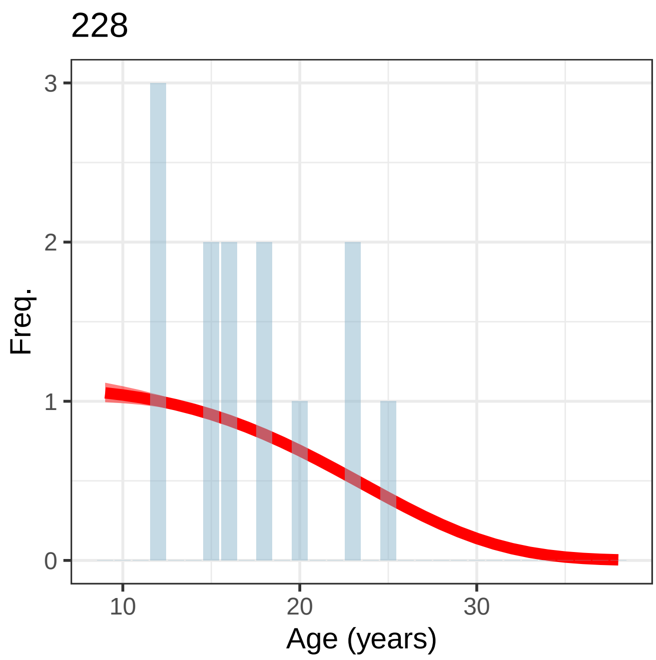

Vaquita

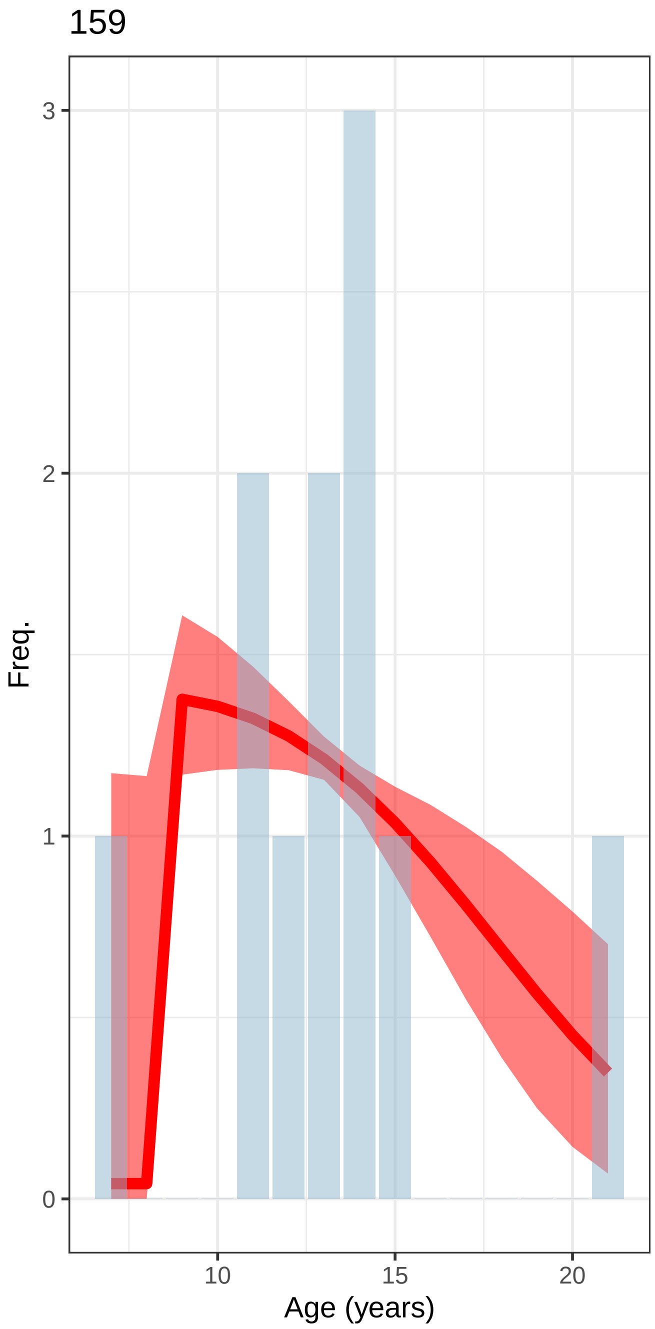

WhiteBeakedDolphin

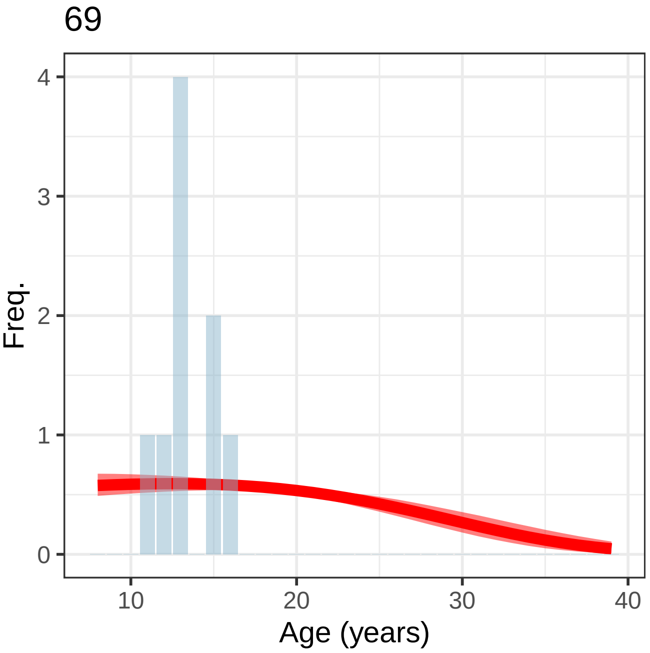

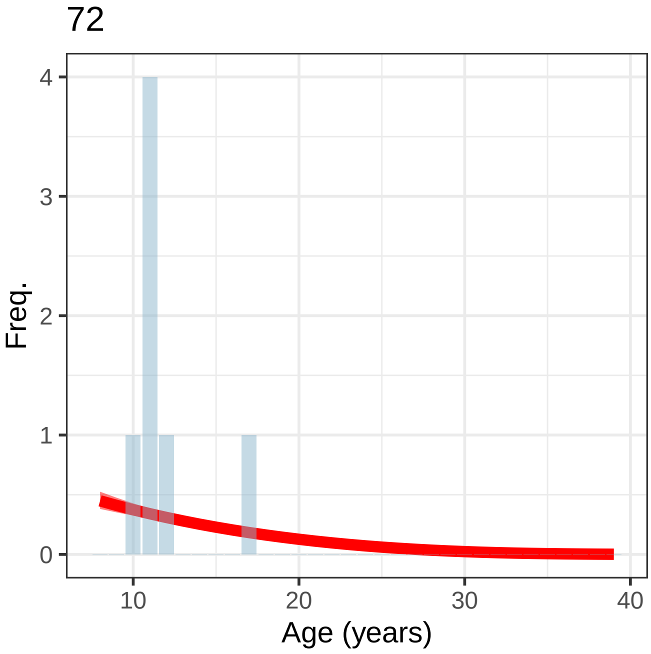

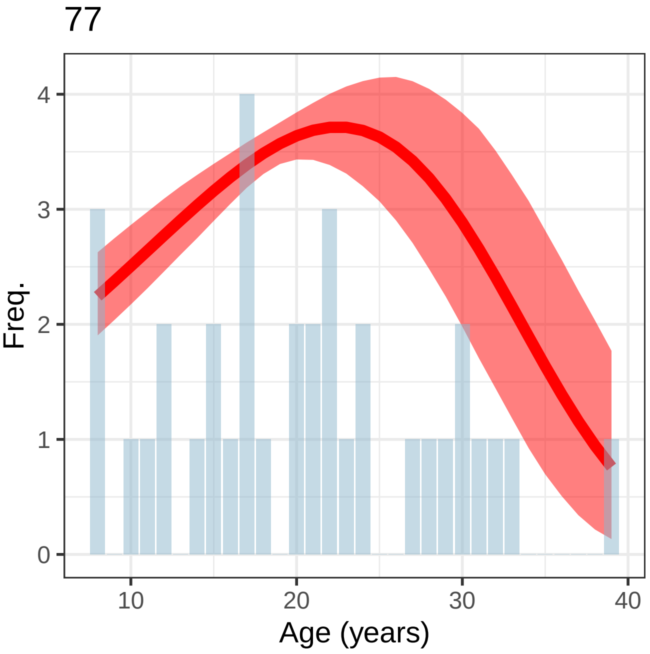

B. Fitted Corpora Models

Corpora data with fitted models (see text and equations 2 + S2).

Each plot shows the age and against corpora number for a toothed whale sample. Each dataset is derived from the literature. Points show a whale in the sample, line+ribbon show the fitted model output of ovarian activity with age (posterior mean +/- 95% cred. int.). Where multiple datasets are available from the same species each dataset differs in the initial rate of corpora deposition (model α) but share a rate of corpora decline (model β). Plot titles show species and #NUMBER shows dataset number from the marine life history database (github.com/samellisq/marinelifehistdata).

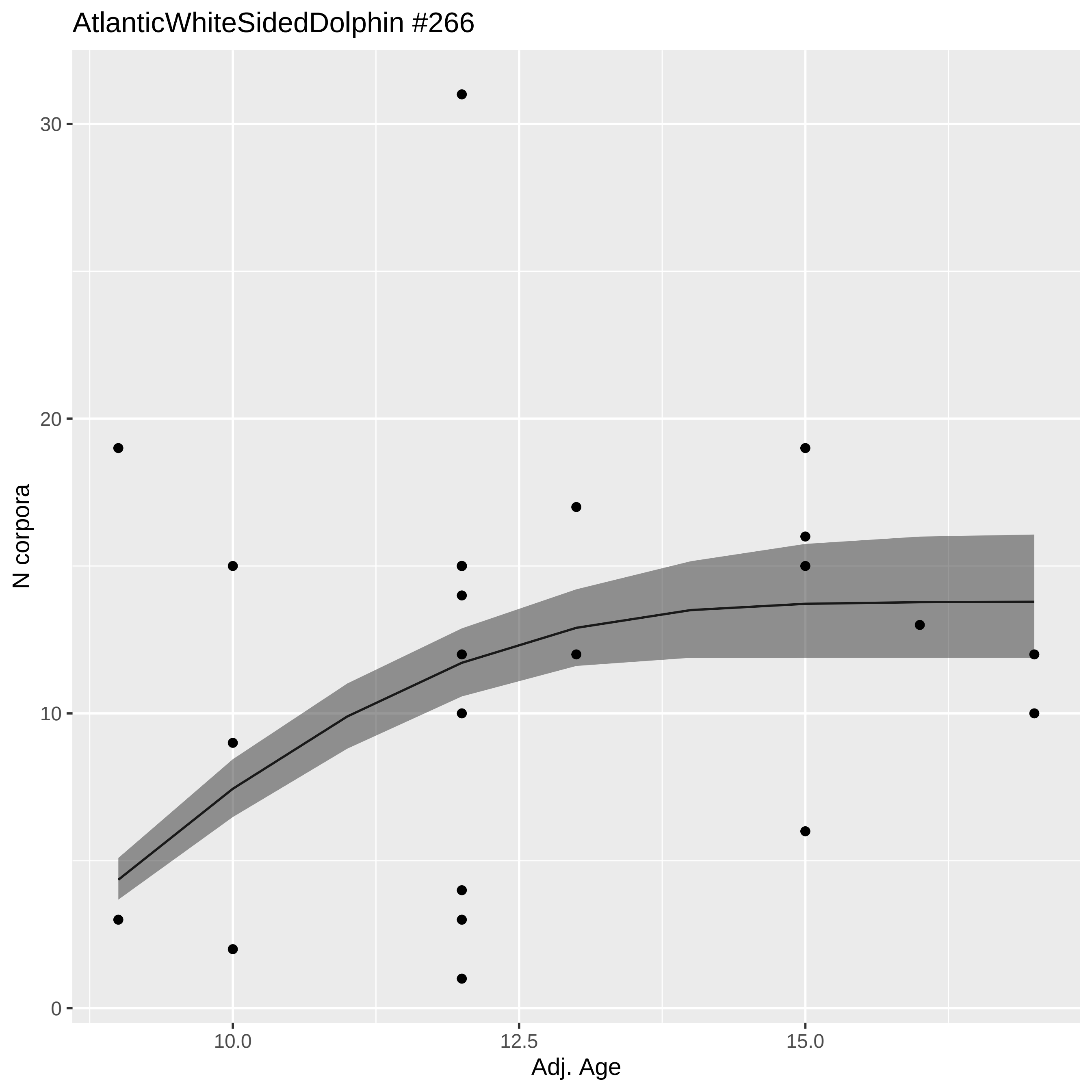

AtlanticWhiteSidedDolphin #266
30
20
10
0
10.0
12.5
15.0
Adj. Age
N corpora

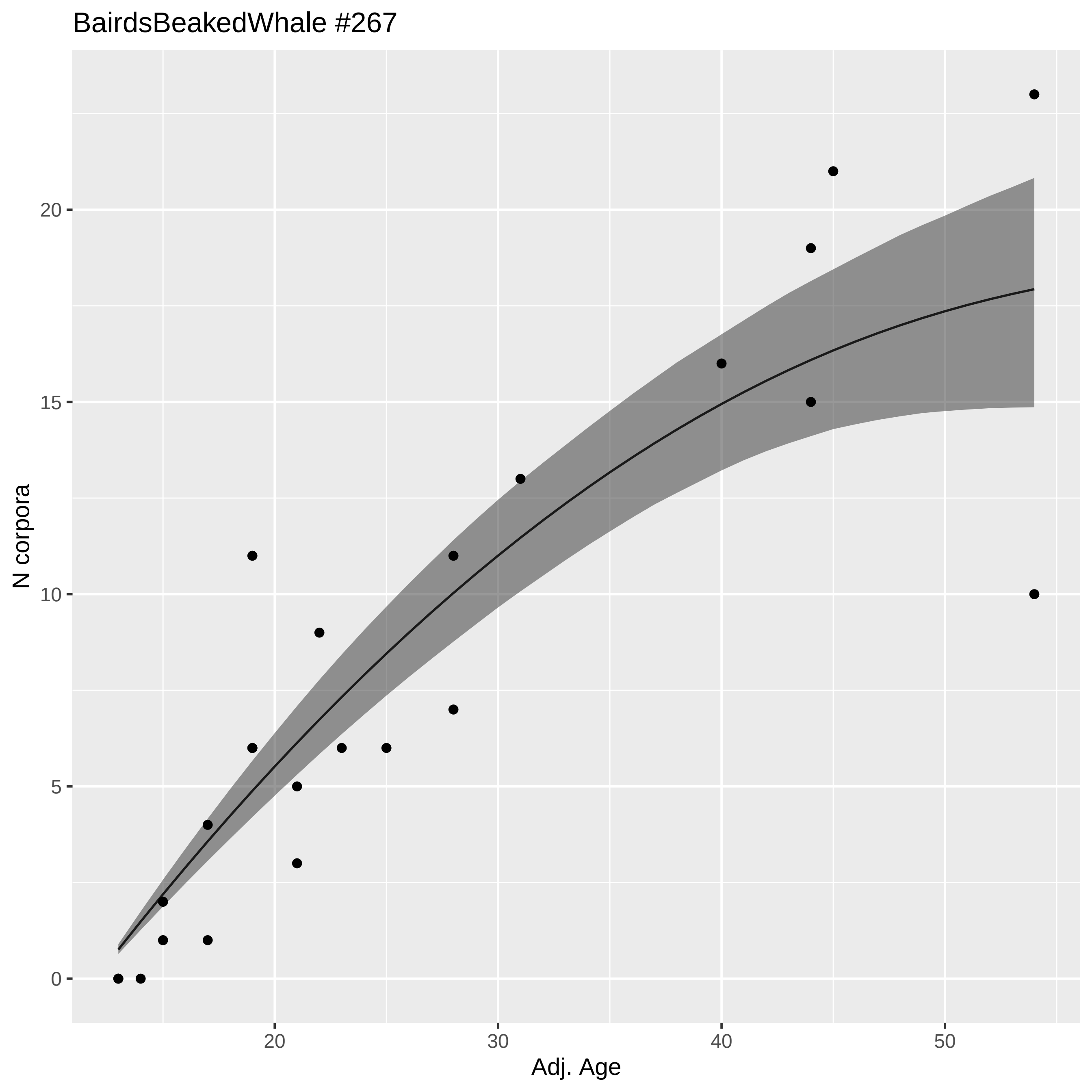
BairdsBeakedWhale #267
N corpora
0
5
10
15
20
20
30
40
50
Adj. Age

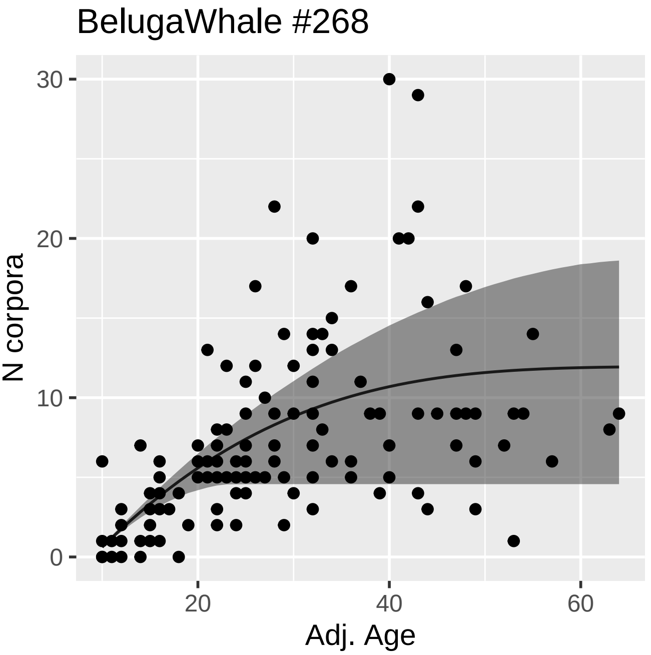
BelugaWhale #268
N corpora
Adj. Age
0
10
20
30
20
40
60

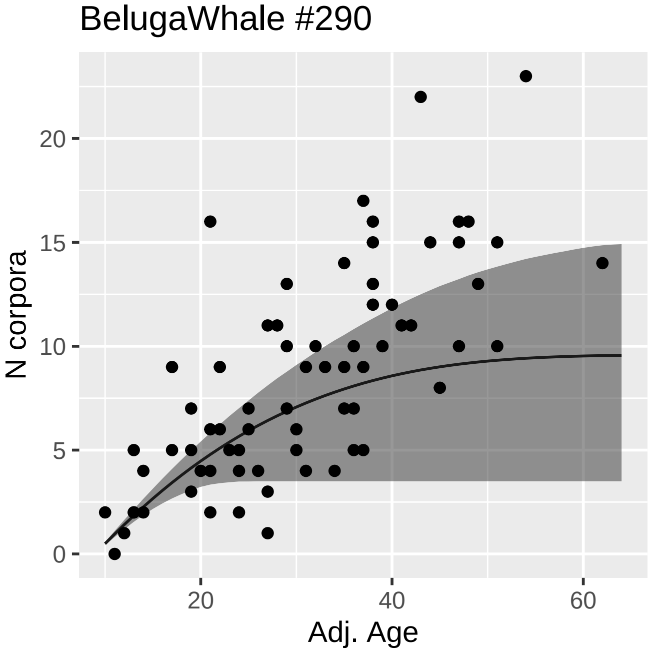
BelugaWhale #290
N corpora
Adj. Age
0
5
10
15
20
20
40
60

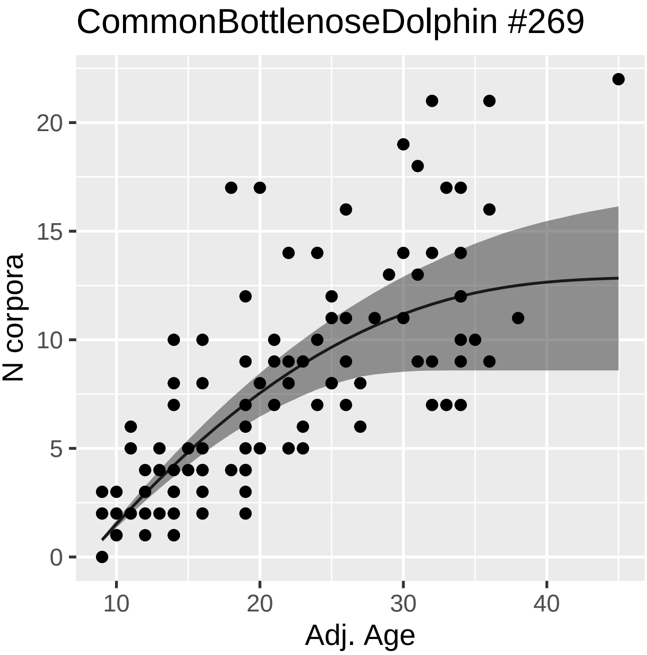
CommonBottlenoseDolphin #269
N corpora
0
5
10
15
20
10
20
30
40
Adj. Age

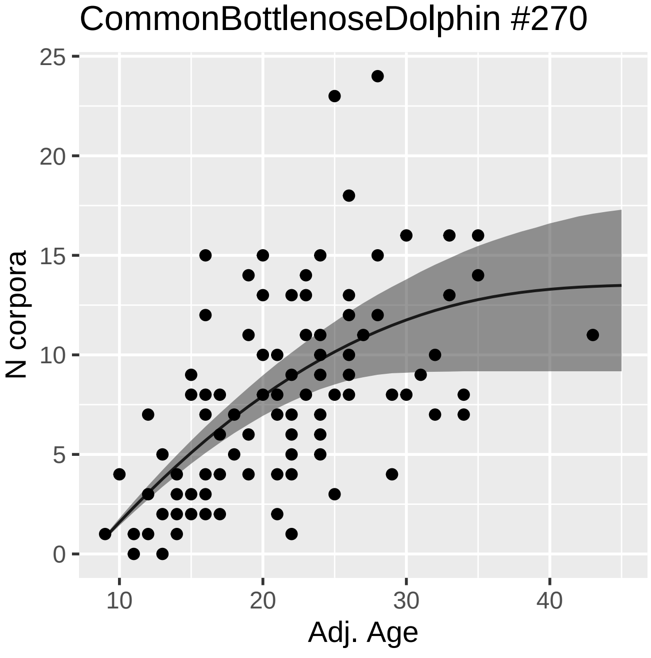
CommonBottlenoseDolphin #270
N corpora
0
5
10
15
20
25
10
20
30
40
Adj. Age

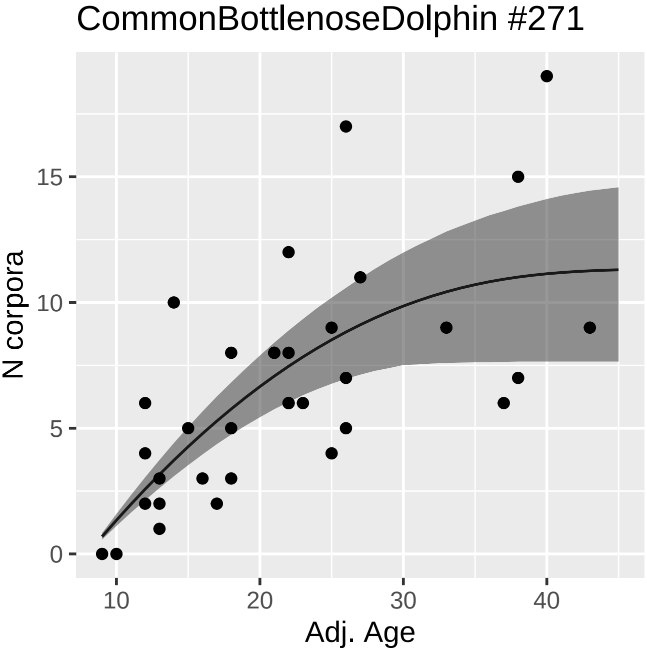
CommonBottlenoseDolphin #271
N corpora
0
5
10
15
10
20
30
40
Adj. Age

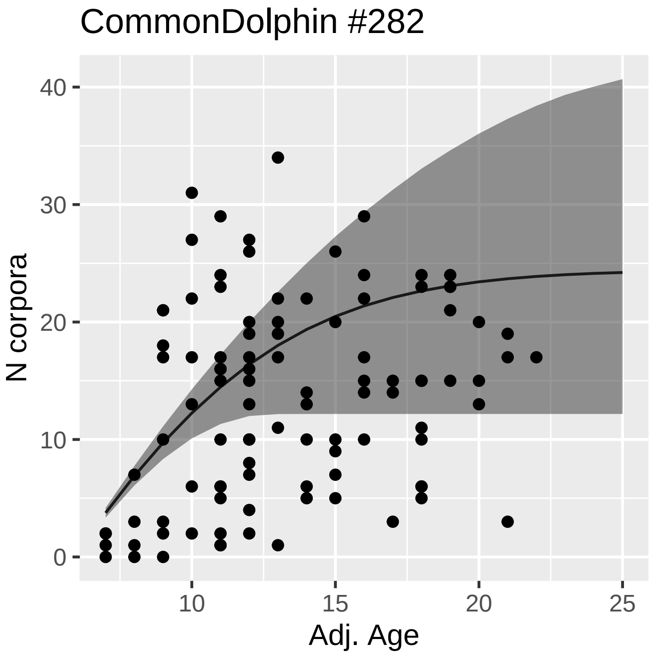
CommonDolphin #282
N corpora
Adj. Age
0
10
20
30
40
10
15
20
25

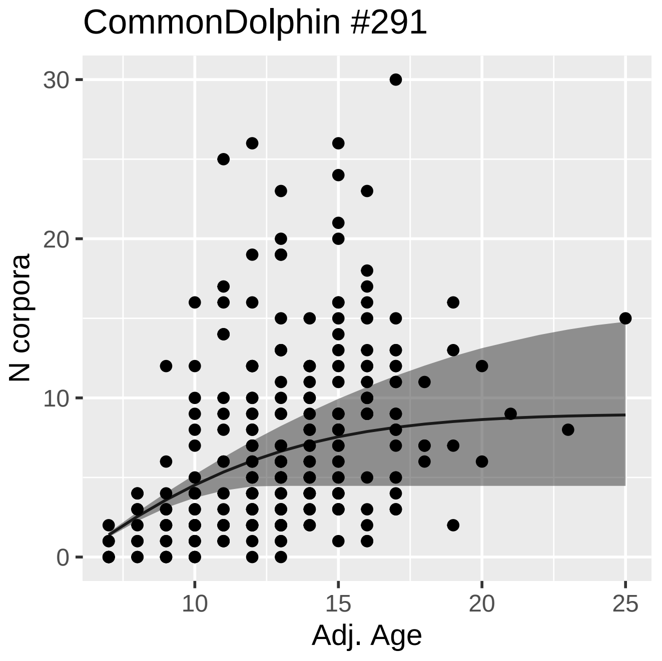
CommonDolphin #291
N corpora
Adj. Age
0
10
20
30
10
15
20
25

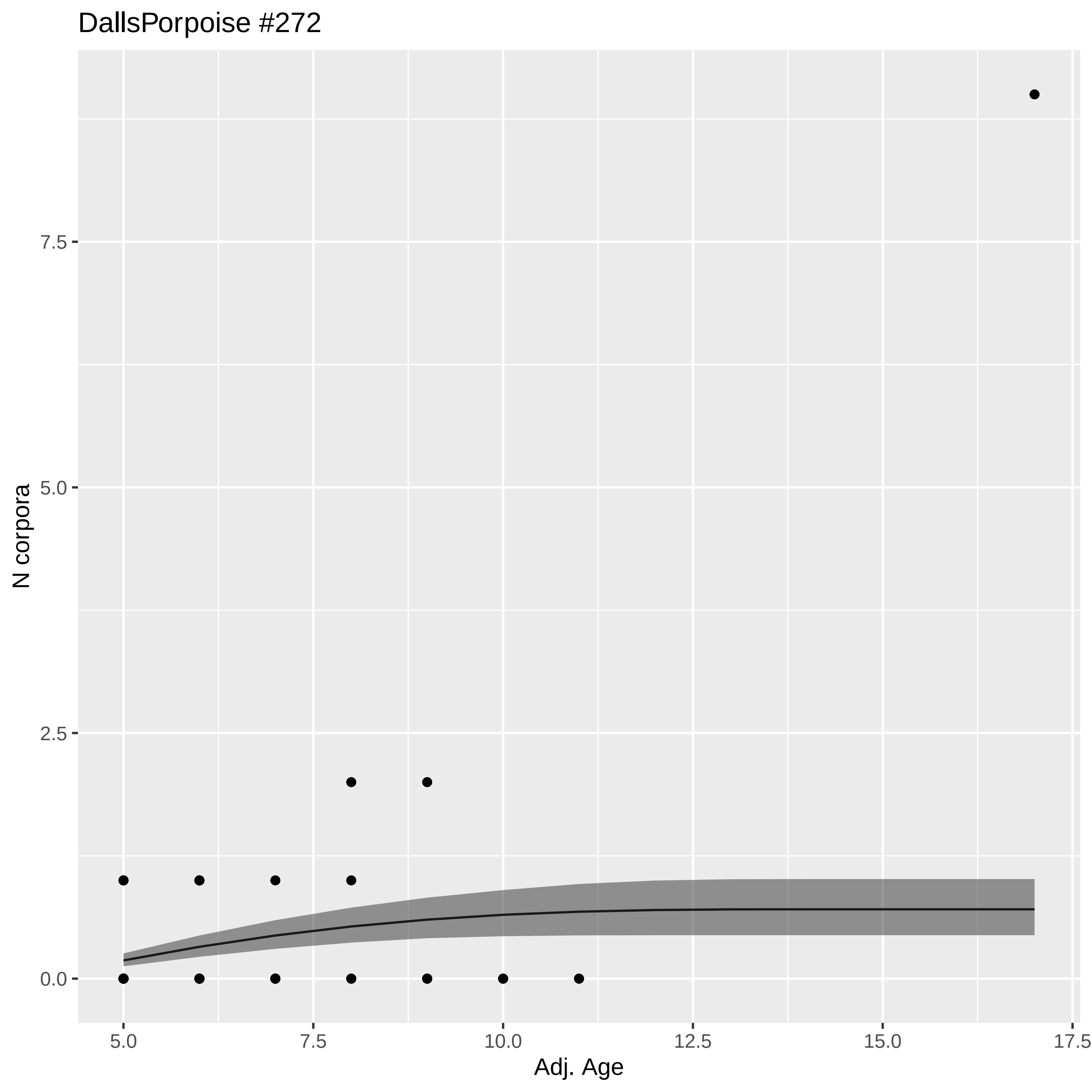
DallsPorpoise #272
N corpora
7.5
5.0
2.5
0.0
5.0
7.5
10.0
12.5
15.0
17.5
Adj. Age

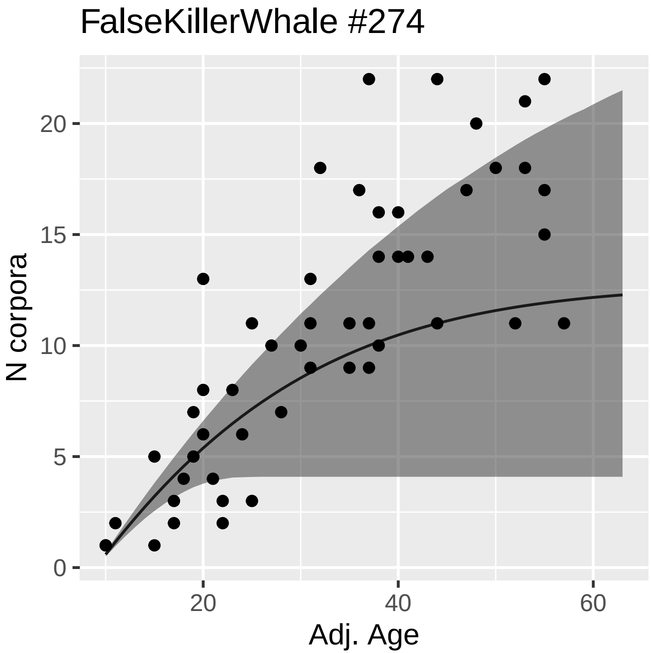
FalseKillerWhale #274
N corpora
Adj. Age
0
5
10
15
20
20
40
60

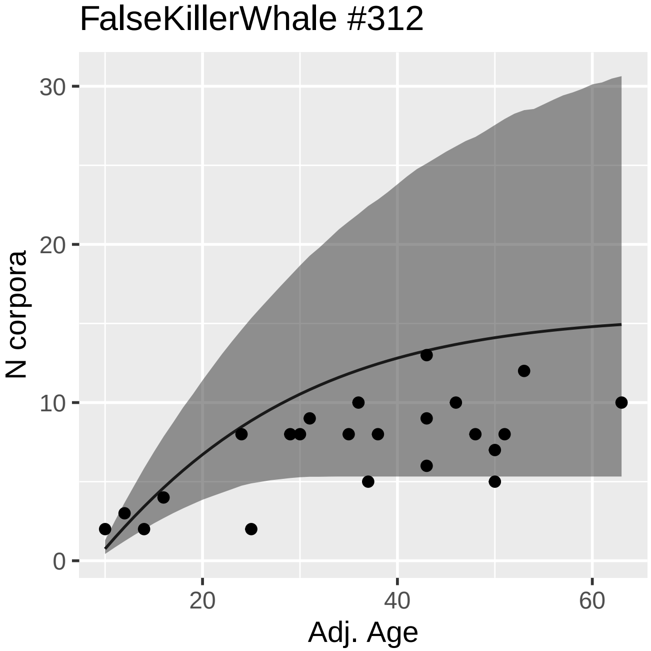
FalseKillerWhale #312
N corpora
Adj. Age
0
10
20
30
20
40
60

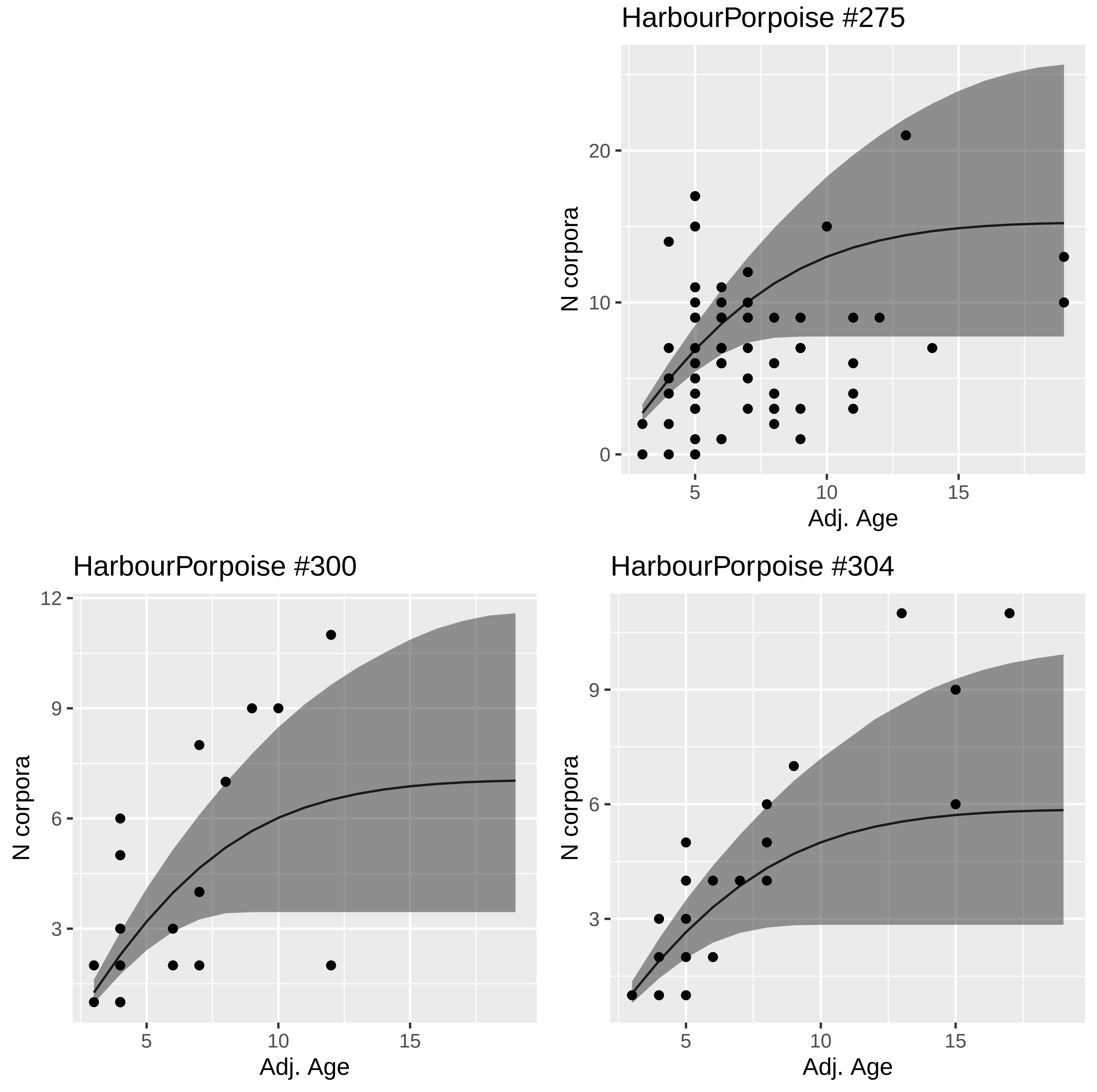

HarbourPorpoise #275
N corpora
Adj. Age
HarbourPorpoise #300
N corpora
Adj. Age
HarbourPorpoise #304
N corpora
Adj. Age

IndoPacificBottlenoseDolphin #302

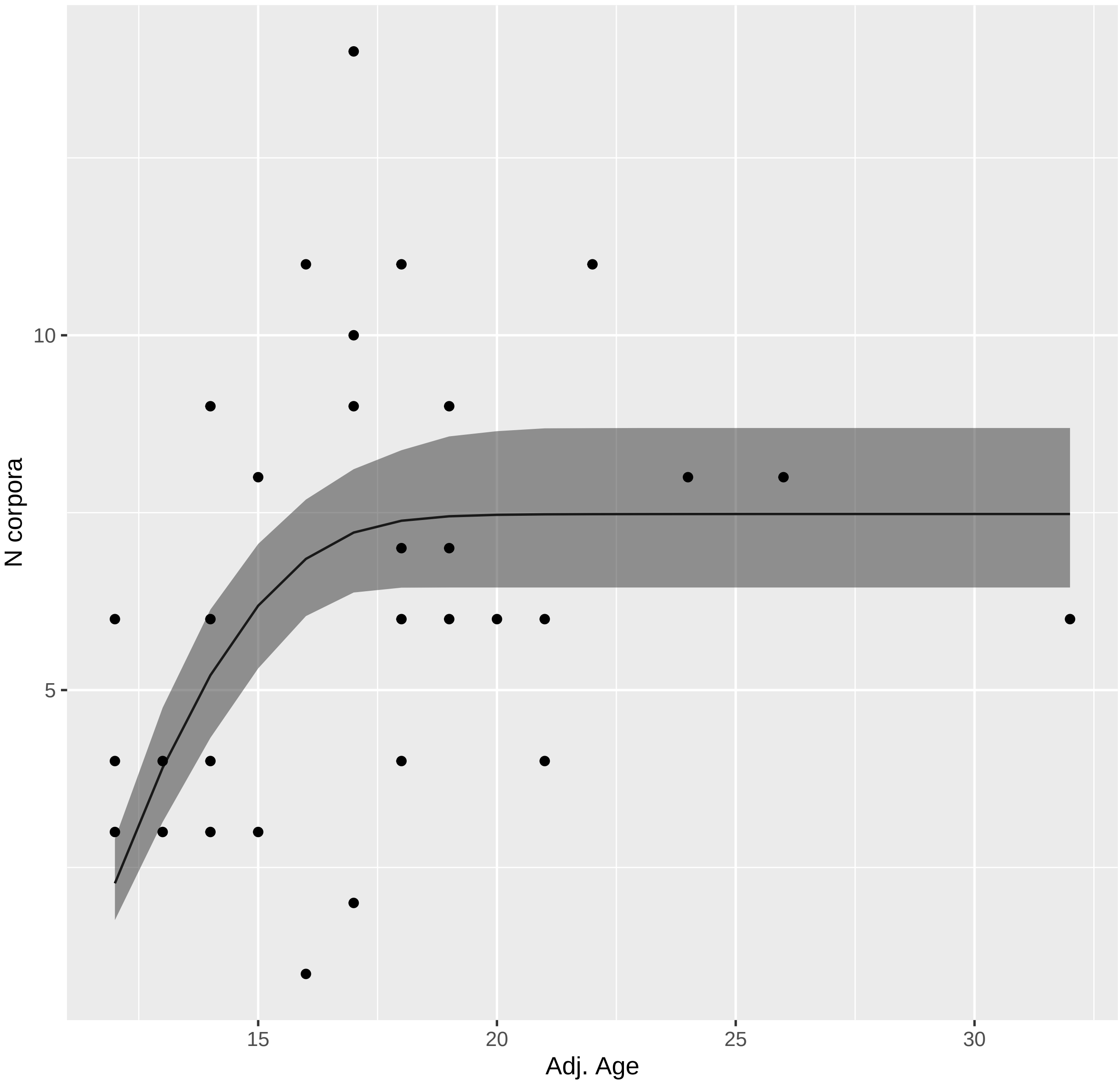

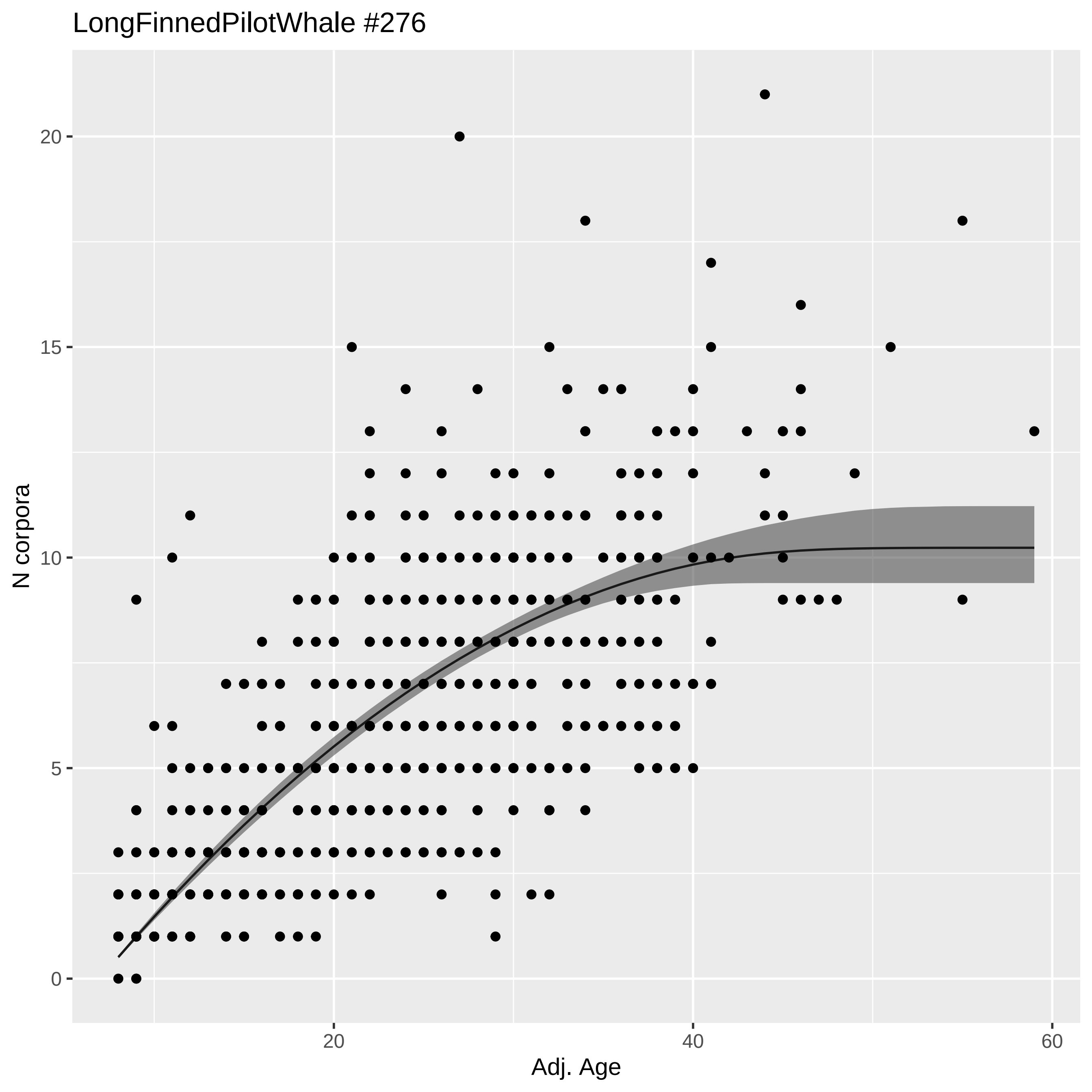
LongFinnedPilotWhale #276
N corpora
Adj. Age
0
5
10
15
20
20
40
60

MelonHeadedWhale #278

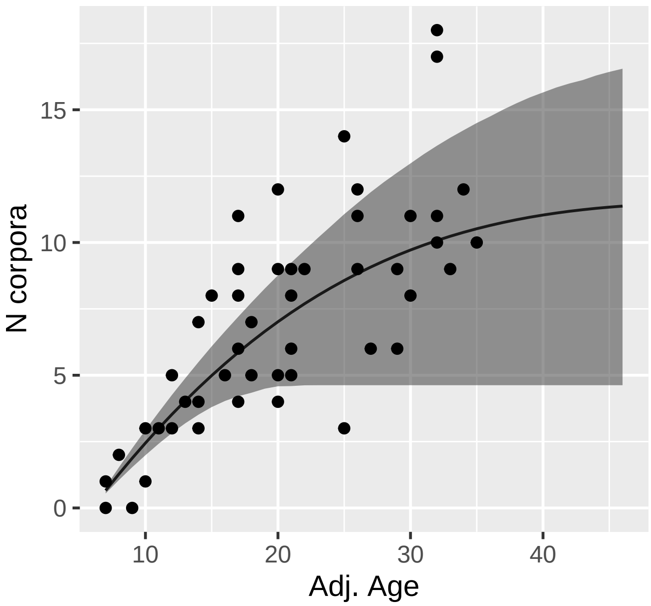

MelonHeadedWhale #299

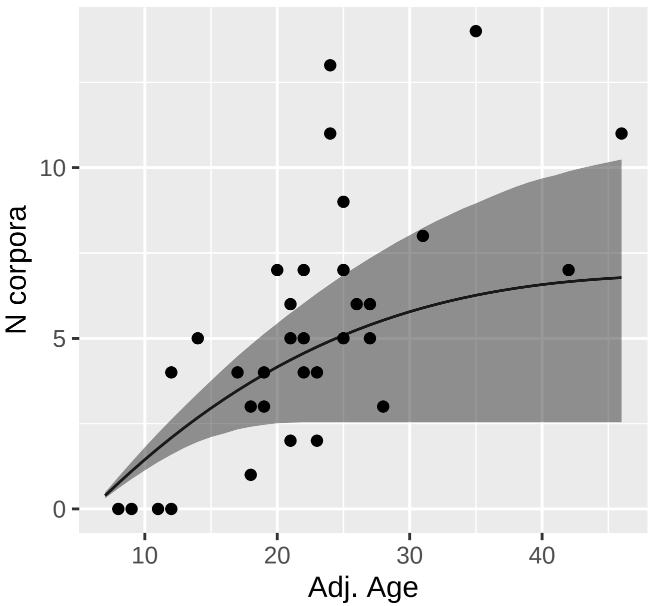

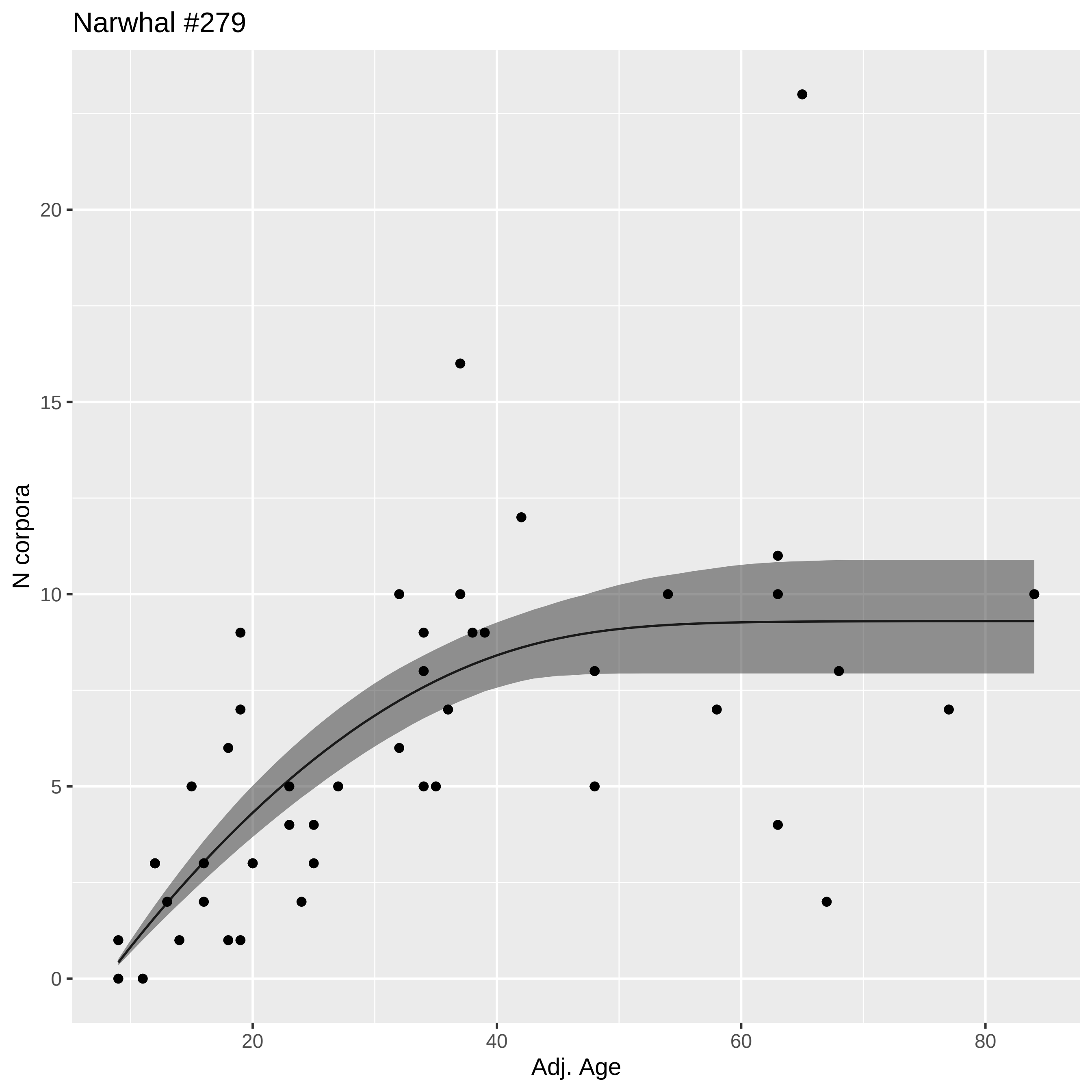
Narwhal #279
N corpora
Adj. Age
0
5
10
15
20
20
40
60
80

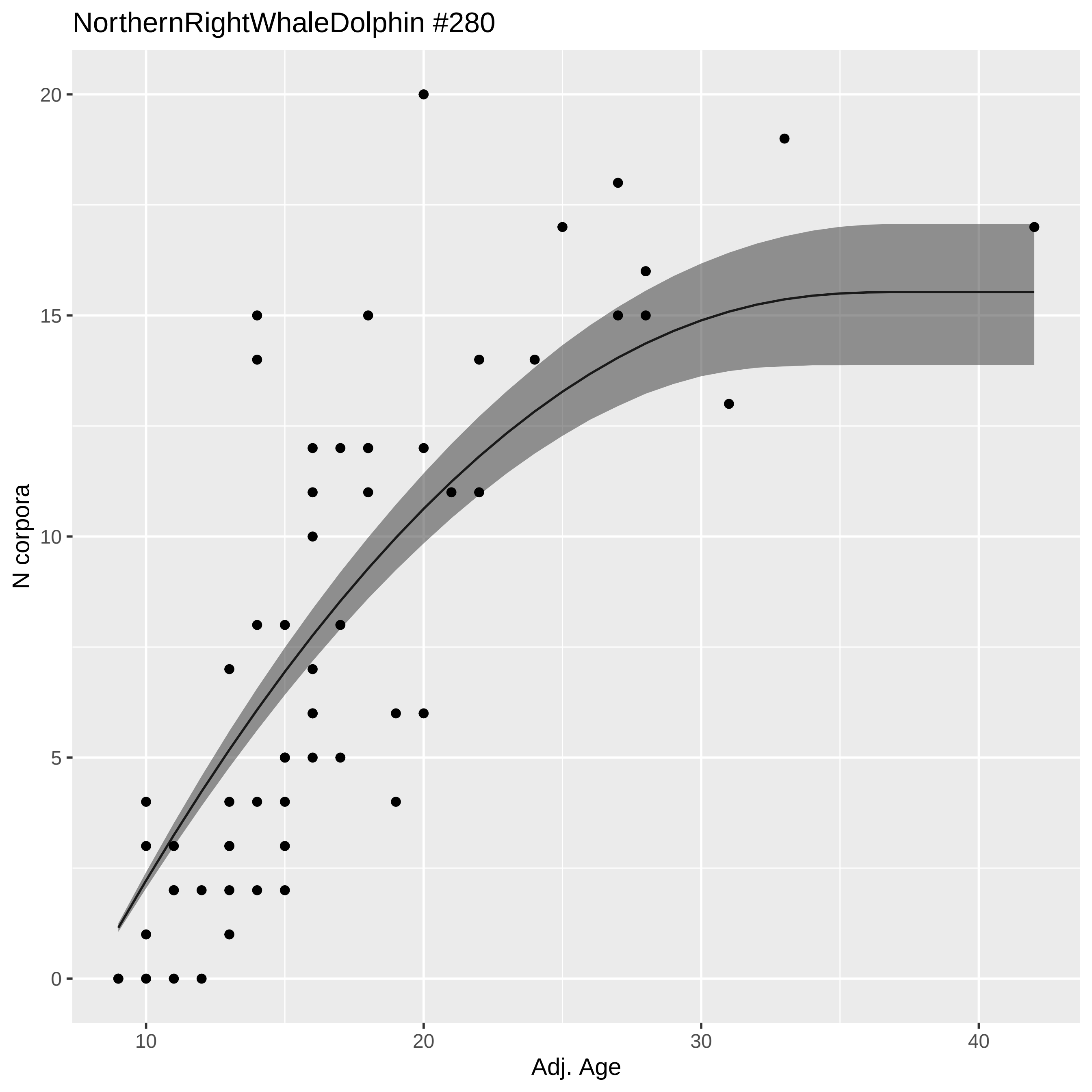
NorthernRightWhaleDolphin #280
N corpora
Adj. Age
0
5
10
15
20
10
20
30
40

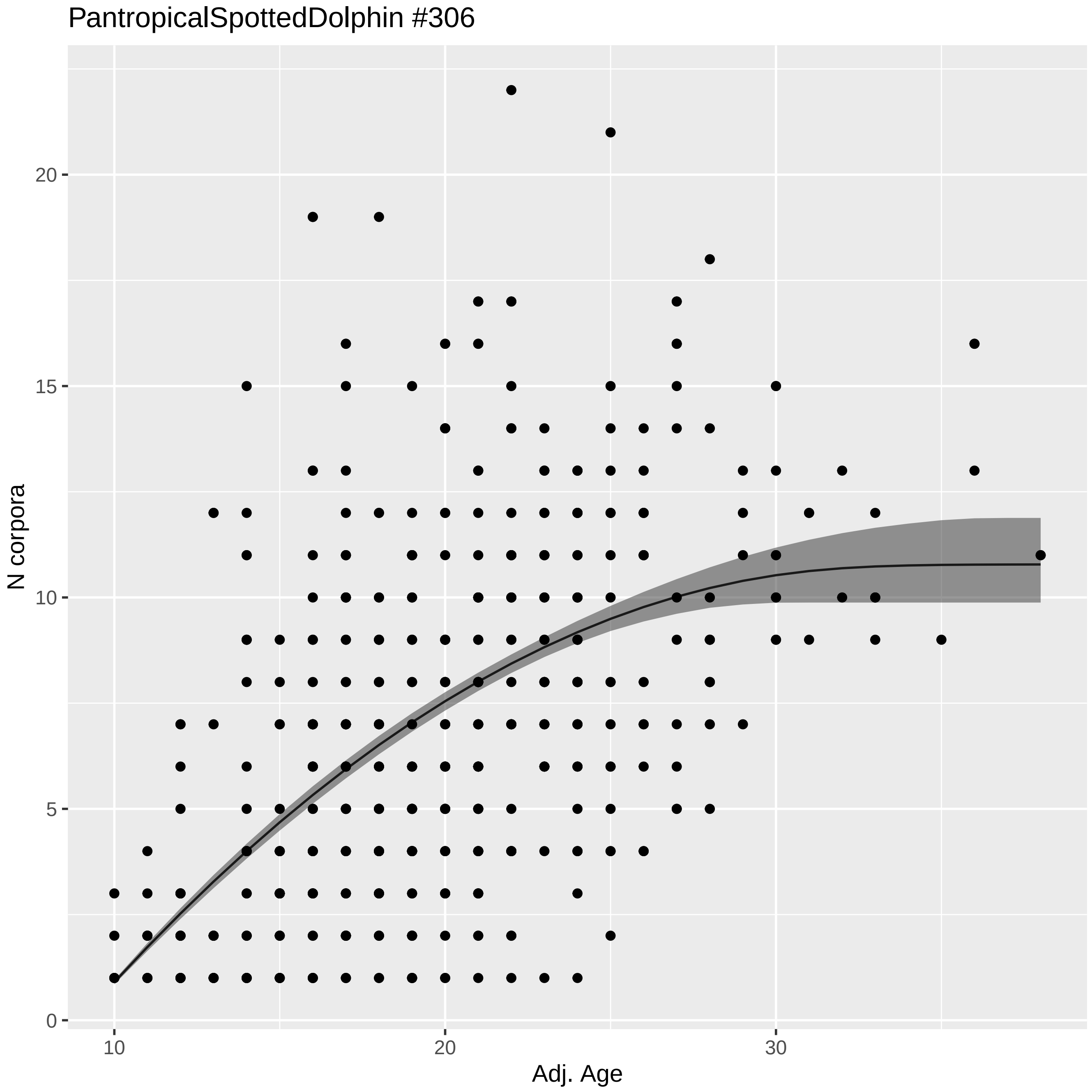
PantropicalSpottedDolphin #306
N corpora
0
5
10
15
20
10
20
30
Adj. Age

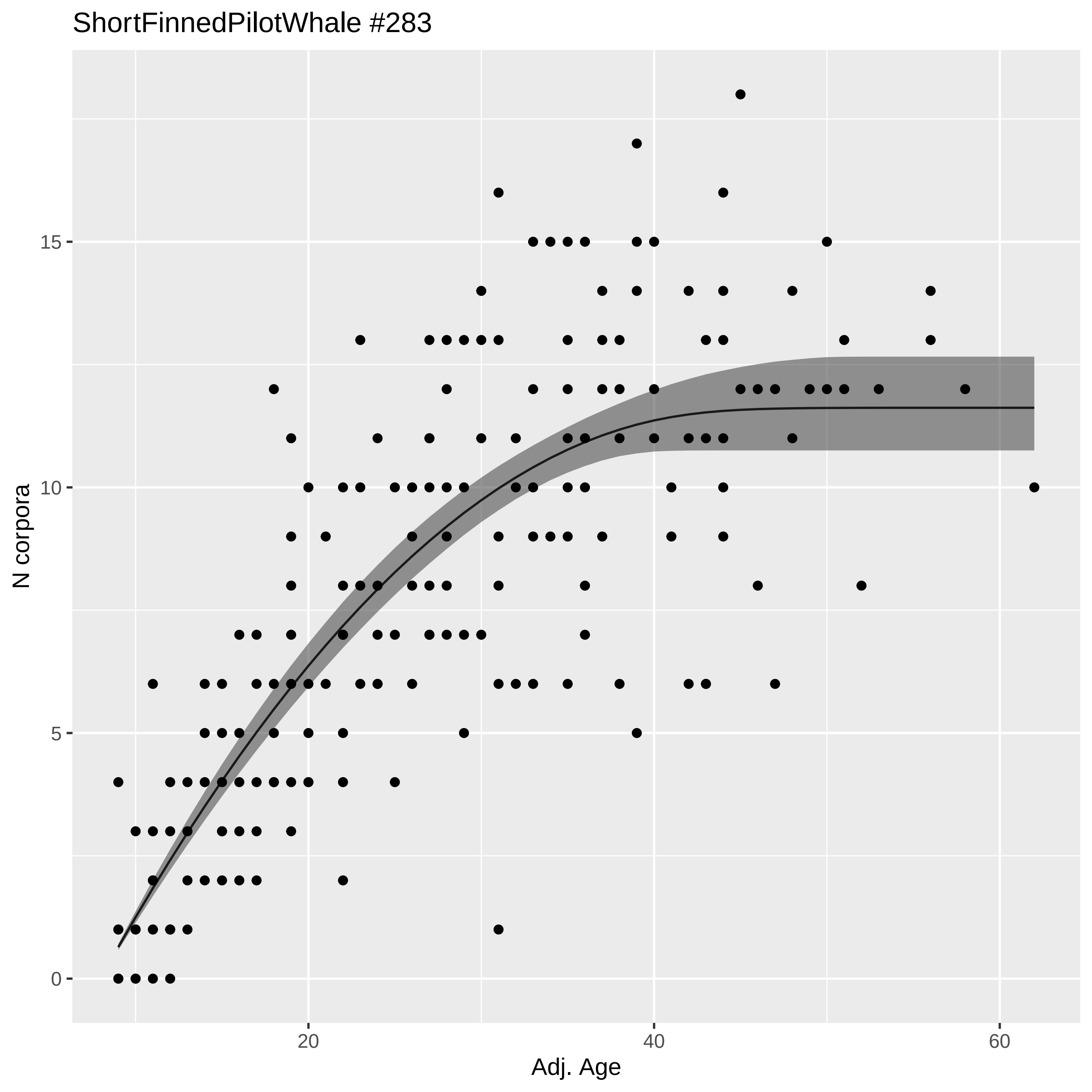
ShortFinnedPilotWhale #283
N corpora
0
5
10
15
20
40
60
Adj. Age

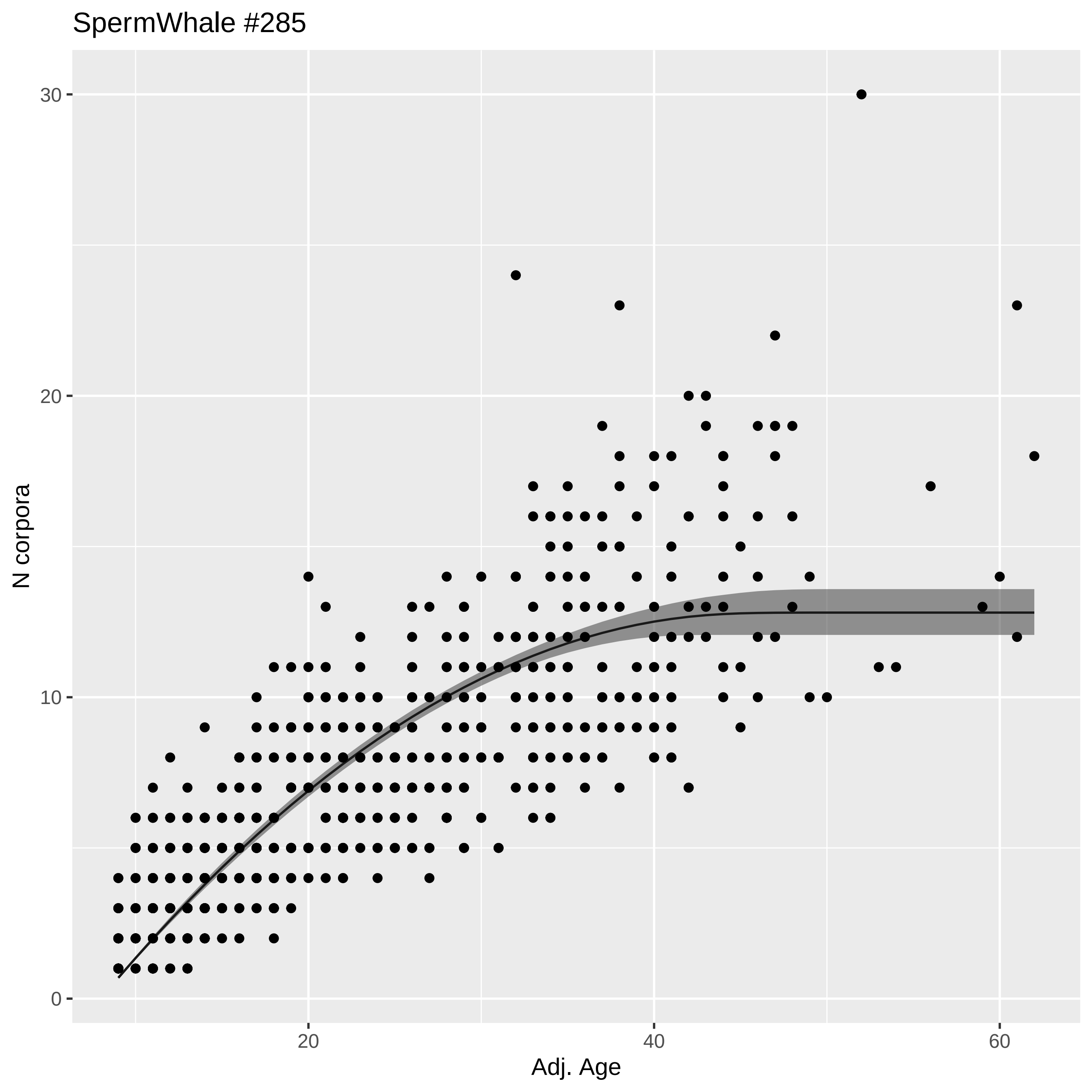
SpermWhale #285
N corpora
Adj. Age
0
10
20
30
20
40
60

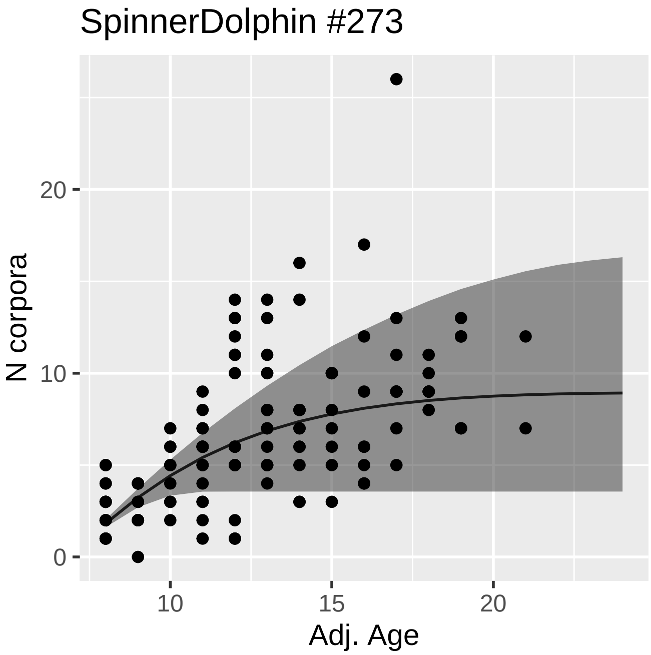
SpinnerDolphin #273
N corpora
0
10
20
10
15
20
Adj. Age

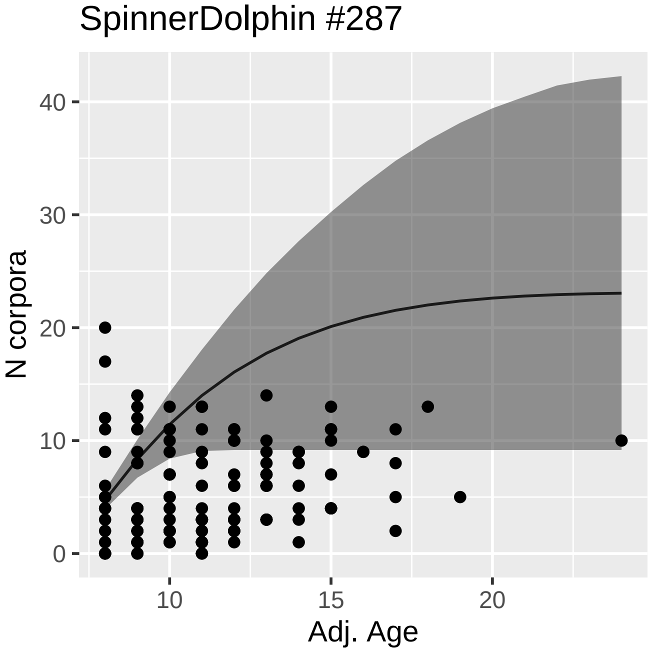
SpinnerDolphin #287
N corpora
0
10
20
30
40
10
15
20
Adj. Age

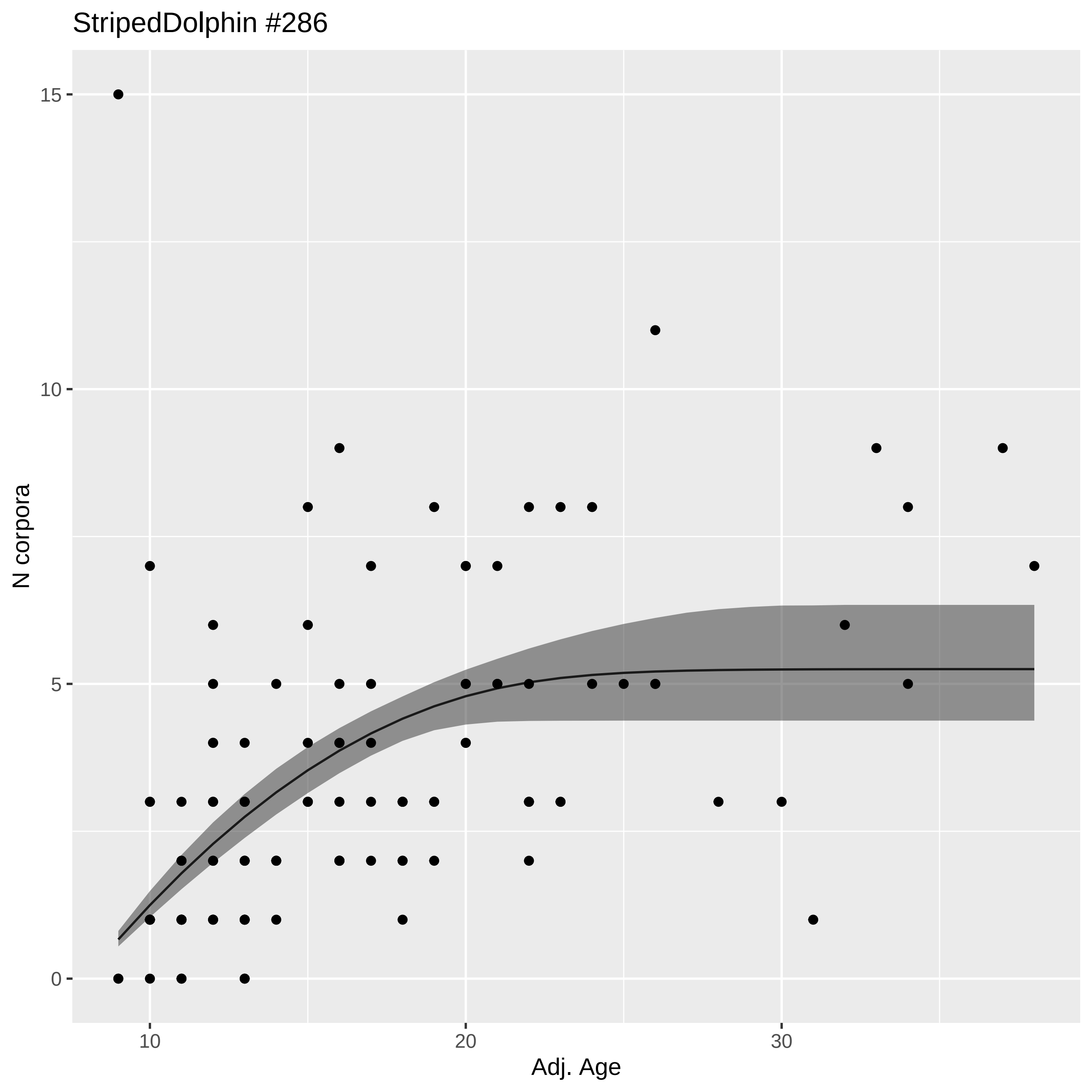
StripedDolphin #286
N corpora
15
10
5
0
10
20
30
Adj. Age

C. Fitted Mortality Models: Parameter Table

Parameter outputs from the lifespan models A single model is run for all of the data for one species. Parameters *a, b* are the fitted parameters for a Gompertz mortality model: where a model is run for both sexes *[1]* are the parameters for females and *[2]* for males. Population growth parameters *r* are run for each population of a species (so both sexes can be part of the same population and share a population growth parameter), indexed *[1 – n populations]* growth parameters show the fitted parameter for different populations. Sampling bias *s* applies to each dataset separately, datasets indexed *[1 – n datasets]*. Note that in some cases the particular parameter is not applied to a particular dataset (see methods)- in these cases the parameters have not been used for inference and just reflect the prior. For each parameter we report the posterior mean (post. Mean), standard deviation (post.sd), lower 95% credible interval (post.lCI) and upper 95% credible interview (post.uCI).

species	parameter	post.mean	post.sd	post.lCI	post.uCI
AtlanticSpottedDolphin	a[1]	0.045	0.016	0.022	0.072
	a[2]	0.039	0.014	0.019	0.064
	b[1]	0.231	0.060	0.140	0.332
	b[2]	0.211	0.059	0.123	0.309
	r[1]	0.011	0.279	-0.428	0.446
	s[1]	-0.009	1.092	-1.721	1.718
	s[2]	-0.004	1.086	-1.728	1.725
AtlanticWhiteSidedDolphin	a[1]	0.046	0.013	0.027	0.068
	a[2]	0.044	0.014	0.025	0.068
	b[1]	0.259	0.043	0.193	0.329
	b[2]	0.221	0.040	0.160	0.288
	r[1]	0.001	0.280	-0.433	0.440
	r[2]	0.002	0.275	-0.428	0.438
	r[3]	-0.241	0.134	-0.449	-0.013
	r[4]	0.006	0.284	-0.443	0.445
	s[1]	-0.968	0.452	-1.685	-0.224
	s[2]	-0.970	0.457	-1.693	-0.218
	s[3]	-0.971	0.469	-1.712	-0.182
	s[4]	-0.976	0.469	-1.713	-0.195
	s[5]	-0.010	1.100	-1.766	1.736
	s[6]	-0.014	1.092	-1.709	1.715
	s[7]	-0.001	1.064	-1.702	1.687
	s[8]	0.013	1.069	-1.697	1.724
Baiji	a[1]	0.038	0.013	0.020	0.061
	a[2]	0.039	0.013	0.020	0.061
	b[1]	0.160	0.031	0.112	0.213
	b[2]	0.206	0.045	0.135	0.282
	r[1]	-0.001	0.276	-0.436	0.438
	s[1]	-0.008	1.069	-1.693	1.706
	s[2]	-0.026	1.109	-1.754	1.728
BairdsBeakedWhale	a[1]	0.018	0.007	0.008	0.031
	a[2]	0.009	0.004	0.004	0.015
	b[1]	0.058	0.014	0.037	0.080
	b[2]	0.032	0.008	0.020	0.045
	r[1]	-0.238	0.129	-0.438	-0.024
	r[2]	-0.236	0.128	-0.434	-0.029
	r[3]	0.002	0.284	-0.435	0.437
	s[1]	0.011	1.074	-1.678	1.700
	s[2]	-0.008	1.037	-1.659	1.670
	s[3]	-0.023	1.070	-1.703	1.670
	s[4]	-0.009	1.087	-1.731	1.739
	s[5]	-0.024	1.103	-1.756	1.731
BelugaWhale	a[1]	0.021	0.003	0.017	0.026

species	parameter	post.mean	post.sd	post.lCI	post.uCI
	a[2]	0.031	0.005	0.023	0.039
	b[1]	0.039	0.004	0.033	0.046
	b[2]	0.035	0.006	0.026	0.044
	r[1]	0.001	0.280	-0.437	0.436
	r[2]	-0.001	0.277	-0.435	0.432
	r[3]	0.007	0.277	-0.431	0.436
	r[4]	0.002	0.282	-0.437	0.439
	r[5]	-0.004	0.282	-0.443	0.438
	r[6]	-0.236	0.127	-0.434	-0.023
	r[7]	-0.006	0.288	-0.449	0.448
	r[8]	0.006	0.282	-0.433	0.443
	s[1]	0.014	1.068	-1.652	1.714
	s[2]	-0.965	0.477	-1.715	-0.179
	s[3]	-0.997	0.482	-1.779	-0.207
	s[4]	-0.981	0.476	-1.724	-0.202
	s[5]	-0.968	0.467	-1.713	-0.201
	s[6]	0.026	1.056	-1.656	1.673
	s[7]	0.035	1.071	-1.687	1.702
	s[8]	-0.003	1.066	-1.694	1.696
	s[9]	0.008	1.090	-1.710	1.756
	s[10]	-0.011	1.084	-1.723	1.682
	s[11]	0.003	1.078	-1.697	1.722
	s[12]	0.004	1.065	-1.682	1.670
	s[13]	0.022	1.091	-1.701	1.734
ChileanDolphin	a[1]	0.037	0.013	0.019	0.060
	a[2]	0.043	0.014	0.023	0.068
	b[1]	0.193	0.043	0.126	0.263
	b[2]	0.227	0.057	0.142	0.324
	r[1]	-0.239	0.125	-0.434	-0.025
	s[1]	0.009	1.078	-1.703	1.722
	s[2]	0.007	1.092	-1.731	1.719
CommersonsDolphin	a[1]	0.048	0.014	0.027	0.072
	a[2]	0.037	0.012	0.021	0.058
	b[1]	0.235	0.044	0.168	0.306
	b[2]	0.159	0.029	0.115	0.206
	r[1]	-0.240	0.134	-0.450	-0.024
	r[2]	-0.238	0.127	-0.434	-0.023
	s[1]	0.004	1.071	-1.678	1.708
	s[2]	-0.001	1.101	-1.728	1.721
	s[3]	-0.004	1.070	-1.690	1.709
	s[4]	0.007	1.076	-1.703	1.698
CommonBottlenoseDolphin	a[1]	0.013	0.003	0.008	0.019
	a[2]	0.024	0.006	0.015	0.034

species	parameter	post.mean	post.sd	post.lCI	post.uCI
	b[1]	0.102	0.012	0.083	0.123
	b[2]	0.086	0.013	0.066	0.107
	r[1]	0.000	0.284	-0.440	0.436
	r[2]	0.001	0.279	-0.432	0.430
	r[3]	-0.003	0.280	-0.432	0.430
	r[4]	0.002	0.273	-0.430	0.422
	r[5]	0.000	0.277	-0.441	0.437
	r[6]	0.000	0.280	-0.440	0.436
	r[7]	0.001	0.280	-0.438	0.437
	r[8]	-0.006	0.278	-0.437	0.428
	r[9]	-0.211	0.169	-0.456	0.086
	r[10]	-0.209	0.161	-0.444	0.070
	r[11]	-0.210	0.165	-0.453	0.082
	r[12]	-0.212	0.171	-0.455	0.090
	s[1]	0.000	1.074	-1.702	1.703
	s[2]	0.007	1.095	-1.733	1.708
	s[3]	-0.002	1.082	-1.728	1.705
	s[4]	0.000	1.088	-1.710	1.688
	s[5]	0.004	1.108	-1.735	1.741
	s[6]	-0.001	1.089	-1.732	1.719
	s[7]	0.015	1.073	-1.706	1.721
	s[8]	-0.028	1.062	-1.704	1.699
	s[9]	0.007	1.076	-1.722	1.723
	s[10]	0.024	1.089	-1.705	1.722
	s[11]	-0.972	0.472	-1.718	-0.201
	s[12]	0.013	1.080	-1.707	1.715
	s[13]	0.000	1.055	-1.689	1.709
	s[14]	0.017	1.072	-1.692	1.725
	s[15]	0.005	1.071	-1.703	1.734
	s[16]	-0.017	1.074	-1.724	1.670
	s[17]	0.010	1.081	-1.722	1.717
	s[18]	0.008	1.073	-1.744	1.715
	s[19]	0.009	1.097	-1.729	1.741
	s[20]	-0.005	1.063	-1.709	1.683
CommonDolphin	a[1]	0.025	0.005	0.018	0.033
	a[2]	0.033	0.011	0.018	0.052
	b[1]	0.173	0.016	0.148	0.198
	b[2]	0.146	0.028	0.104	0.193
	r[1]	0.002	0.287	-0.447	0.447
	r[2]	-0.206	0.171	-0.454	0.095
	r[3]	-0.239	0.132	-0.443	-0.014
	r[4]	-0.212	0.167	-0.453	0.079
	r[5]	0.000	0.285	-0.434	0.439

species	parameter	post.mean	post.sd	post.lCI	post.uCI
	r[6]	-0.001	0.278	-0.438	0.435
	r[7]	-0.003	0.290	-0.450	0.446
	s[1]	0.974	0.473	0.189	1.721
	s[2]	0.977	0.475	0.187	1.720
	s[3]	0.002	1.080	-1.727	1.715
	s[4]	0.026	1.069	-1.680	1.712
	s[5]	0.022	1.069	-1.674	1.681
	s[6]	0.036	1.089	-1.718	1.759
	s[7]	0.011	1.079	-1.695	1.714
	s[8]	-0.031	1.077	-1.722	1.671
	s[9]	0.010	1.091	-1.726	1.726
CuviersBeakedWhale	a[1]	0.038	0.013	0.021	0.060
	a[2]	0.019	0.008	0.008	0.033
	b[1]	0.217	0.050	0.140	0.300
	b[2]	0.052	0.012	0.035	0.071
	r[1]	0.003	0.282	-0.441	0.442
	s[1]	-0.003	1.073	-1.666	1.707
	s[2]	-0.004	1.075	-1.692	1.692
DallsPorpoise	a[1]	0.252	0.019	0.223	0.283
	a[2]	0.149	0.017	0.124	0.177
	b[1]	0.117	0.015	0.092	0.141
	b[2]	0.179	0.019	0.148	0.210
	r[1]	-0.238	0.125	-0.437	-0.028
	r[2]	-0.236	0.130	-0.437	-0.012
	r[3]	-0.242	0.134	-0.447	-0.017
	r[4]	-0.236	0.123	-0.432	-0.033
	s[1]	0.012	1.067	-1.714	1.708
	s[2]	-0.029	1.091	-1.739	1.687
	s[3]	-0.001	1.047	-1.689	1.675
	s[4]	0.022	1.084	-1.697	1.712
	s[5]	0.002	1.096	-1.757	1.733
	s[6]	-0.973	0.458	-1.694	-0.232
	s[7]	-0.973	0.474	-1.705	-0.183
	s[8]	-0.004	1.103	-1.762	1.732
DuskyDolphin	a	0.020	0.008	0.008	0.035
	b	0.088	0.021	0.056	0.123
	r	-0.005	0.283	-0.443	0.436
	s	0.250	0.143	0.027	0.471
DwarfSpermWhale	a[1]	0.039	0.013	0.020	0.062
	a[2]	0.040	0.013	0.021	0.062
	b[1]	0.162	0.033	0.111	0.216
	b[2]	0.167	0.033	0.115	0.220
	r[1]	0.002	0.279	-0.440	0.435

species	parameter	post.mean	post.sd	post.lCI	post.uCI
	s[1]	0.003	1.063	-1.683	1.709
	s[2]	0.017	1.087	-1.693	1.718
FalseKillerWhale	a[1]	0.005	0.002	0.002	0.008
	a[2]	0.010	0.004	0.004	0.017
	b[1]	0.065	0.012	0.046	0.086
	b[2]	0.065	0.014	0.044	0.088
	r[1]	0.003	0.283	-0.436	0.439
	r[2]	-0.240	0.129	-0.440	-0.021
	s[1]	0.008	1.073	-1.687	1.695
	s[2]	0.024	1.073	-1.690	1.715
	s[3]	-0.017	1.089	-1.722	1.697
	s[4]	0.015	1.083	-1.705	1.723
Franciscana	a[1]	0.112	0.019	0.083	0.143
	a[2]	0.150	0.020	0.119	0.183
	b[1]	0.143	0.023	0.107	0.181
	b[2]	0.110	0.018	0.081	0.140
	r[1]	-0.238	0.132	-0.442	-0.018
	r[2]	-0.235	0.128	-0.434	-0.026
	r[3]	-0.236	0.130	-0.438	-0.023
	r[4]	-0.240	0.129	-0.443	-0.021
	r[5]	-0.236	0.126	-0.430	-0.024
	r[6]	-0.209	0.168	-0.454	0.081
	r[7]	-0.212	0.174	-0.461	0.089
	r[8]	-0.212	0.166	-0.456	0.068
	r[9]	-0.211	0.169	-0.452	0.082
	s[1]	-0.959	0.462	-1.672	-0.186
	s[2]	-0.981	0.471	-1.716	-0.200
	s[3]	0.972	0.458	0.233	1.702
	s[4]	0.978	0.487	0.161	1.733
	s[5]	-0.007	1.082	-1.710	1.696
	s[6]	0.008	1.051	-1.689	1.677
	s[7]	0.005	1.095	-1.741	1.733
	s[8]	0.032	1.084	-1.716	1.760
	s[9]	0.000	1.079	-1.703	1.714
	s[10]	0.013	1.092	-1.713	1.729
	s[11]	-0.003	1.068	-1.706	1.696
	s[12]	0.970	0.472	0.163	1.722
	s[13]	0.008	1.103	-1.720	1.720
	s[14]	0.974	0.456	0.230	1.708
FrasersDolphin	a[1]	0.031	0.010	0.017	0.048
	a[2]	0.043	0.013	0.025	0.065
	b[1]	0.230	0.041	0.166	0.299
	b[2]	0.241	0.042	0.176	0.310

species	parameter	post.mean	post.sd	post.lCI	post.uCI
	r[1]	0.003	0.280	-0.434	0.436
	r[2]	-0.007	0.281	-0.436	0.431
	r[3]	-0.237	0.130	-0.434	-0.017
	r[4]	0.002	0.282	-0.440	0.435
	s[1]	0.003	1.094	-1.745	1.743
	s[2]	-0.009	1.097	-1.742	1.712
	s[3]	-0.019	1.090	-1.724	1.744
	s[4]	-0.004	1.060	-1.695	1.706
	s[5]	-0.004	1.057	-1.698	1.664
	s[6]	-0.006	1.077	-1.697	1.680
	s[7]	0.002	1.060	-1.662	1.671
GangesRiverDolphin	a	0.031	0.013	0.013	0.054
	b	0.142	0.036	0.089	0.203
	r	0.010	0.256	-0.397	0.412
	s	0.232	0.146	0.020	0.467
GuianaDolphin	a[1]	0.020	0.008	0.010	0.033
	a[2]	0.035	0.011	0.019	0.053
	b[1]	0.101	0.021	0.069	0.136
	b[2]	0.105	0.021	0.074	0.140
	r[1]	-0.007	0.276	-0.435	0.429
	r[2]	0.005	0.272	-0.424	0.427
	r[3]	0.000	0.278	-0.434	0.435
	r[4]	0.001	0.280	-0.437	0.437
	r[5]	-0.210	0.167	-0.458	0.073
	r[6]	-0.211	0.163	-0.443	0.063
	r[7]	-0.002	0.289	-0.452	0.450
	r[8]	-0.003	0.286	-0.443	0.442
	s[1]	-0.029	1.087	-1.712	1.706
	s[2]	0.014	1.070	-1.684	1.673
	s[3]	-0.024	1.080	-1.719	1.719
	s[4]	-0.008	1.096	-1.718	1.740
	s[5]	0.005	1.078	-1.684	1.703
	s[6]	-0.015	1.082	-1.721	1.743
	s[7]	0.970	0.470	0.180	1.694
	s[8]	0.000	1.104	-1.724	1.738
	s[9]	-0.012	1.087	-1.707	1.704
	s[10]	-0.007	1.066	-1.672	1.707
	s[11]	0.003	1.087	-1.709	1.716
	s[12]	0.003	1.065	-1.684	1.731
HarbourPorpoise	a[1]	0.082	0.011	0.066	0.100
	a[2]	0.062	0.011	0.045	0.081
	b[1]	0.110	0.013	0.089	0.132
	b[2]	0.170	0.020	0.138	0.202

species	parameter	post.mean	post.sd	post.lCI	post.uCI
	r[1]	-0.240	0.132	-0.443	-0.020
	r[2]	-0.002	0.277	-0.434	0.432
	r[3]	-0.209	0.169	-0.453	0.082
	r[4]	-0.208	0.168	-0.452	0.083
	r[5]	-0.211	0.165	-0.454	0.079
	r[6]	-0.211	0.170	-0.459	0.084
	r[7]	0.001	0.278	-0.438	0.435
	r[8]	-0.006	0.288	-0.448	0.444
	r[9]	-0.009	0.283	-0.441	0.437
	r[10]	0.005	0.284	-0.435	0.441
	r[11]	0.002	0.283	-0.438	0.443
	r[12]	-0.002	0.282	-0.438	0.438
	r[13]	-0.003	0.283	-0.442	0.441
	r[14]	0.001	0.286	-0.440	0.444
	r[15]	-0.003	0.282	-0.440	0.436
	r[16]	0.002	0.284	-0.439	0.444
	r[17]	0.007	0.279	-0.437	0.439
	r[18]	-0.002	0.277	-0.440	0.431
	r[19]	0.001	0.288	-0.449	0.448
	r[20]	-0.003	0.284	-0.444	0.444
	s[1]	-0.008	1.081	-1.735	1.714
	s[2]	0.019	1.082	-1.679	1.724
	s[3]	0.015	1.105	-1.710	1.737
	s[4]	0.020	1.091	-1.721	1.734
	s[5]	-0.007	1.077	-1.735	1.707
	s[6]	0.008	1.098	-1.724	1.742
	s[7]	-0.013	1.043	-1.707	1.633
	s[8]	0.005	1.085	-1.722	1.720
	s[9]	-0.039	1.105	-1.748	1.721
	s[10]	-0.004	1.070	-1.694	1.688
	s[11]	-0.015	1.101	-1.717	1.751
	s[12]	-0.015	1.069	-1.691	1.709
	s[13]	-0.007	1.066	-1.671	1.670
	s[14]	0.005	1.074	-1.688	1.701
	s[15]	-0.006	1.079	-1.713	1.701
	s[16]	-0.004	1.111	-1.727	1.739
	s[17]	0.015	1.067	-1.680	1.713
	s[18]	0.010	1.066	-1.685	1.686
	s[19]	-0.009	1.068	-1.713	1.705
	s[20]	0.000	1.076	-1.686	1.688
	s[21]	0.012	1.086	-1.685	1.722
	s[22]	0.025	1.079	-1.724	1.713
	a[1]	0.036	0.013	0.018	0.059

species	parameter	post.mean	post.sd	post.lCI	post.uCI
	a[2]	0.032	0.012	0.017	0.053
	b[1]	0.188	0.042	0.124	0.257
	b[2]	0.181	0.041	0.118	0.249
	r[1]	-0.237	0.130	-0.439	-0.016
	s[1]	-0.979	0.465	-1.718	-0.218
	s[2]	-0.968	0.466	-1.689	-0.212
IndianOceanHumpbackDolphin	a[1]	0.040	0.013	0.022	0.063
	a[2]	0.035	0.012	0.019	0.056
	b[1]	0.232	0.054	0.150	0.321
	b[2]	0.181	0.036	0.125	0.242
	r[1]	-0.235	0.126	-0.431	-0.027
	s[1]	0.022	1.093	-1.707	1.757
	s[2]	-0.006	1.089	-1.716	1.712
IndoPacificBottlenoseDolphin	a[1]	0.038	0.011	0.022	0.056
	a[2]	0.029	0.009	0.016	0.045
	b[1]	0.074	0.015	0.051	0.100
	b[2]	0.099	0.019	0.070	0.131
	r[1]	0.000	0.291	-0.445	0.449
	r[2]	-0.001	0.287	-0.449	0.441
	r[3]	-0.240	0.130	-0.442	-0.027
	s[1]	0.014	1.084	-1.715	1.716
	s[2]	0.016	1.067	-1.696	1.704
	s[3]	0.012	1.096	-1.720	1.728
	s[4]	0.012	1.064	-1.703	1.703
IndoPacificFinlessPorpoise	a[1]	0.029	0.011	0.014	0.048
	a[2]	0.034	0.012	0.017	0.055
	b[1]	0.094	0.020	0.063	0.127
	b[2]	0.181	0.043	0.116	0.253
	r[1]	0.002	0.280	-0.436	0.434
	s[1]	0.025	1.077	-1.720	1.721
	s[2]	-0.001	1.065	-1.706	1.727
IndoPacificHumpbackDolphin	a[1]	0.015	0.006	0.007	0.026
	a[2]	0.030	0.011	0.016	0.049
	b[1]	0.105	0.023	0.070	0.143
	b[2]	0.137	0.027	0.095	0.182
	r[1]	-0.001	0.281	-0.435	0.440
	r[2]	0.004	0.277	-0.431	0.440
	s[1]	-0.011	1.074	-1.703	1.707
	s[2]	0.018	1.070	-1.710	1.719
	s[3]	0.036	1.085	-1.712	1.731
	s[4]	0.008	1.086	-1.735	1.722
KillerWhale	a[1]	0.026	0.006	0.017	0.036
	a[2]	0.031	0.009	0.017	0.047

species	parameter	post.mean	post.sd	post.lCI	post.uCI
	b[1]	0.028	0.006	0.019	0.038
	b[2]	0.087	0.017	0.060	0.116
	r[1]	-0.001	0.285	-0.441	0.440
	r[2]	-0.237	0.134	-0.443	-0.018
	r[3]	-0.239	0.136	-0.449	-0.014
	r[4]	-0.007	0.279	-0.445	0.435
	r[5]	0.000	0.289	-0.448	0.448
	r[6]	-0.001	0.274	-0.433	0.434
	r[7]	0.003	0.279	-0.432	0.437
	r[8]	-0.002	0.280	-0.436	0.433
	s[1]	-0.002	1.081	-1.677	1.717
	s[2]	0.007	1.081	-1.739	1.715
	s[3]	-0.005	1.093	-1.746	1.724
	s[4]	-0.971	0.462	-1.697	-0.212
	s[5]	-0.976	0.454	-1.700	-0.213
	s[6]	-0.014	1.065	-1.694	1.689
	s[7]	0.000	1.082	-1.726	1.727
	s[8]	-0.008	1.095	-1.747	1.751
	s[9]	0.007	1.077	-1.692	1.713
	s[10]	0.013	1.109	-1.717	1.734
	s[11]	-0.009	1.050	-1.685	1.677
LongFinnedPilotWhale	a[1]	0.013	0.002	0.010	0.016
	a[2]	0.023	0.005	0.016	0.032
	b[1]	0.075	0.006	0.065	0.086
	b[2]	0.088	0.012	0.070	0.107
	r[1]	-0.235	0.129	-0.432	-0.020
	r[2]	-0.006	0.277	-0.436	0.431
	r[3]	0.000	0.279	-0.435	0.436
	s[1]	0.015	1.088	-1.703	1.731
	s[2]	0.014	1.098	-1.738	1.722
	s[3]	-0.008	1.093	-1.734	1.725
	s[4]	-0.003	1.080	-1.713	1.693
	s[5]	-0.020	1.106	-1.746	1.715
	s[6]	0.003	1.074	-1.735	1.692
MelonHeadedWhale	a[1]	0.012	0.004	0.006	0.020
	a[2]	0.013	0.006	0.006	0.023
	b[1]	0.092	0.017	0.067	0.120
	b[2]	0.116	0.024	0.080	0.155
	r[1]	0.000	0.276	-0.429	0.432
	r[2]	0.003	0.277	-0.434	0.440
	r[3]	0.002	0.278	-0.440	0.429
	r[4]	0.003	0.281	-0.437	0.443
	r[5]	-0.003	0.279	-0.439	0.434

species	parameter	post.mean	post.sd	post.lCI	post.uCI
	r[6]	-0.002	0.281	-0.439	0.431
	s[1]	0.013	1.085	-1.726	1.721
	s[2]	0.009	1.063	-1.668	1.694
	s[3]	0.018	1.088	-1.704	1.738
	s[4]	-0.010	1.060	-1.701	1.689
	s[5]	-0.974	0.478	-1.730	-0.185
	s[6]	-0.979	0.475	-1.735	-0.201
	s[7]	-0.011	1.081	-1.716	1.719
	s[8]	0.000	1.087	-1.698	1.716
NarrowRidgedFinlessPorpoise	a[1]	0.038	0.011	0.022	0.057
	a[2]	0.033	0.010	0.019	0.051
	b[1]	0.135	0.024	0.097	0.176
	b[2]	0.155	0.026	0.115	0.197
	r[1]	-0.002	0.281	-0.439	0.437
	r[2]	-0.237	0.131	-0.438	-0.012
	r[3]	-0.238	0.129	-0.439	-0.021
	r[4]	0.002	0.279	-0.436	0.436
	r[5]	-0.207	0.175	-0.456	0.098
	r[6]	-0.212	0.165	-0.451	0.072
	r[7]	-0.210	0.164	-0.448	0.067
	r[8]	-0.209	0.169	-0.450	0.085
	s[1]	-0.007	1.085	-1.725	1.698
	s[2]	0.014	1.073	-1.686	1.704
	s[3]	-0.005	1.070	-1.707	1.693
	s[4]	0.002	1.105	-1.734	1.744
	s[5]	0.024	1.095	-1.708	1.721
	s[6]	0.015	1.080	-1.698	1.721
	s[7]	-0.009	1.083	-1.719	1.691
	s[8]	-0.006	1.073	-1.696	1.684
	s[9]	-0.975	0.474	-1.735	-0.205
	s[10]	-0.981	0.457	-1.711	-0.246
	s[11]	0.003	1.085	-1.715	1.742
	s[12]	0.001	1.081	-1.720	1.693
Narwhal	a[1]	0.012	0.004	0.006	0.018
	a[2]	0.017	0.005	0.010	0.025
	b[1]	0.028	0.006	0.019	0.039
	b[2]	0.029	0.006	0.019	0.039
	r[1]	0.003	0.282	-0.434	0.435
	r[2]	-0.002	0.284	-0.447	0.445
	r[3]	0.004	0.284	-0.438	0.437
	r[4]	-0.006	0.288	-0.447	0.440
	r[5]	0.005	0.279	-0.435	0.435
	r[6]	0.001	0.289	-0.442	0.447

species	parameter	post.mean	post.sd	post.lCI	post.uCI
	s[1]	0.023	1.060	-1.669	1.700
	s[2]	-0.007	1.078	-1.710	1.703
	s[3]	-0.009	1.080	-1.694	1.684
	s[4]	-0.013	1.066	-1.683	1.689
	s[5]	0.003	1.087	-1.698	1.715
	s[6]	-0.006	1.056	-1.704	1.707
	a[1]	0.033	0.011	0.018	0.052
	a[2]	0.025	0.008	0.014	0.040
	b[1]	0.169	0.032	0.120	0.224
NorthernBottlenoseWhale	b[2]	0.095	0.018	0.067	0.125
	r[1]	-0.241	0.127	-0.439	-0.028
	s[1]	0.005	1.088	-1.732	1.730
	s[2]	-0.009	1.091	-1.722	1.697
	a[1]	0.040	0.011	0.024	0.058
	a[2]	0.058	0.015	0.035	0.085
	b[1]	0.115	0.020	0.083	0.148
	b[2]	0.144	0.025	0.105	0.184
	r[1]	-0.234	0.132	-0.439	-0.012
NorthernRightWhaleDolphin	r[2]	-0.239	0.129	-0.438	-0.022
	s[1]	-0.019	1.078	-1.721	1.716
	s[2]	-0.013	1.093	-1.732	1.739
	s[3]	-0.965	0.467	-1.719	-0.212
	s[4]	-0.975	0.464	-1.693	-0.197
	a[1]	0.017	0.006	0.008	0.027
	a[2]	0.015	0.006	0.007	0.025
	b[1]	0.088	0.016	0.063	0.115
	b[2]	0.107	0.020	0.077	0.140
	r[1]	0.001	0.274	-0.426	0.430
	r[2]	-0.238	0.132	-0.443	-0.014
PacificWhiteSidedDolphin	r[3]	-0.236	0.126	-0.435	-0.025
	s[1]	0.010	1.058	-1.682	1.679
	s[2]	0.009	1.095	-1.706	1.738
	s[3]	-0.966	0.468	-1.690	-0.187
	s[4]	-0.978	0.470	-1.710	-0.216
	s[5]	0.019	1.052	-1.660	1.686
	s[6]	-0.012	1.087	-1.728	1.706
	a[1]	0.016	0.003	0.011	0.020
	a[2]	0.016	0.004	0.010	0.024
	b[1]	0.127	0.010	0.111	0.144
PantropicalSpottedDolphin	b[2]	0.156	0.018	0.129	0.185
	r[1]	-0.237	0.130	-0.435	-0.019
	r[2]	-0.211	0.170	-0.454	0.084
	r[3]	-0.210	0.167	-0.455	0.073

species	parameter	post.mean	post.sd	post.lCI	post.uCI
	r[4]	-0.209	0.171	-0.458	0.088
	r[5]	-0.212	0.166	-0.455	0.077
	r[6]	-0.209	0.170	-0.456	0.091
	r[7]	-0.211	0.169	-0.456	0.077
	r[8]	-0.210	0.174	-0.457	0.093
	r[9]	-0.207	0.164	-0.449	0.078
	s[1]	-0.985	0.480	-1.756	-0.198
	s[2]	-0.967	0.467	-1.702	-0.187
	s[3]	-0.965	0.462	-1.701	-0.206
	s[4]	-0.972	0.481	-1.734	-0.164
	s[5]	-0.968	0.476	-1.730	-0.186
	s[6]	-0.967	0.463	-1.697	-0.169
	s[7]	-0.968	0.459	-1.695	-0.192
	s[8]	-0.963	0.462	-1.685	-0.188
	s[9]	-0.978	0.472	-1.728	-0.211
	s[10]	-0.988	0.467	-1.715	-0.217
PealesDolphin	a[1]	0.040	0.015	0.020	0.066
	a[2]	0.039	0.014	0.019	0.064
	b[1]	0.217	0.055	0.133	0.309
	b[2]	0.209	0.056	0.126	0.302
	r[1]	0.000	0.278	-0.437	0.438
	s[1]	-0.022	1.058	-1.714	1.665
	s[2]	0.028	1.073	-1.685	1.728
PygmySpermWhale	a[1]	0.034	0.012	0.018	0.055
	a[2]	0.032	0.011	0.016	0.052
	b[1]	0.155	0.031	0.108	0.205
	b[2]	0.105	0.022	0.072	0.140
	r[1]	-0.002	0.277	-0.435	0.433
	r[2]	0.001	0.281	-0.444	0.440
	r[3]	0.001	0.279	-0.428	0.439
	r[4]	-0.002	0.292	-0.449	0.443
	s[1]	0.976	0.482	0.180	1.726
	s[2]	0.971	0.462	0.209	1.687
	s[3]	0.980	0.490	0.162	1.733
	s[4]	-0.013	1.093	-1.742	1.739
	s[5]	0.001	1.091	-1.719	1.701
RissosDolphin	a[1]	0.024	0.008	0.012	0.038
	a[2]	0.036	0.012	0.019	0.058
	b[1]	0.106	0.020	0.074	0.140
	b[2]	0.162	0.034	0.110	0.218
	r[1]	0.000	0.281	-0.433	0.433
	r[2]	-0.240	0.130	-0.442	-0.020
	r[3]	-0.238	0.129	-0.438	-0.021

species	parameter	post.mean	post.sd	post.lCI	post.uCI
	r[4]	-0.001	0.278	-0.434	0.429
	s[1]	-0.003	1.057	-1.658	1.703
	s[2]	-0.002	1.082	-1.740	1.707
	s[3]	-0.967	0.472	-1.700	-0.201
	s[4]	0.004	1.084	-1.715	1.715
	s[5]	0.003	1.088	-1.705	1.734
	s[6]	-0.002	1.108	-1.742	1.751
	s[7]	0.974	0.471	0.212	1.730
	s[8]	0.969	0.462	0.213	1.707
RoughToothedDolphin	a[1]	0.021	0.008	0.009	0.036
	a[2]	0.027	0.010	0.013	0.043
	b[1]	0.101	0.023	0.066	0.140
	b[2]	0.138	0.028	0.094	0.184
	r[1]	0.002	0.284	-0.443	0.442
	r[2]	-0.242	0.130	-0.443	-0.026
	s[1]	0.012	1.085	-1.718	1.732
	s[2]	-0.003	1.064	-1.701	1.713
	s[3]	-0.014	1.060	-1.702	1.671
	s[4]	-0.018	1.076	-1.718	1.705
ShortFinnedPilotWhale	a[1]	0.008	0.002	0.005	0.012
	a[2]	0.027	0.007	0.017	0.039
	b[1]	0.068	0.008	0.055	0.081
	b[2]	0.080	0.014	0.059	0.102
	r[1]	-0.240	0.128	-0.442	-0.027
	r[2]	-0.236	0.130	-0.439	-0.023
	r[3]	-0.008	0.272	-0.436	0.430
	s[1]	-0.960	0.467	-1.699	-0.203
	s[2]	-0.994	0.483	-1.744	-0.201
	s[3]	-0.016	1.094	-1.732	1.679
	s[4]	-0.006	1.076	-1.706	1.695
	s[5]	-0.022	1.083	-1.720	1.712
	s[6]	0.002	1.064	-1.716	1.702
SpermWhale	a[1]	0.024	0.002	0.021	0.028
	a[2]	0.014	0.002	0.012	0.017
	b[1]	0.042	0.003	0.037	0.046
	b[2]	0.055	0.004	0.049	0.061
	r[1]	-0.210	0.161	-0.451	0.067
	r[2]	-0.002	0.283	-0.440	0.439
	r[3]	-0.212	0.167	-0.454	0.071
	r[4]	-0.207	0.164	-0.451	0.077
	r[5]	0.003	0.278	-0.435	0.438
	r[6]	-0.211	0.171	-0.457	0.083
	r[7]	-0.211	0.168	-0.453	0.079

species	parameter	post.mean	post.sd	post.lCI	post.uCI
	r[8]	-0.209	0.167	-0.451	0.084
	r[9]	-0.207	0.167	-0.455	0.084
	s[1]	-0.976	0.472	-1.708	-0.202
	s[2]	0.002	1.089	-1.729	1.741
	s[3]	0.007	1.087	-1.706	1.722
	s[4]	-0.022	1.080	-1.743	1.713
	s[5]	-0.016	1.076	-1.734	1.704
	s[6]	-0.976	0.482	-1.740	-0.179
	s[7]	-0.038	1.096	-1.742	1.731
	s[8]	-0.969	0.467	-1.706	-0.205
	s[9]	-0.975	0.466	-1.712	-0.209
	s[10]	0.004	1.050	-1.704	1.680
SpinnerDolphin	a[1]	0.035	0.005	0.027	0.043
	a[2]	0.059	0.014	0.038	0.082
	b[1]	0.193	0.015	0.171	0.216
	b[2]	0.241	0.033	0.189	0.296
	r[1]	-0.209	0.172	-0.457	0.091
	r[2]	-0.208	0.176	-0.465	0.095
	r[3]	-0.206	0.167	-0.451	0.079
	r[4]	-0.210	0.167	-0.455	0.077
	s[1]	-0.015	1.065	-1.718	1.684
	s[2]	0.002	1.078	-1.693	1.701
	s[3]	0.017	1.069	-1.706	1.712
	s[4]	0.013	1.091	-1.733	1.747
StejnegersBeakedWhale	a	0.023	0.009	0.010	0.039
	b	0.118	0.029	0.074	0.165
	r	-0.003	0.284	-0.436	0.442
	s	0.250	0.145	0.026	0.471
StripedDolphin	a[1]	0.029	0.004	0.022	0.036
	a[2]	0.062	0.008	0.049	0.074
	b[1]	0.111	0.010	0.096	0.128
	b[2]	0.060	0.008	0.047	0.074
	r[1]	-0.235	0.129	-0.437	-0.016
	r[2]	0.000	0.281	-0.442	0.443
	s[1]	-0.027	1.097	-1.738	1.729
	s[2]	-0.007	1.092	-1.742	1.719
	s[3]	0.012	1.090	-1.710	1.741
	s[4]	0.029	1.085	-1.709	1.747
Vaquita	a[1]	0.028	0.010	0.014	0.046
	a[2]	0.030	0.011	0.015	0.049
	b[1]	0.167	0.036	0.112	0.228
	b[2]	0.188	0.044	0.122	0.260
	r[1]	-0.238	0.129	-0.432	-0.025

species	parameter	post.mean	post.sd	post.lCI	post.uCI
	s[1]	-0.970	0.477	-1.722	-0.170
	s[2]	-0.974	0.458	-1.700	-0.224
WhiteBeakedDolphin	a[1]	0.022	0.008	0.011	0.035
	a[2]	0.032	0.011	0.017	0.051
	b[1]	0.093	0.018	0.065	0.123
	b[2]	0.113	0.023	0.078	0.153
	r[1]	-0.004	0.282	-0.439	0.440
	r[2]	-0.004	0.276	-0.437	0.435
	r[3]	0.002	0.273	-0.437	0.438
	s[1]	0.003	1.089	-1.723	1.734
	s[2]	-0.016	1.075	-1.717	1.703
	s[3]	0.009	1.115	-1.756	1.766
	s[4]	0.012	1.076	-1.677	1.691
	s[5]	-0.007	1.074	-1.714	1.694
	s[6]	-0.008	1.083	-1.735	1.711

D. Fitted Corpora Models: Parameter Tables

Parameter estimates for fitted reproductive models. For each species rate of corpora deposition (*alpha*) can vary between datasets, while rate of deposition rate (*beta*) is the same. Alpha values for different datasets of the same species are indexed *[1 – n datasets]*.

species	variable	mean	sd	lCI	uCI
AtlanticWhiteSidedDolphin	alpha	4.35	0.44	3.68	5.09
	beta	0.14	0.02	0.11	0.19
BairdsBeakedWhale	alpha	0.76	0.08	0.64	0.90
	beta	0.02	0.00	0.02	0.03
BelugaWhale	alpha[1]	0.62	0.04	0.56	0.69
	alpha[2]	0.49	0.04	0.42	0.57
	beta	0.03	0.02	0.02	0.08
CommonBottlenoseDolphin	alpha[1]	0.78	0.05	0.69	0.87
	alpha[2]	0.82	0.06	0.74	0.93
	alpha[3]	0.69	0.08	0.57	0.82
	beta	0.03	0.01	0.02	0.05
CommonDolphin	alpha[1]	3.76	0.25	3.35	4.19
	alpha[2]	1.39	0.08	1.25	1.53
	beta	0.08	0.03	0.04	0.16
DallsPorpoise	alpha	0.19	0.04	0.13	0.26
	beta	0.12	0.03	0.10	0.21
FalseKillerWhale	alpha[1]	0.59	0.06	0.50	0.69
	alpha[2]	0.75	0.28	0.43	1.30
	beta	0.03	0.02	0.01	0.08
HarbourPorpoise	alpha[1]	2.72	0.33	2.19	3.30
	alpha[2]	1.26	0.19	0.98	1.61
	alpha[3]	1.05	0.18	0.80	1.36
	beta	0.09	0.03	0.05	0.18
IndoPacificBottlenoseDolphin	alpha	2.28	0.35	1.76	2.90
	beta	0.14	0.03	0.09	0.20
LongFinnedPilotWhale	alpha	0.51	0.02	0.48	0.54
	beta	0.02	0.00	0.02	0.03
MelonHeadedWhale	alpha[1]	0.66	0.08	0.55	0.79
	alpha[2]	0.39	0.05	0.31	0.48
	beta	0.03	0.02	0.02	0.08
Narwhal	alpha	0.42	0.05	0.35	0.51
	beta	0.02	0.00	0.02	0.03
NorthernRightWhaleDolphin	alpha	1.15	0.06	1.06	1.25
	beta	0.04	0.00	0.03	0.04
PantropicalSpottedDolphin	alpha	0.90	0.03	0.85	0.96
	beta	0.04	0.00	0.03	0.05
ShortFinnedPilotWhale	alpha	0.64	0.04	0.58	0.71
	beta	0.03	0.00	0.02	0.03
SpermWhale	alpha	0.69	0.02	0.66	0.72
	beta	0.03	0.00	0.02	0.03
SpinnerDolphin	alpha[1]	1.82	0.13	1.62	2.05
	alpha[2]	4.72	0.50	3.93	5.63
	beta	0.12	0.05	0.05	0.25

StripedDolphin	alpha	0.67	0.08	0.55	0.81
	beta	0.06	0.01	0.04	0.09

Supplementary 3: Additional Data Explanation

Here we present further explanation of the origins and derivation of the phylogeny, size, age at maturity and survival to maturity data used in this study.

Phylogeny

We derive the structure and time-calibration of the phylogeny used in this analysis from a recently published toothed whale phylogeny[1]. We run analyses over 1000 bootstrapped phylogenies, combining the posteriors from these models to derive the parsimonious parameter estimates over various bootstrapped tree structures. The bootstrapped trees only differ over a small number of nodes- and in general, the phylogenetic structure of the whales is well resolved (figure 1; figure SE1).

We time-calibrate trees using the mean estimated divergence times from an analysis published alongside the tree structures[1]. Under some bootstrapped tree structures species, the mean divergence times are not possible given the tree structure. This only occurs at certain 7 nodes, and when in these trees we infer the node divergence times via correlated penalised likelihood estimation[2] implemented via the ape R package[3]. The result is 1000 time-calibrated bootstrapped tree structures. For computational reasons, we limit analysis to a single consensus tree for some supplementary analysis (table S3). This consensus tree is from the same source as the bootstrapped trees[1] and time-calibrated in the same way as described above.

Thirteen recognised toothed whale species[4] are not present in our phylogenies (table SE1). For these 13 species, a sister species was chosen based on presumed phylogenetic history from other sources (table SE1). The missing species are assumed to have the same phylogenetic position as their sister species. In essence, this assumes that the missing species and their sister species have an identical phylogenetic history. Most of the missing species and their sister species have only recently been recognised as separate species, or are known to be close relatives with poorly resolved inter-relations, the sister species are likely therefore to offer a very good approximation of their true phylogenetic position. Two missing species – Longman's beaked whale (*Indopacetus pacificus*) and spade-toothed beaked whales (*Mesoplodon traversii*)– are very little known and their nearest phylogenetic relatives are not resolved with any certainty and their sister species are therefore necessarily speculative. However, as neither Longman's beaked whales, spade-toothed beaked whale or their sister species, nor any of their near phylogenetic relatives appear in any further analysis this uncertainty will not affect any of the results in this study.

Age at Maturity

For the species in our analysis, we require age at maturity to (1) define the beginning of adulthood for our mortality model (Methods: Data: lifespan data and modelling), and (2) for some regression analyses (Methods: Analysis: Intergenerational help and the evolution of extended lifespan). For most species-sexes, we collated age at maturity estimates from expert analysis of available evidence[5], but where this is unavailable we referred to individual studies[6–12]. For each species, we gathered as many as available of: mean, minimum, maximum and standard deviation around, age at maturity. When not specified we assume a single reported 'age at maturity' for a species-sex represents the mean. We processed the data to get a mean and standard deviation for each species. When not reported the mean was assumed to be mid-point between the minimum and maximum reported ages at maturity. When not reported, the standard deviation was calculated from the assumption that the range between the minimum and maximum reported ages at maturity represents the 95% - and therefore 4 standard deviations - of the distribution of ages at maturity. All collected and reported age at maturity metrics are available in the marinelifehistory R package[13]. When defining the beginning of adulthood for the mortality model we use the mean age at maturity. For other analyses, we use the age with the standard deviation.

Size

Measures

Although mass is the most common measure of size used in life-history studies, for cetaceans accurate mass data are rare. Mass is difficult to measure in cetaceans for two reasons (1) it is logistically difficult to weigh, large, free-ranging, marine mammals and (2) as most samples are necessarily of deceased whales the time since death and cause of death can both influence realised weight. Due to these limitations mass samples are almost always small and are potentially difficult to compare between samples and studies[14]. Due to these difficulties- and consistent with previous work and recommendations[14] – we use length as our measure of size throughout this study.

We also collected and processed mass data using the same protocol as described for length below. In these samples there is a strong positive correlation between length and mass, supporting the robustness of length as a measure of size in toothed whales (figure SE2).

Data collection and processing

Length data were collated from expert evaluation in edited volumes (primarily the Handbook of Marine Mammals series[15–17], supplemented by the Encyclopaedia of Marine Mammals[5] where data are not available in the Handbooks). We collated all available summary metrics for every toothed whale species-sex (table SE2). We classified these metrics as: mean, asymptote, standard deviation, minimum or maximum. Asymptotes represent the asymptotic length from fitted growth models, for the rest of this data processing pathway asymptotes are considered synonymous with means and subject to the same processing. As data are sometimes reported from multiple populations in our data it was not uncommon to have multiple measures of some metrics for a given species-sex.

We first collated these metrics to get a single measure of each metric for every species-sex. For each species-sex, the metrics were collated as follows:

1. Means. The mean of reported means and asymptotes, weighted by sample size where possible (i.e. if all sample sizes were reported).
2. Standard deviations. The mean of reported standard deviations, weighted by sample size where possible.
3. Minimum. The minimum of reported minimums.
4. Maximum. The maximum of reported maximums.

In some cases, where no data for any metrics were available for a given species-sex, we filled the gap by: (a) using data from combined sexes samples (6 species in the final sample [figure 1]: dwarf sperm whale *Kogia sima*, Franciscana *Pontoporia blainvillei*, Guiana dolphin *Sotalia guianensis*, Northern right-whale dolphin *Lissodelphis borealis*, Pacific white-sided dolphin *Lagenorhynchus obliquidens*, pygmy sperm whale *Kogia breviceps*) and if that is not possible (b) using the data from the other sex (1 species in analysed sample [figure 1]: Commerson's dolphin *Cephalorhynchus commersonii*). Species with size estimates derived from combined sex or opposite sex samples are not included in analyses including or comparing both sexes.

From these collated metrics we then calculated a species-mean and species-standard deviation for each species. Where mean or standard deviation were available for a given species-sex they were used as the species metrics. Where either mean and/or standard deviation were not available they were calculated from the other metrics. For the mean of a given species-sex, where a collated mean was unavailable (n=10/32), we used in order of preference: (1) if minimum and maximum estimates are available we used the mid-point of this range; (2) if only one of the minimum or maximum were available we used the scaled range (between minimum and maximum) for known species-sex pairs and then applied this to the species in question to get an estimate of the missing metric, and then took

the mid-point as the mean. For the standard deviation of a given species-sex, where the collated standard deviation was unavailable (n = 23/32), we used in order of preference (1) if minimum and maximum are both available and the known total sample size used to get the observed metrics >30 then we assume the that the range covers 95% of the true variation and therefore represents four standard deviations; (2) if minimum and maximum are unavailable but total sample size ≤30 then we assume that the sample represents 50% of the true variation and therefore is two standard deviations and (3) if either minimum or maximum are unavailable we use the scaled range to calculate the missing metric (as for the mean) and then repeat standard deviation steps 1 and 2. The outcome of this pathway is a measure of mean and standard deviation for every toothed whale species in this study. Both raw and derived data are available in the marinelifehistdata package[13].

Survival to maturity

For our demographic simulations, we required an estimate of survival to maturity for all the toothed whales in our sample. Most samples in our study have relatively sparse data in immature whales, especially at young ages. This is due to both sampling bias and the increased probability of smaller whales escaping by-catch or being washed back out to sea during strandings. The absence and unreliability of samples in immature whales prevent us from robustly fitting bathtub-type mortality models (e.g. gompertez-makeham) to our data to estimate survival to maturity. We instead (1) calculate survival to maturity in the samples with high pre-maturity sampling and then (2) use these calculated metrics to estimate survival to maturity in other species-sexes.

1. *Survival to maturity in large samples*

We calculate survival to maturity in the 25 datasets in our sample with greater than 100 whales under the age at maturity (figure SE3). Two of these datasets (both sperm whales) have age distributions clearly biased towards older immature whales and are therefore not included going forwards in this section (figure SE3), leaving a total of 23 datasets.

We fit an extended version of our mortality model to this juvenile mortality data to estimate survival to maturity in these samples. We extend the model by adding a ‘bathtub’ term to the Gompertz model to describe survival before maturity (derived from[18]). Bathtub terms describe a pattern of exponentially decreasing risk of mortality from birth to maturity and are widely applied to mortality models e.g.[18]. We extended our model by replacing the survival term (Equation S1, $L_{d,i}$) in our mortality model with:

$$G_{d,i} = \frac{e^{-\left(\alpha_{SEX_d}/\beta_{SEX_d}\right)\left(e^{\beta_{SEX_d} \times AGE_{d,i}}-1\right)}}{\sum_{j=0}^{n} e^{-\left(\alpha_{SEX_d}/\beta_{SEX_d}\right)\left(e^{\beta_{SEX_d} \times AGE_{d,j}}-1\right)}}$$

$$B_{d.i} = e^{\left(e^{\alpha 0_{SEX_d}}/\beta 0_{SEX_d}\right)\left(e^{-\beta 0_{SEX_d} \times AGE_{d,i}}-1\right)}$$

$$L_{d,i} = G_{d,i} B_{d,i}$$

Where G describes adult mortality as in the unextedned model, and B describes pre-maturity mortality with a bathtub function (defined by parameters $\alpha 0$ and $\beta 0$). All other aspects of the model remained the same, including the corrections for sampling bias, population growth and age estimation error. We fit this model to the 23 datasets with good juvenile data. Some datasets (n = 5) had no whales age 0, in these we assumed that whales of age 0 were just not recorded in the data and therefore only fit the model to ages >0. In other datasets (n = 6) age 1 was overrepresented releative

to age 0 (n whales of age 0 < n whales of age 1)- in these models we assumed there was a sampling bias against whales <1 and modelled this bias using the *S* term in the complete morality model.

Fitting this model results in estimates of Gompertz α0 and β0 for each species-sex. From these parameters, we can estimate the posterior distribution of probabilities of a whale surviving from birth to age at maturity (s0; table SE3).

2. *Inferring s0 in other species*

We then extrapolated from these calculated s0 values to estimate survival to maturity in all toothed whale species.

Survival from birth to maturity (s0) shows a negative relationship with age at maturity in this sample (post. mean = -0.16, 95%CI = -0.32-0.01; figure SE4). The parameters of this relationship are derived from a phylogenetically-controlled Bayesian linear regression. This fitted model includes error around both estimates of the age at maturity and s0. The model does not include a term for sex because of the small sample size but does model phylogenetic autocorrelation via the Ornestain-Uhlenbeck process[19].

We sample from the posterior of this fitted regression to generate distributions of estimates of s0 for all female toothed whale species in our sample (table SE4). For consistency, we use the generated estimates rather than the modelled s0 for all species. These generated estimates have wide distributions, which we view as a strength of the approach because this error is carried through the rest of the analysis. Our calculated demographic parameters, therefore, also inherently incorporate this uncertainty.

Supplementary Data Explanation References

1. McGowen, M. R. *et al.* Phylogenomic Resolution of the Cetacean Tree of Life Using Target Sequence Capture. *Systematic Biology* **69**, 479–501 (2020).
2. Paradis, E. Molecular dating of phylogenies by likelihood methods: A comparison of models and a new information criterion. *Molecular Phylogenetics and Evolution* **67**, 436–444 (2013).
3. Paradis, E. & Schliep, K. ape 5.0: an environment for modern phylogenetics and evolutionary analyses in R. *Bioinformatics* **35**, 526–528 (2019).
4. IWC. Taxonomy: classification of Cetacea. *International Whaling Commission/About Whales* (2021).
5. *Encyclopedia of Marine Mammals*. (Academic Press, 2018).
6. Cáceres-Saez, I. *et al.* Sexual dimorphism and morphometric relationships in pelvic bones of Commerson's dolphins (*Cephalorhynchus c. commersonii*>) from Tierra del Fuego, Argentina. *Marine Mammal Science* **31**, 734–747 (2015).
7. Plön, S. The status and natural history of pygmy (*Kogia breviceps*) and dwarf (*K . sima*) sperm whales off Southern Africa. (2004).
8. Kasuya, T. Some informations on the growth of the Ganges Dolphin with a comment on the Indus Dolphin. *Scientific Reports of the Whales Research Institute* **24**, 87–108 (1972).
9. Negri, M. F. *et al.* Biological parameters of franciscana dolphins, *Pontoporia blainvillei*, by-caught in artisanal fisheries off southern Buenos Aires, Argentina. *Journal of the Marine Biological Association of the United Kingdom* **96**, 821–829 (2016).
10. Ferreira, I. M., Kasuya, T., Marsh, H. & Best, P. B. False killer whales (*Pseudorca crassidens*) from Japan and South Africa: Differences in growth and reproduction. *Marine Mammal Science* **30**, 64–84 (2014).
11. Beasley, I. L. Conservation of the Irrawaddy dolphin, Orcaella brevirostris (Owen in Gray, 1866) in the Mekong River : biological and social considerations influencing management. PhD. (James Cook University, 2007).
12. Robeck, T. R. *et al.* Reproduction, growth and development in captive beluga (Delphinapterus leucas). *Zoo Biology* **24**, 29–49 (2005).
13. Ellis, S. marinelifehistdata: a collection of marine life history data. (2022).
14. Whitehead, H. & Mann, J. Female reproductive strategies of ceteceans: life histories and calf care. in *Cetacean Societies: Field Studies of Whales and Dolphins* (eds. Mann, J., Connor, R. C., Tyack, P. L. & Whitehead, H.) 219–246 (The University of Chicago Press, 2000).

15. *Handbook of marine mammals. Vol. 4: River dolphins and the larger toothed whales*. (Academic Press, 1989).
16. *Handbook of marine mammals. Vol. 5: The first book of dolphins*. (Academic Press, 1994).
17. *Handbook of marine mammals. Vol. 6: The second book of dolphins and the porpoises*. (Academic Press, 1999).
18. Colchero, F., Jones, O. R. & Rebke, M. BaSTA: an R package for Bayesian estimation of age-specific survival from incomplete mark–recapture/recovery data with covariates. *Methods in Ecology and Evolution* **3**, 466–470 (2012).
19. Hansen, T. F. Stabilizing Selection and the Comparative Analysis of Adaptation. *Evolution* **51**, 1341–1351 (1997).
20. Yu, G., Smith, D. K., Zhu, H., Guan, Y. & Lam, T. T.-Y. ggtree: an r package for visualization and annotation of phylogenetic trees with their covariates and other associated data. *Methods in Ecology and Evolution* **8**, 28–36 (2017).
21. Arnold, C., Matthews, L. J. & Nunn, C. L. The 10kTrees website: A new online resource for primate phylogeny. *Evolutionary Anthropology* **19**, 114–118 (2010).
22. Dalebout, M. L. *et al.* Resurrection of Mesoplodon hotaula Deraniyagala 1963: A new species of beaked whale in the tropical Indo-Pacific. *Marine Mammal Science* **30**, 1081–1108 (2014).
23. Thompson, K. *et al.* The world's rarest whale. *Current Biology* **22**, R905–R906 (2012).
24. Jefferson, T. A. & Wang, J. Y. Revision of the taxonomy of finless porpoises (genus Neophocaena): The existence of two species. **4**, (2011).
25. Ben Chehida, Y. *et al.* Mitochondrial genomics reveals the evolutionary history of the porpoises (Phocoenidae) across the speciation continuum. *Sci Rep* **10**, 15190 (2020).
26. Braulik, G. T. *et al.* Taxonomic revision of the South Asian River dolphins (Platanista): Indus and Ganges River dolphins are separate species. *Marine Mammal Science* **37**, 1022–1059 (2021).
27. Jefferson, T. A. & Rosenbaum, H. C. Taxonomic revision of the humpback dolphins (Sousa spp.), and description of a new species from Australia. *Marine Mammal Science* **30**, 1494–1541 (2014).

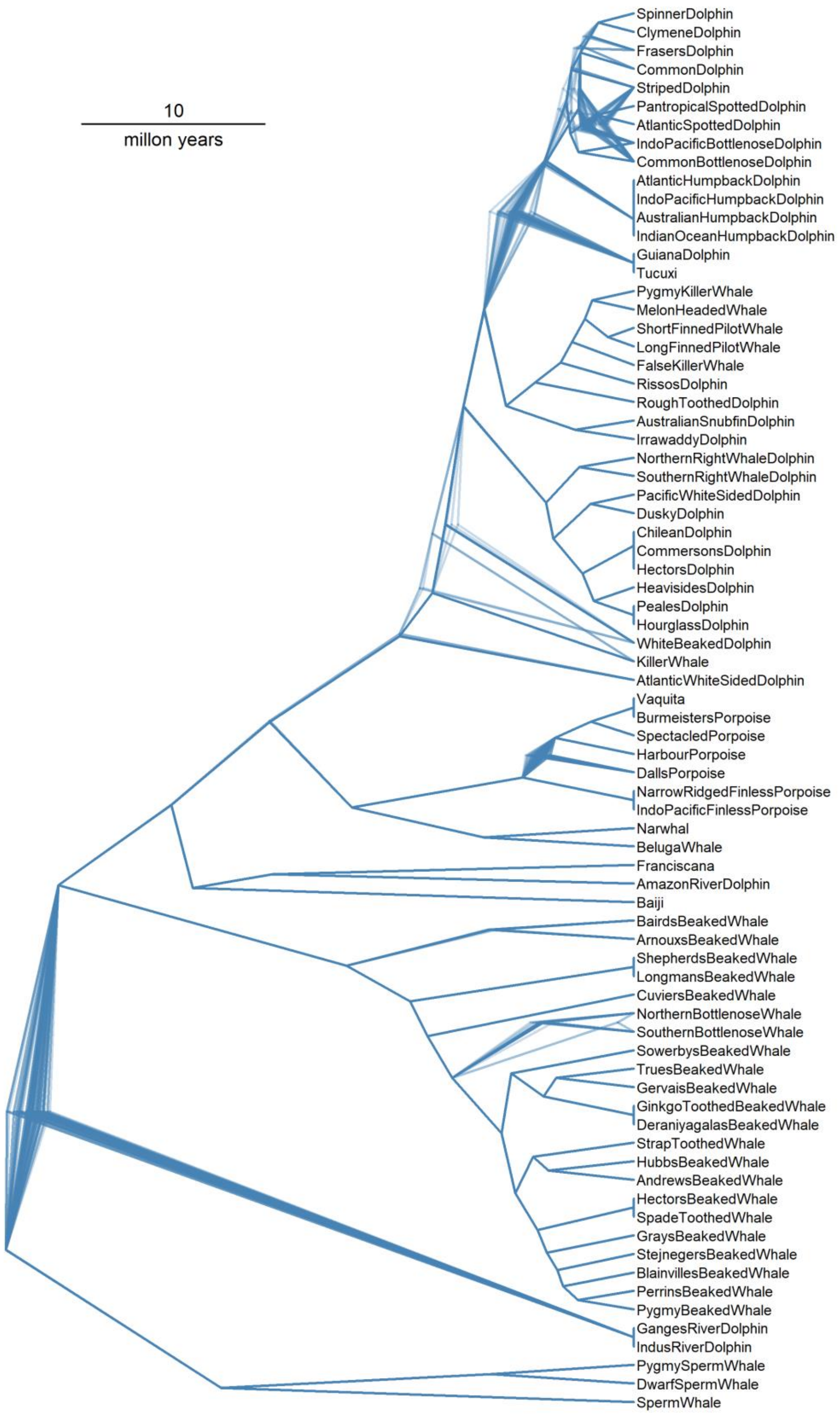

Figure SE1. Complete toothed whale bootstrapped phylogeny used in this study (cf. main text figure 1). The plot shows a randomly selected 100 of 1000 bootstrapped phylogenies. Phylogeny plotted using the ggtree R package[20]. All species names in table SE2.

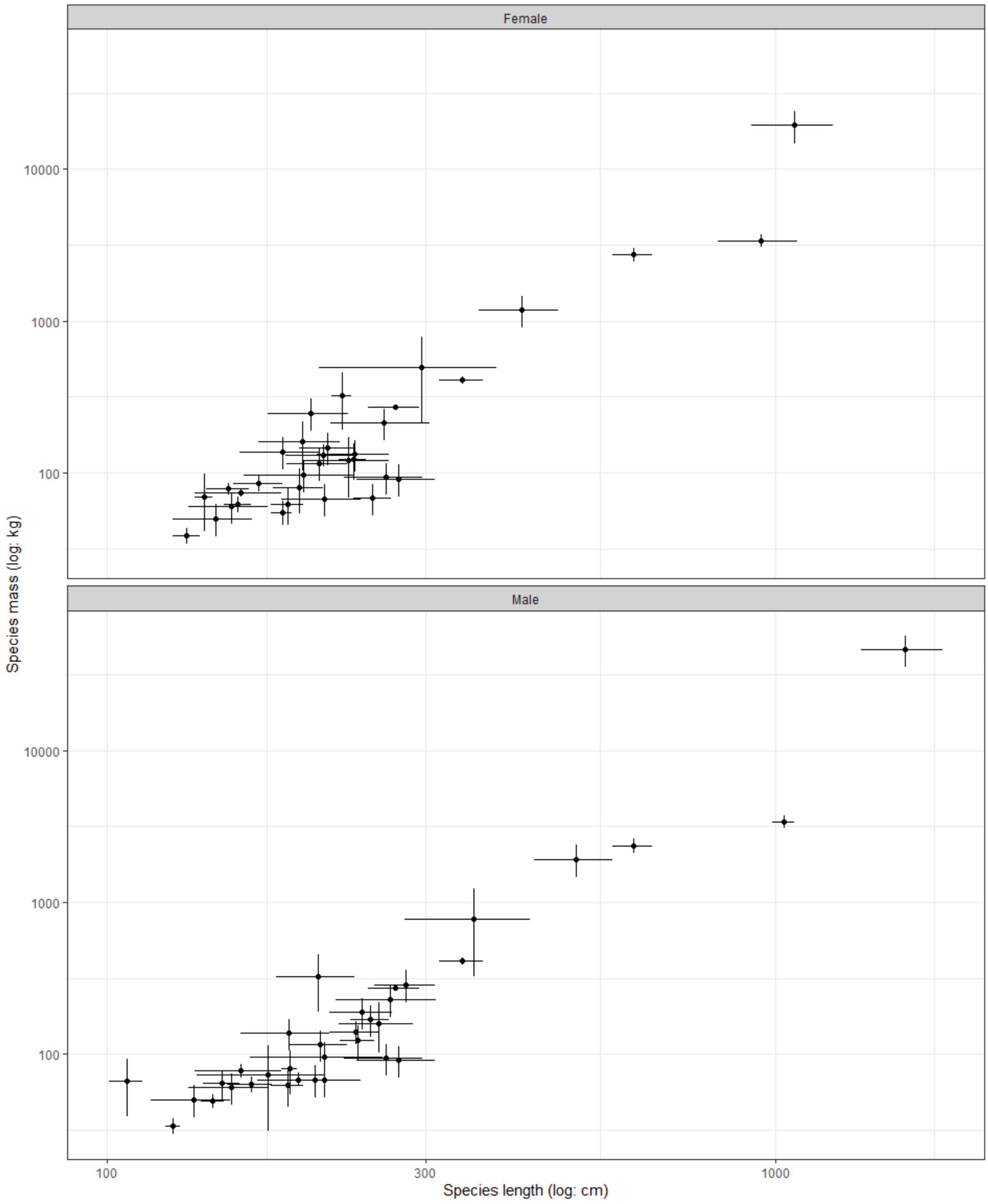

Figure SE2. Comparison of length and size in toothed whales. There is a close linear positive correlation between mass and length in both female (n=34) and male toothed whales (n=34). Each point represents the mean estimate, and errors are standard deviations: both calculated by the pathway described in the text. All data in the marinelifehistory R package[13].

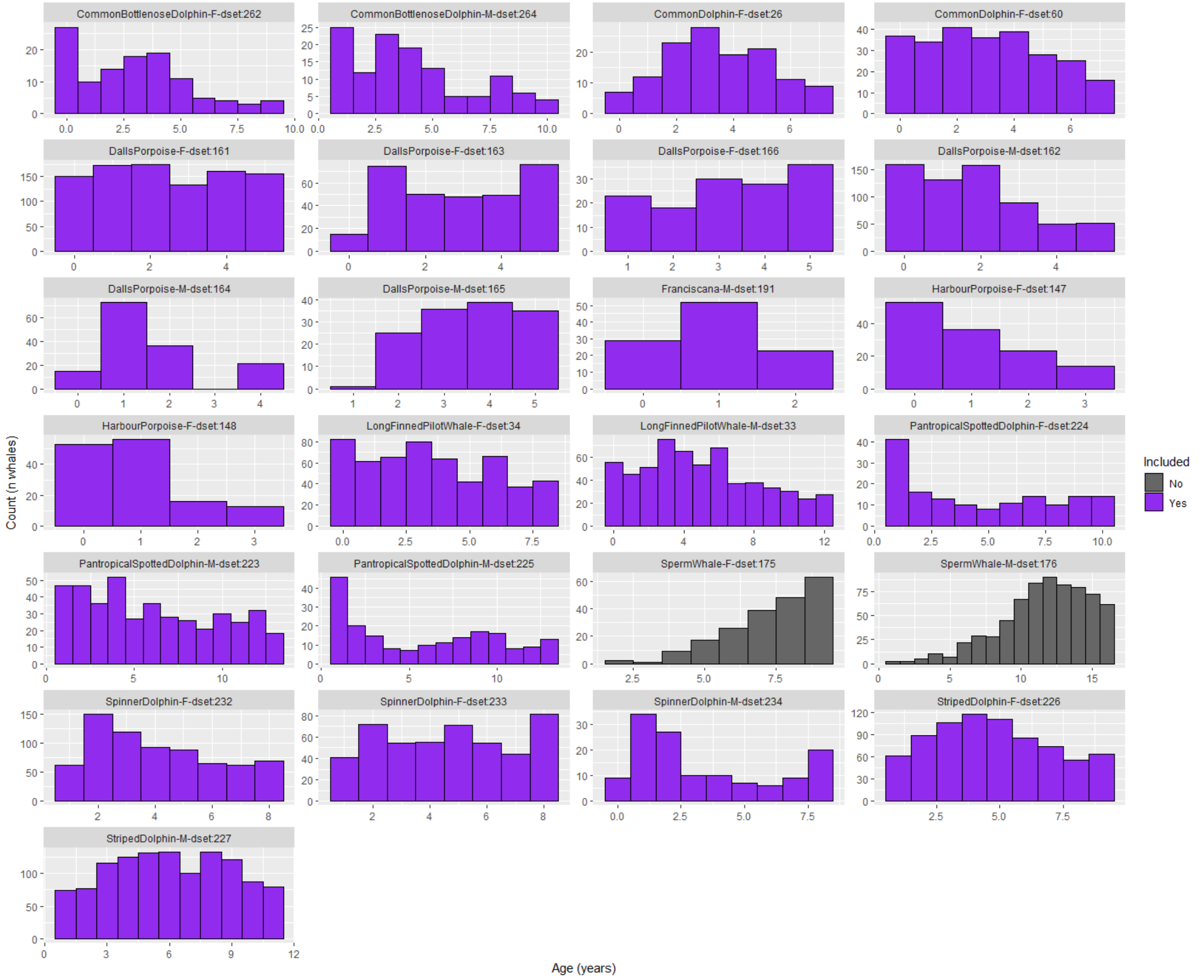

Figure SE3. Pre-maturity age distributions for datasets with greater than 100 pre-maturity samples (n=25). Panel headings define the species-sex-dataset number. Dataset numbers refer to raw datasets in the marinelifehistdata R package[13]. Bar colour indicates if- based on visual examination – the sample is included (purple) or not (grey) in the sample. Samples were excluded if they showed very strong evidence of a bias towards older immature whales.

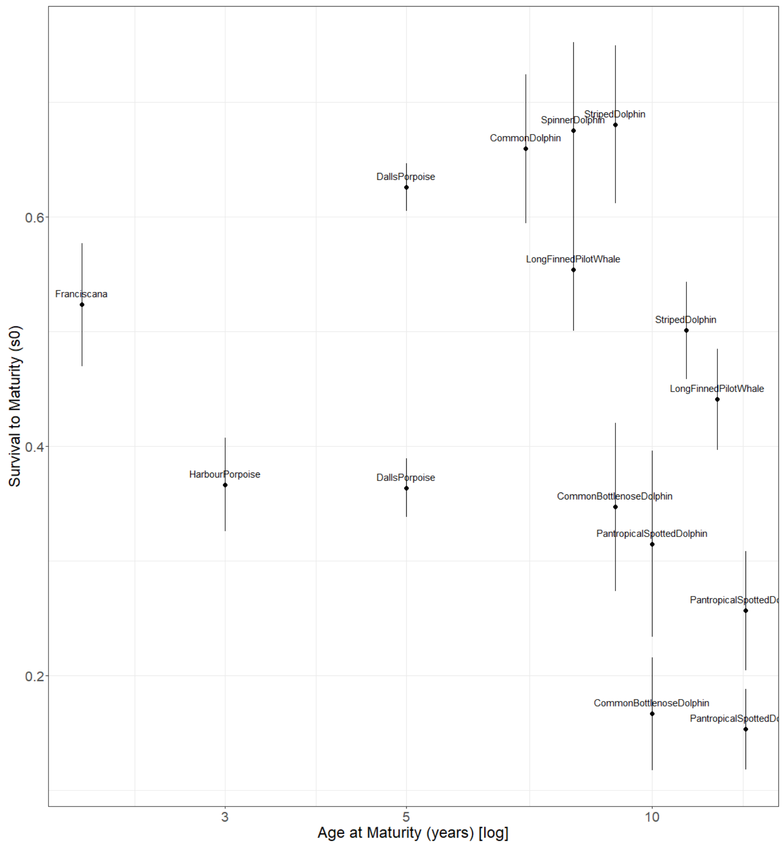

Figure SE4. Relationship between age at maturity and estimate probability of surviving from birth to maturity (s0) in toothed whales (n=15). Points show the mean and standard deviation in both size and s0.

Table SE1. Missing sister species with their selected relatives. For the purposes of our phylogeny, the missing species are assumed to have an identical phylogenetic position to their sister species. * indicate species included in the final lifespan and/or reproductive lifespan samples. Species names in table SE2.

**Missing Species**	**Sister Species**	**Reference**
Chilean dolphin	Commerson's dolphin*	[1]
Hector's dolphin	Commerson's dolphin*	[1]
Longman's beaked whale	Shephard's beaked whale	[21]
Hourglass dolphin	Peale's dolphin	[1]
Deraniyagala's beaked whale	Ginkgo-toothed beaked whale	[22]
Spade-toothed beaked whale	Hector's beaked whale	[23]
Narrow-ridged finless porpoise*	Indo-pacific finless porpoise	[24]
Vaquita*	Burmeister's porpoise	[25]
Ganges river dolphin	Indus river dolphin	[26]
Guiana dolphin*	Tucuxi	[1]
Indian Ocean humpback dolphin	Indo-pacific humpback dolphin	[27]
Australian humpback dolphin	Indo-pacific humpback dolphin	[27]
Atlantic humpback dolphin	Indo-pacific humpback dolphin	[27]

Table SE2. Common and species names of the 75 toothed whale species recognised by the International Whaling Commission and considered in this study[4]. The table is ordered alphabetically by species name.

Common Name	Species Name
**Arnoux's beaked whale**	*Berardius arnuxii*
**Baird's beaked whale**	*Berardius bairdii*
**Commerson's dolphin**	*Cephalorhynchus commersonii*
**Chilean dolphin**	*Cephalorhynchus eutropia*
**Heaviside's dolphin**	*Cephalorhynchus heavisidii*
**Hector's dolphin**	*Cephalorhynchus hectori*
**Beluga whale**	*Delphinapterus leucas*
**Common dolphin**	*Delphinus delphis*
**Pygmy killer whale**	*Feresa attenuata*
**Short-finned pilot whale**	*Globicephala macrorhynchus*
**Long-finned pilot whale**	*Globicephala melas*
**Risso's dolphin**	*Grampus griseus*
**Northern bottlenose whale**	*Hyperoodon ampullatus*
**Southern bottlenose whale**	*Hyperoodon planifrons*
**Longman's beaked whale**	*Indopacetus pacificus*
**Amazon river dolphin**	*Inia geoffrensis*
**Pygmy sperm whale**	*Kogia breviceps*
**Dwarf sperm whale**	*Kogia sima*
**Fraser's dolphin**	*Lagenodelphis hosei*
**Atlantic white-sided dolphin**	*Lagenorhynchus acutus*
**White-beaked dolphin**	*Lagenorhynchus albirostris*
**Peale's dolphin**	*Lagenorhynchus australis*
**Hourglass dolphin**	*Lagenorhynchus cruciger*
**Pacific white-sided dolphin**	*Lagenorhynchus obliquidens*
**Dusky dolphin**	*Lagenorhynchus obscurus*
**Baiji**	*Lipotes vexillifer*
**Northern right whale dolphin**	*Lissodelphis borealis*
**Southern right whale dolphin**	*Lissodelphis peronii*
**Sowerby's beaked whale**	*Mesoplodon bidens*
**Andrews' beaked whale**	*Mesoplodon bowdoini*
**Hubbs' beaked whale**	*Mesoplodon carlhubbsi*
**Blainville's beaked whale**	*Mesoplodon densirostris*
**Gervais' beaked whale**	*Mesoplodon europaeus*
**Ginkgo-toothed beaked whale**	*Mesoplodon ginkgodens*
**Gray's beaked whale**	*Mesoplodon grayi*
**Hector's beaked whale**	*Mesoplodon hectori*
**Deraniyagala's beaked whale**	*Mesoplodon hotaula*
**Strap-toothed whale**	*Mesoplodon layardii*
**True's beaked whale**	*Mesoplodon mirus*
**Perrin's beaked whale**	*Mesoplodon perrini*
**Pygmy beaked whale**	*Mesoplodon peruvianus*
**Stejneger's beaked whale**	*Mesoplodon stejnegeri*
**Spade-toothed whale**	*Mesoplodon traversii*
**Narwhal**	*Monodon monoceros*
**Narrow-ridged finless porpoise**	*Neophocaena asiaeorientalis*
**Indo-Pacific finless porpoise**	*Neophocaena phocaenoides*
**Irrawaddy dolphin**	*Orcaella brevirostris*
**Australian snubfin dolphin**	*Orcaella heinsohni*
**Killer whale**	*Orcinus orca*
**Melon-headed whale**	*Peponocephala electra*
**Spectacled porpoise**	*Phocoena dioptrica*
**Harbour porpoise**	*Phocoena phocoena*
**Vaquita**	*Phocoena sinus*
**Burmeister's porpoise**	*Phocoena spinipinnis*
**Dall's porpoise**	*Phocoenoides dalli*

**Sperm whale**	*Physeter macrocephalus*
**Ganges River Dolphin**	*Platanista gangetica*
**Indus River Dolphin**	*Platanista minor*
**Franciscana**	*Pontoporia blainvillei*
**False killer whale**	*Pseudorca crassidens*
**Tucuxi**	*Sotalia fluviatilis*
**Guiana dolphin**	*Sotalia guianensis*
**Indo-Pacific humpback dolphin**	*Sousa chinensis*
**Indian Ocean humpback dolphin**	*Sousa plumbea*
**Australian humpback dolphin**	*Sousa sahulensis*
**Atlantic humpback dolphin**	*Sousa teuszii*
**Pantropical spotted dolphin**	*Stenella attenuata*
**Clymene dolphin**	*Stenella clymene*
**Striped dolphin**	*Stenella coeruleoalba*
**Atlantic spotted dolphin**	*Stenella frontalis*
**Spinner dolphin**	*Stenella longirostris*
**Rough-toothed dolphin**	*Steno bredanensis*
**Shepherd's beaked whale**	*Tasmacetus shepherdi*
**Indo-Pacific bottlenose dolphin**	*Tursiops aduncus*
**Common bottlenose dolphin**	*Tursiops truncatus*
**Cuvier's beaked whale**	*Ziphius cavirostris*

Table SE3. Probability of surviving from birth to maturity (s0) in toothed whales. Estimates are the posterior: mean, lower 95% credible interval (95lCI), upper 95% credible interval (95uCI) and standard deviation – from a model fitted to datasets with more than 100 whales under the age at maturity.

**Species**	**Sex**	**Age at Maturity**	**s0 mean**	**s0 95%lCI**	**s0 95%uCI**	**s0 std. dev.**
Common bottlenose dolphin	F	9	0.35	0.23	0.51	0.07
Common bottlenose dolphin	M	10	0.17	0.09	0.28	0.05
Common dolphin	F	7	0.66	0.53	0.78	0.06
Dall's porpoise	F	5	0.63	0.59	0.67	0.02
Dall's porpoise	M	5	0.36	0.32	0.42	0.03
Franciscana	M	2	0.52	0.43	0.64	0.05
Harbour porpoise	F	3	0.37	0.29	0.46	0.04
Long-finned pilot whale	F	8	0.55	0.46	0.67	0.05
Long-finned pilot whale	M	12	0.44	0.36	0.54	0.04
Pantropical spotted dolphin	F	10	0.31	0.20	0.52	0.08
Pantropical spotted dolphin	M	13	0.15	0.10	0.23	0.03
Spinner dolphin	F	8	0.68	0.52	0.81	0.08
Spinner dolphin	M	8	0.26	0.18	0.38	0.05
Striped dolphin	F	9	0.68	0.54	0.79	0.07
Striped dolphin	M	11	0.50	0.41	0.57	0.04

Table SE4. Generated estimates of survival from birth to maturity (s0) for toothed whales. Estimates describe the posterior distributions from a Bayesian regression model.

Species	Sex	Age at Maturity (mean)	Age at Maturity (std. dev.)	s0 Mean	s0 std. dev.	s0 95%l CI	s0 u95%CI
**Atlantic Spotted Dolphin**	F	9	0.50	0.42	0.05	0.34	0.52
Atlantic White-Sided Dolphin	F	9	3.00	0.42	0.05	0.34	0.52
Baiji	F	6	1.20	0.49	0.06	0.38	0.60
Bairds Beaked Whale	F	13	2.50	0.34	0.07	0.21	0.49
Beluga Whale	F	10	2.50	0.40	0.05	0.31	0.50
Commerson’s Dolphin	F	5	0.50	0.49	0.06	0.38	0.60
Common Bottlenose Dolphin	F	9	4.00	0.51	0.07	0.39	0.65
Common Dolphin	F	7	1.00	0.42	0.05	0.34	0.52
Dall’s Porpoise	F	5	1.50	0.47	0.05	0.37	0.57
Dwarf Sperm Whale	F	4	1.00	0.40	0.05	0.31	0.50
False Killer Whale	F	10	1.50	0.51	0.07	0.39	0.65
Franciscana	F	2	0.40	0.53	0.08	0.39	0.69
Fraser’s Dolphin	F	6	1.50	0.40	0.05	0.31	0.50
Guiana Dolphin	F	6	1.20	0.58	0.10	0.38	0.76
Harbour Porpoise	F	3	0.50	0.49	0.06	0.38	0.60
Hectors Dolphin	F	7	1.50	0.49	0.06	0.38	0.60
Indian Ocean Humpback Dolphin	F	10	1.99	0.55	0.09	0.39	0.72
Indo Pacific Bottlenose Dolphin	F	12	2.50	0.47	0.05	0.37	0.57
Indo-Pacific Finless Porpoise	F	5	1.00	0.40	0.05	0.31	0.50
Indo- Pacific Humpback Dolphin	F	9	0.50	0.36	0.06	0.24	0.49
Killer Whale	F	13	1.00	0.51	0.07	0.39	0.65
Long-Finned Pilot Whale	F	8	1.59	0.42	0.05	0.34	0.52
Melon-Headed Whale	F	7	1.39	0.34	0.07	0.21	0.49
Narrow-Ridged Finless Porpoise	F	5	1.00	0.45	0.05	0.36	0.54
Narwhal	F	8	0.50	0.47	0.05	0.37	0.57
Northern Bottlenose Whale	F	10	2.50	0.51	0.07	0.39	0.65
Northern Right-Whale Dolphin	F	9	1.79	0.45	0.05	0.36	0.54
Pacific White-Sided Dolphin	F	9	1.50	0.40	0.05	0.31	0.50
Pantropical Spotted Dolphin	F	10	1.00	0.42	0.05	0.34	0.52
Pygmy Sperm Whale	F	5	1.00	0.42	0.05	0.34	0.52
Risso’s Dolphin	F	9	1.00	0.40	0.05	0.31	0.50
Rough Toothed Dolphin	F	10	1.99	0.49	0.06	0.38	0.60
Short-Finned Pilot Whale	F	9	1.79	0.51	0.07	0.39	0.65
Sperm Whale	F	9	1.79	0.42	0.05	0.34	0.52
Spinner Dolphin	F	8	0.50	0.40	0.05	0.31	0.50
Striped Dolphin	F	9	4.00	0.42	0.05	0.34	0.52
Vaquita	F	5	1.50	0.42	0.05	0.34	0.52
White Beaked Dolphin	F	8	1.59	0.45	0.05	0.36	0.54
