# Supplementary material for: The evolution of menopause in toothed whales
Source: Nature. 2024 Mar 13;627(8004):579–85. doi: 10.1038/s41586-024-07159-9 (PMC10954554; doi:10.1038/s41586-024-07159-9)
Supplement: Supplementary file 2 — Reporting Summary [file 41586_2024_7159_MOESM2_ESM.pdf]

nature portfolio

Corresponding author(s): Samuel Ellis

Last updated by author(s): Jan 25, 2024

Reporting Summary

Nature Portfolio wishes to improve the reproducibility of the work that we publish. This form provides structure for consistency and transparency in reporting. For further information on Nature Portfolio policies, see our Editorial Policies and the Editorial Policy Checklist.

Statistics

For all statistical analyses, confirm that the following items are present in the figure legend, table legend, main text, or Methods section.

n/a	Confirmed	
☐	☒	The exact sample size (n) for each experimental group/condition, given as a discrete number and unit of measurement
☐	☒	A statement on whether measurements were taken from distinct samples or whether the same sample was measured repeatedly
☒	☐	The statistical test(s) used AND whether they are one- or two-sided<br>*Only common tests should be described solely by name; describe more complex techniques in the Methods section.*
☐	☒	A description of all covariates tested
☐	☒	A description of any assumptions or corrections, such as tests of normality and adjustment for multiple comparisons
☐	☒	A full description of the statistical parameters including central tendency (e.g. means) or other basic estimates (e.g. regression coefficient) AND variation (e.g. standard deviation) or associated estimates of uncertainty (e.g. confidence intervals)
☒	☐	For null hypothesis testing, the test statistic (e.g. F, t, r) with confidence intervals, effect sizes, degrees of freedom and P value noted<br>*Give P values as exact values whenever suitable.*
☐	☒	For Bayesian analysis, information on the choice of priors and Markov chain Monte Carlo settings
☐	☒	For hierarchical and complex designs, identification of the appropriate level for tests and full reporting of outcomes
☒	☐	Estimates of effect sizes (e.g. Cohen's d, Pearson's r), indicating how they were calculated

Our web collection on statistics for biologists contains articles on many of the points above.

Software and code

Policy information about availability of computer code

Data collection: Rohatgi, A. WebPlotDigitizer. (2020). Is an online tool used to extract data from figures.

Data analysis:
R software. Version 4.05. (2021)
R package: Tidyverse. Version 1.3.0. (2019).
R package: ape. Version 5.0 (2019)
R package: marinelifehistory. Version 0.1.0 (2023).
R package: marinesurvival. Version 0.1.0. (2023)
R package: rstan. Version 2.26.22 (2023)
R package: cmstanr. Version 0.5.3 (2023)
Code and data from this study: github.com/samellisq/marinelifehistdata, github.com/samellisq/marinesurvival, osf.io/26s7m/ .

For manuscripts utilizing custom algorithms or software that are central to the research but not yet described in published literature, software must be made available to editors and reviewers. We strongly encourage code deposition in a community repository (e.g. GitHub). See the Nature Portfolio guidelines for submitting code & software for further information.

Data

Policy information about availability of data

All manuscripts must include a data availability statement. This statement should provide the following information, where applicable:

- Accession codes, unique identifiers, or web links for publicly available datasets
- A description of any restrictions on data availability
- For clinical datasets or third party data, please ensure that the statement adheres to our policy

All data used in this analysis are avalaible as a database at: github.com/samellisq/marinelifehistdata.

Research involving human participants, their data, or biological material

Policy information about studies with human participants or human data. See also policy information about sex, gender (identity/presentation), and sexual orientation and race, ethnicity and racism.

Reporting on sex and gender	NA
Reporting on race, ethnicity, or other socially relevant groupings	NA
Population characteristics	NA
Recruitment	NA
Ethics oversight	NA

Note that full information on the approval of the study protocol must also be provided in the manuscript.

Field-specific reporting

Please select the one below that is the best fit for your research. If you are not sure, read the appropriate sections before making your selection.

☐ Life sciences ☐ Behavioural & social sciences ☒ Ecological, evolutionary & environmental sciences

For a reference copy of the document with all sections, see nature.com/documents/nr-reporting-summary-flat.pdf

Ecological, evolutionary & environmental sciences study design

All studies must disclose on these points even when the disclosure is negative.

Study description	This study uses published age-structured toothed whale life-history data to understand the evolution of menopause in toothed whales.
Research sample	To our knowledge, the sample consists of all published age-structured toothed whale datasets.
Sampling strategy	Sample size was maximised over as many species as possible. Overall it consisted of 269 datasets allowing the calculation of lifespan for 32 species of female and 33 species of male toothed whale.
Data collection	Data were identified in publications through systematic and opportunistic literature searches. Data were then extracted using webplotdigitiser if in a figure or by transcription if in a table.
Timing and spatial scale	Literature search conducted during May-September 2021
Data exclusions	A given species-sex were only used for further analysis if they had at least one dataset with more than 10 samples and a sampling rate (n samples/max observed age) of greater than 0.5/
Reproducibility	There was no experiment in this study.
Randomization	There was no experiment in this study.
Blinding	Blinding was not possible because all data are from species of known identity.

Did the study involve field work? ☐ Yes ☒ No

Reporting for specific materials, systems and methods

We require information from authors about some types of materials, experimental systems and methods used in many studies. Here, indicate whether each material, system or method listed is relevant to your study. If you are not sure if a list item applies to your research, read the appropriate section before selecting a response.

Materials & experimental systems

n/a	Involved in the study
☒	☐ Antibodies
☒	☐ Eukaryotic cell lines
☒	☐ Palaeontology and archaeology
☒	☐ Animals and other organisms
☒	☐ Clinical data
☒	☐ Dual use research of concern
☒	☐ Plants

Methods

n/a	Involved in the study
☒	☐ ChIP-seq
☒	☐ Flow cytometry
☒	☐ MRI-based neuroimaging

Plants

Seed stocks	NA
Novel plant genotypes	NA
Authentication	NA
